# Autophagy and Redox Homeostasis in Parkinson's: A Crucial Balancing Act

**DOI:** 10.1155/2020/8865611

**Published:** 2020-11-10

**Authors:** Natalia Jimenez-Moreno, Jon D. Lane

**Affiliations:** ^1^Edinburgh Cancer Research UK Centre, MRC Institute of Genetics and Molecular Medicine, University of Edinburgh, Edinburgh EH4 2XR, UK; ^2^Cell Biology Laboratories, School of Biochemistry, University of Bristol, Bristol BS8 1TD, UK

## Abstract

Reactive oxygen species (ROS) and reactive nitrogen species (RNS) are generated primarily from endogenous biochemical reactions in mitochondria, endoplasmic reticulum (ER), and peroxisomes. Typically, ROS/RNS correlate with oxidative damage and cell death; however, free radicals are also crucial for normal cellular functions, including supporting neuronal homeostasis. ROS/RNS levels influence and are influenced by antioxidant systems, including the catabolic autophagy pathways. Autophagy is an intracellular lysosomal degradation process by which invasive, damaged, or redundant cytoplasmic components, including microorganisms and defunct organelles, are removed to maintain cellular homeostasis. This process is particularly important in neurons that are required to cope with prolonged and sustained operational stress. Consequently, autophagy is a primary line of protection against neurodegenerative diseases. Parkinson's is caused by the loss of midbrain dopaminergic neurons (mDANs), resulting in progressive disruption of the nigrostriatal pathway, leading to motor, behavioural, and cognitive impairments. Mitochondrial dysfunction, with associated increases in oxidative stress, and declining proteostasis control, are key contributors during mDAN demise in Parkinson's. In this review, we analyse the crosstalk between autophagy and redoxtasis, including the molecular mechanisms involved and the detrimental effect of an imbalance in the pathogenesis of Parkinson's.

## 1. Introduction: Autophagy Forms, Roles, and Regulation

Eukaryotic cells employ a variety of catabolic pathways to degrade altered/damaged proteins and redundant macromolecular components (e.g., organelles). These pathways are critical for cellular homeostasis, and alterations in any have been linked to diverse human diseases [1–4]. Autophagy is one of the major catabolic quality control mechanisms, and is adapted for the degradation of soluble as well as large and/or insoluble cytosolic material, such as aggregated proteins and damaged organelles [1, 2, 5, 6]. It describes several distinct recycling pathways in which cytosolic cargoes are removed through lysosomal degradation, releasing macromolecular precursors such as amino acids, lipids, and nucleosides back to the cytoplasm to be reused. As expected for a process that contributes to removal of toxic cytosolic components, autophagy dysregulation has been linked to numerous diseases, including cancer, bone diseases, cardiomyopathy, infectious diseases, metabolic disorders, and neurodegenerative diseases [7–14]. In this review, we explore the roles of one form of autophagy—macroautophagy—as a prominent pathway for the removal of toxic protein aggregates and damaged organelles, focusing on the interplay between macroautophagy and redox homeostasis, and how imbalances contribute to neuronal decline in Parkinson's.

There are three types of autophagy, each with a distinct mechanism for delivery of substrates to the lysosome. These are microautophagy, chaperone-mediated autophagy (CMA), and macroautophagy. In microautophagy, the cargo is directly engulfed into lysosomes through lysosomal invaginations or protrusions [15]. CMA is a highly selective type of autophagy, where cargoes containing KFERQ-like motifs and/or proteins that have been posttranslationally modified (either by acetylation or phosphorylation) to generate KFERQ-like motifs—becoming de novo CMA substrates [16–18]—are selectively targeted via heat shock cognate 71 kDa protein (HSC70) and cochaperones, and internalized to the lysosome lumen through the lysosome-associated membrane protein 2 receptor (LAMP2A) for their degradation [18]. Thus, CMA plays an important role in the degradation of altered and aggregated proteins, and impairments in this process have been linked to numerous diseases, including neurodegenerative diseases. For example, accumulation of CMA substrates such as *α*-synuclein (*α*-syn) and tau are hallmarks, respectively, of Parkinson's and tauopathies [19].

### 1.1. (Macro)autophagy

**Figure 1:**
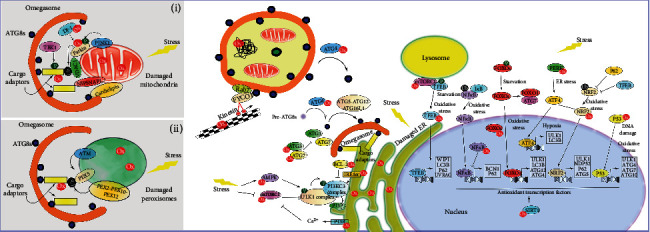
Redox regulation of autophagy. Free radicals in the cell are mainly generated in mitochondria, peroxisomes, and ER; thus, a tightly regulated process to ensure proper functionality and turnover is crucial for cell survival (i.e., degradation by selective autophagy, e.g., mitophagy (i) or pexophagy (ii)). Under certain conditions (e.g., oxidative damage), autophagy is induced as an antioxidant pathway, and this leads to the initiation and nucleation of autophagy assembly sites (e.g., at the ER), with subsequent formation of the autophagosome, and eventual fusion with a lysosome to form a degradative autolysosome. ROS/RNS have the potential to regulate autophagy via upstream regulators, including proteins involved in the UPR system and the autophagy inhibitor mTOR, as well as redox modification in the cytoskeleton, affecting autophagosome transport. In addition, direct modifications in proteins involved in the autophagy process have also been identified including those involved in ATG8 cleavage and conjugation (i.e., ATG4 involved in LC3 cleavage; ATG3 and ATG7 involved in ATG8 lipidation), PI3KC3 activation and cargo recognition (e.g., p62/SQSTM1), and in selective autophagy (e.g., ATM in pexophagy and PINK1, Parkin and DJ-1 for mitophagy) (see the text for full description). Finally, autophagy and redoxtasis crosstalk is evident at the transcriptional level, with several transcription factors involved in autophagy regulation subject to redox modification. Some transcription factors regulate both redox levels and the autophagy process (e.g., NRF2, FOXOs, and p53). P (green): highlights phosphorylation events; Ub (black): highlights ubiquitination events; Ox (red): highlights sites for redox regulation of autophagy.

Macroautophagy is the best understood of the three autophagy forms. It is commonly referred to simply as “autophagy,” and we will adopt the same nomenclature herein. Defects in autophagy are common hallmarks of human diseases, including neurodegenerative diseases [7]. During autophagy, cargoes are sequestered by double-membrane vesicles called autophagosomes, which eventually fuse with lysosomes to generate hybrid degradative compartments (the autolysosomes) (Figure 1) [7, 20–23]. Autophagy is a highly conserved pathway in all eukaryotes, and was first described in detail ~50 years ago by Christian De Duve; however, it was not until the early 1990s that the Nobel Laureate Yoshinori Ohsumi began to unpick the genetic and molecular basis of this process, including identifying the proteins involved and their regulatory interplay using budding yeast [24–26]. Since then, there has been a remarkable progress in this field regarding the molecular control of autophagy and its physiological relevance in multicellular eukaryotes.

Although autophagy occurs in cells under basal conditions, it is dramatically upregulated in response to stresses including starvation, oxidative stress, and pathogen infection [27]. Crucially, autophagy can be nonselective (also known as cargo-independent autophagy), when portions of cytoplasm are randomly encapsulated into autophagosomes based on locality alone, or it can be highly selective. Here, autophagy cargo receptors recognise and bind both cargo and the autophagy machinery, thereby removing specific cargoes such as protein aggregates or damaged organelles [28–31]. Thus, the machineries involved in selective and nonselective autophagy are not identical (e.g., the requirement for specific adaptors and cargo receptors) [32]. In selective autophagy, contributing effector proteins differ depending on specific cargoes, with the process being named according to the organelle affected: mitophagy (mitochondria); pexophagy (peroxisomes); ribophagy (ribosomes); reticulophagy (ER-phagy); lysophagy (lysosomes); xenophagy (bacteria or virus; being distinct from LC3-associated phagocytosis (LAP), where LC3 (see below) is recruited directly to the single-membrane phagosome [33]); nucleophagy (nucleus); proteaphagy (proteasome); lipophagy (lipid droplets); ferritinophagy (ferritin); and glycophagy (glycogen) [28, 34]. Selective autophagy is also implicated in, e.g., noncanonical secretion [35, 36], and LAP for the degradation of bacteria or dead cells [37]. Relevant to a range of human diseases, autophagy also selectively degrades aggregated/misfolded proteins, by a process referred to as aggrephagy [38]. Aggregated proteins that are common hallmarks of neurodegenerative diseases, and known autophagy substrates, include amyloid-*β* [39, 40], that forms amyloid plaques in Alzheimer's disease; HTT (huntingtin) [41], the causative agent in Huntington's disease; and *α*-syn [42], a major component of Lewy's bodies associated with Parkinson's and Lewy's body dementia. Befitting such an important process, a dedicated family of protein is required for autophagy (with the majority designated as “AuTophaGy-related” or “ATG” proteins), and their functions are tightly regulated (a summary of the proteins involved, and their functions, can be found in Table 1) [7, 20–23, 43].

### 1.2. Mechanisms and Regulation of Autophagosome Biogenesis: Initiation and Phagophore Expansion

The process of autophagy consists of several sequential steps: (i) initiation and nucleation; (ii) elongation; (iii) maturation; and (iv) fusion with the endolysosomal compartment. In mammalian cells, autophagy initiation involves the recruitment of several complexes to the autophagy initiation sites and the formation of the phagophore (also known as the isolation membrane). Upon autophagy induction (e.g., nutrient starvation conditions), the Unc51-like kinase 1 (ULK1) complex—formed by the catalytic subunit ULK1, the regulatory subunit ATG13, ATG101, and focal adhesion kinase family interacting protein of 200 kDa (FIP200)—is activated [44]. ULK1 activation depends on its phosphorylation status: (i) it is inactivated by the mammalian target or rapamycin Complex 1 (mTORC1), which also inhibits ATG13 via phosphorylation; and (ii) it is activated by adenosine monophosphate-activated protein kinase (AMPK), which also inhibits mTORC1 directly by phosphorylation, and indirectly via activation of tuberous sclerosis Complex 2 (TSC2) which controls the GTPase activity of the Ras homolog enriched in brain (Rheb) (i.e., Rheb-GDP inhibits mTORC1 activity). The latter process is inhibited in autophagy-inducing conditions [45]. Once activated, ULK1 phosphorylates itself and other ULK1 complex components (i.e., ATG13, FIP200, and ATG101), a step considered important for the catalytic activity of the complex [46–48]. The ULK1 complex is then recruited to the site of autophagosome formation, generally in close-proximity to the endoplasmic reticulum (ER)—or at ER-mitochondria contact sites—triggering nucleation of the phagophore [7, 49–51]. The ULK1 complex activates the downstream machinery including via (i) trafficking of ATG9-positive vesicles from the plasma membrane, recycling endosomes, and trans-Golgi network (TGN) to the autophagy initiation site [52–55]; and (ii) activation by phosphorylation of the autophagic phosphatidylinositol 3-kinase class III Complex 1 (PI3KC3-C1; also known as vacuolar protein sorting 34 Complex 1 (VPS34-I)). This complex comprises (i) the adaptor protein VPS15; (ii) the catalytic subunit VPS34; (iii) ATG14L—required for ER targeting via interaction with syntaxin 17 (STX17) [56]; and (iv) the regulatory subunit coiled-coil myosin-like BCL-2-interacting protein (BECLIN1; itself influenced by AMBRA1 (activating molecule in BECLIN1-regulated autophagy [57])). When active, it establishes phosphatidylinositol 3-phosphate- (PI3P-) enriched subdomains of the ER, known as omegasomes, from where phagophores emerge [49, 58–60].

At the omegasome, PI3P effector proteins are recruited, including the zinc-finger FYVE domain-containing protein (DFCP1), the autophagy-linked FYVE protein (ALFY), and WD repeat domain phosphoinositide-interacting proteins (WIPIs; here WIPI2 is the exemplar isoform) [7]. DFCP1 resides on ER/Golgi membranes, and is an excellent omegasome marker, but is thought not to be essential for autophagy [58]. ALFY has been reported to be essential for selective degradation of aggregated proteins, and is required for neuronal connectivity [61, 62]. WIPI2 plays an important role in the recruitment and activation of the tandem Ub-like (UBL) conjugation pathways that drive autophagosome assembly, namely, the ATG12 and the ATG8 conjugation systems [43, 63, 64]. In the first UBL conjugation system, the UBL protein ATG12 is conjugated to ATG5 by a process mediated by ATG7 (E1-like activating enzyme) and ATG10 (E2-like conjugating enzyme). ATG12~5 binds to ATG16L1, generating a complex with E3-like activity for the second UBL conjugation pathway [65, 66]. ATG12~5-16L1 complex recruitment is mediated by direct interactions between WIPI2B (WIPI2 splice variant) and ATG16L1 [63]. There, the AT12~5-16L1 complex, together with ATG3 (E2-like conjugating enzyme) and ATG7, coordinates activities in the second UBL conjugation pathway, during which ATG8 family members are covalently attached to lipids (most often to phosphatidyl ethanolamine (PE)) in situ. The ATG8 family comprises the microtubule associated protein 1 light chain 3 (MAPLC3; herein, referred to as LC3) and gamma-aminobutyric acid receptor-associated protein (GABARAP) families. These families encompass LC3A (with two variants differing in the N-terminal sequence, v1 and v2 [67]), LC3B (LC3B1 and LC3B2, with only one amino acid difference (C^113^ versus Y^113^ [67]), LC3C, GABARAP, GABARAPL1, GABARAPL2/GATE-16 [68]. Prior to lipidation, ATG8s are first activated (or primed) by members of the ATG4 endopeptidase family (ATG4A-D; with ATG4B being the best characterised family member which displays activity against all ATG8s), which cleave ATG8 proteins at their C-termini to expose a glycine residue (e.g., G120 in LC3B) that is the future site for lipidation (e.g., primed LC3 is referred to as “LC3-I”) [7, 22, 69]. The subsequent covalent attachment of ATG8s to lipids at the nascent isolation membrane generates the membrane-bound form (e.g., LC3-II) [70, 71], a step that is followed by coordinating membrane expansion and phagophore closure.

### 1.3. Mechanisms and Regulation of Autophagosome Biogenesis: Maturation, Trafficking, and Lysosomal Fusion

Autophagosome maturation and fusion with lysosomes involves (i) membrane fission for autophagosome closure; (ii) trafficking of the autophagosome along the cytoskeleton, typically in the retrograde direction (i.e., towards the centre of a typical cell); and finally (iii), fusion with the lysosome to form a degradative autolysosome [43]. Here, cargoes are degraded, and their components transported back into the cytosol. ESCRT proteins (endosomal sorting complex required for transport) have been identified as essential for autophagosome membrane fission/closure. These are recruited in a RAS-related protein 5- (RAB5-) dependent manner [72]. Defects in LC3B lipidation have been found to cause the accumulation of unclosed autophagosomes, suggesting that the ATG-conjugation machinery is needed for this process [73]. Despite this, functional autophagosomes do form in the absence of all ATG8 family members [74]. Other interventions that lead to the accumulation of unsealed autophagosomes in the cytoplasm include knockdown of the phospholipid transfer protein ATG2A/B, required for autophagosome expansion [75]. Indeed, it was recently shown that an ATG2-GABARAP interaction is needed for efficient autophagosome closure [76].

Fully formed autophagosomes go through a maturation process during which ATG8 proteins link autophagosomes to motor proteins and the microtubule cytoskeleton. For example, RAB7 is recruited to mature autophagosomes and further recruits the FYVE and coiled-coil domain-containing 1 protein (FYCO1), which in turn binds to LC3 (via LIR- (LC3-interacting region-) type interaction) and PI3P to mediate anterograde kinesin-driven transport [77]. Alternatively, RAB7 binds to the RAB-interacting lysosomal protein (RILP) to mediate retrograde dynactin/dynein-driven transport towards the nucleus [78–82]. Crucially, microtubules are also involved in autophagosome formation (e.g., microtubule transport from the centrosome is necessary for recruitment of GABARAP to the nascent phagophore via the centriolar satellite protein PCM1 [80, 83]).

For membrane fusion and formation of the autolysosome, the SNARE (soluble N-ethylmaleimide-sensitive factor activating protein receptor) fusion machinery is required. On the autophagosome membrane resides STX17 and synaptosomal-associated protein 29 (SNAP29), whereas on the lysosome, vesicle-associated membrane protein 7 or 8 (VAMP7 or VAMP8) mediates membrane fusion supported by the homotypic fusion and protein sorting complex (HOPS) which interacts with STX17 [84, 85]. STX17 also recruits ATG14L (also involved in autophagosome formation as a complex of PI3KC3-C1) to promote membrane tethering and to stabilise the SNARE complex promoting membrane fusion [86–88]. In addition, Wilkinson et al. described that phosphorylation of LC3B by hippo kinases STK3 and STK4 was critical for autophagosome fusion [89]. Meanwhile Wang et al. described that ULK1 mediates autophagosome-lysosome fusion via interactions with STX17, with protein kinase *α*- (PKC*α*-) mediated ULK1 phosphorylation reducing this interaction via ULK1 degradation by the CMA pathway [90].

### 1.4. General Features and Properties of Autophagy in Neurons

Autophagy pathways in general are especially important in neurons, as these are postmitotic cells that cannot dilute cytoplasmic damage through proliferation/division, and thus autophagy is required to maintain long-term neuronal functionality. Although this review focuses on (macro)autophagy, it is important to mention that CMA and microautophagy are also present in neurons [91]. In neurons, autophagy is needed to degrade neurotoxic factors (e.g., *α*-syn) and damaged organelles that are selected by ubiquitylation and recognised by the autophagy machinery [92, 93]. Misfolded proteins can be refolded by the actions of chaperones, or can be degraded primarily by the ubiquitin-proteasome system (UPS); however, when these processes are impaired, misfolded proteins accumulate to form aggregates (aggresomes) that require removal via aggrephagy [94], otherwise insoluble inclusions are generated [95, 96]. These are defining features of some neurodegenerative diseases, including Parkinson's [2]. In autophagy-deficient dopaminergic neurons (mDANs), derived from *Atg7* knockout mice, *α*-syn and p62/SQSTM1 both accumulate in inclusions within neurites in an ageing-dependent fashion that is ultimately linked to mDAN loss and motor dysfunction [96].

Autophagy also contributes to axonal regeneration, presynaptic modelling, dendritic spine pruning, and synaptic plasticity [91, 97–100]. Autophagy dysregulation has been linked to the development of neurodegenerative diseases [101–103], and crucially, decreased autophagic activity is a characteristic of ageing [8]. Autophagy supports neuronal survival. For example, neonatal lethality in *Atg5* knockout mice is rescued after restoration of neuronal-specific expression of ATG5 [104]; meanwhile, autophagy activation in the mouse brain protects against mDAN loss mediated by oxidative stress [105], and autophagy induction using a neuronal pharmacophore in amyotrophic lateral sclerosis (ALS) and Huntington's mouse models promotes neuronal survival [106]. Consistent with this, suppression of basal autophagy also causes neurodegeneration. For example, conditional neuronal autophagy deficiency leads to neuronal loss, and mutations in autophagy genes have been linked to several neurodegenerative disorders [103, 107–109].

Previous work in primary rodent neurons points to unique characteristics of autophagosome assembly, maturation, and trafficking in these specialised cells [97, 110]. Importantly, control and substrate targeting appear to differ depending on neuronal cell-type and specific conditions. For example, in neurons of the dorsal root ganglia (DRG), autophagy is triggered almost exclusively at the distal tip, whereas in hippocampal primary neurons, it can be initiated in the cell body, dendrites, and axonal regions proximal to the cell body [111, 112]. However, under stress conditions, mitophagy initiation has been reported to also occur along the axon [113]. In general, autophagosome biogenesis is initiated primarily in the distal axon, and thereafter, autophagosomes undergo dynein-dependent retrograde motility to the lysosome-rich soma following recruitment of neuronal scaffold proteins such as JIP1 [112, 114]. Overall, it is essential that neuronal subtype specification is considered when attempting to generalise about the roles and regulation of autophagy in the brain.

Autophagosome biogenesis is a constitutive process that can be triggered in the soma or distal axon where elements of the core autophagy machinery are actively recruited (e.g., ATG9A-containing vesicles are transported from the soma to the distal axons via the kinesin family member, KIF1A [115]). Supplementing these, several neuron-specific proteins have been reported to be involved in autophagosome biogenesis and maturation (e.g., synaptojanin, endophilin A, Basson, and Piccolo) (Table 1). The presynaptic proteins, endophilin A and synaptojanin (mutations in *SYNJ1* are associated with Parkinson's [116]), are primarily involved in the recycling of synaptic vesicles [117], but have also been shown to mediate ATG3 recruitment to the nascent phagophore [118] and to promote autophagosome maturation [119], respectively. Basson and Piccollo are two proteins involved in active zone assembly for the release of neurotransmitters, and they have each been found to act as autophagy inhibitors by sequestering ATG5 [120]. Autophagosome biogenesis can also occur in dendrites (or alternatively, autophagosomes can also migrate from the soma to the dendrites), and here autophagy activity increases as a function of synaptic activity [111, 121]. Furthermore, recent studies have suggested the existence of an unconventional degradation pathway in which glial cells modulate neuronal autophagy by intercellular regulation and/or direct transfer of cellular garbage from neurons, an idea that builds on previous data supporting autophagosome secretion in nonneuronal cells [97, 122].

Neuronal autophagy properties also appear to vary as a function of ageing, with accumulation of neuromelanin and lipofuscin progressively observed in autophagosomes in aging brain tissues [123]. Overall, autophagosome biogenesis efficiency is seen to decline in aged neurons [124].

## 2. Selective Autophagy and Its Relevance to Neurodegenerative Diseases

As a key component of cellular and tissue homeostasis, with protective roles in human neurodegenerative diseases, a full appreciation of mitophagy regulatory control in neurons is desirable. In particular, mitophagy dysfunction is a hallmark of Parkinson's, implicated in a number of early onset genetic forms [125], and is observed in genetic and toxin-induced Parkinson's models [126]. Distinct mechanisms and diverse proteins are involved in the selective degradation of mitochondria, and these have been reviewed in detail elsewhere [32, 103]. Mitochondria can be damaged by numerous factors, including hypoxia, mtDNA damage, chemical uncouplers that dissipate membrane potential (e.g., carbonyl cyanide m-chlorophenylhydrazone (CCCP)), electron transport complex (ETC) inhibitors (e.g., rotenone (Complex I inhibitor) or antimycin (Complex III inhibitor)), or the presence of reactive oxygen species (ROS; mitochondrial superoxide production), as will be described in detail later [127–129]. Thereafter, differing fates are observed, with damaged mitochondria either being rescued by fusion/fission [130] or being degraded via mitophagy.

### 2.1. The PINK1/Parkin Mitophagy Pathway

The best characterised route for mitochondrial degradation is via the PINK1/Parkin (PRKN) pathway, although several Parkin-independent pathways have been described [131–135]. Crucially, *PINK1* and *PRKN* mutations are linked to familial Parkinson's [9]. When mitochondrial membrane potential is intact, PINK1 is imported into mitochondria via the TOM/TIM23 system (translocase of the outer membrane and inner membrane, respectively), to be cleaved consecutively by the matrix-localized protease (MPP) and presenilin-associated rhomboid-like protease (PARL) [128, 136]. However, when membrane potential is lost (i.e., as a feature of damaged mitochondria), PINK1 accumulates on the mitochondrial outer membrane where it phosphorylates and activates Parkin (an E3 ligase), driving protein ubiquitylation on the outer mitochondrial membrane. Subsequently, PINK1 phosphorylates target-bound ubiquitin which in turn recruits further Parkin in a positive feedback pathway [137]. Parkin targets mitochondrial surface proteins, such as the voltage-dependent anion channel 1 (VDAC1) [138]. For Parkin recruitment and substrate ubiquitination, an interaction with the Parkinson's-linked protein F-box protein 7 (FBXO7) is involved, although the precise molecular mechanism remains elusive [139]. These ubiquitylated proteins are recognised by cargo receptor proteins, and thereafter ubiquitylated mitochondria are targeted to the nascent phagophore. Zachari et al. suggested that ubiquitylated mitochondria are enveloped by ER strands to facilitate targeting and autophagy [140].

### 2.2. Cargo Receptors and Their Roles in Mitophagy, Pexophagy, and ER-Phagy

There are several cargo receptor proteins involved in mitophagy, including p62/SQSTM1, NIX (or BNIPL3), Neurabin-1 (NRB1), FUNDC1 (FUN14-domain-containing 1), NDP52, Optineurin (OPTN), and Tax1 binding protein 1 (TAX1BP1) [127]. Recruitment of these receptor proteins occurs subject to specific regulation. For example, receptor binding affinity (particularly OPTN and p62/SQSTM1) is increased via phosphorylation by tank-binding kinase 1 (TBK1) [141, 142]. These receptors bind to ATG8s (via LIR-type interactions) [55], and some of them also recruit the ULK1 complex in a feed-forward pathway to reinforce the autophagosome assembly machinery [143, 144]. Subsequently, mitochondria are degraded by the (macro)autophagy pathway. Ubiquitin is not the only targeting signal for mitophagy, as it has been recently described that the mitochondrial matrix proteins 4-nitrophenylphosphatase domain and nonneuronal SNAP25-like protein homolog (NIPSNAP1) and NIPSNAP2 accumulate on the mitochondrial surface to act as “eat-me” signals through binding to mitophagy cargo receptors [135]. In addition, it has been observed in neurons that cardiolipin externalization on the mitochondrial surface triggers mitophagy via interactions with LC3, thereby targeting mitochondria for degradation [134, 145, 146].

Some cargo receptors involved in mitophagy—including NDP52, OPTN, NRB1, and p62/SQSTM1—also facilitate the degradation of protein aggregates (aggrephagy), or other organelles such as peroxisomes (pexophagy) [147, 148]. Peroxisomes are small single-membrane organelles involved in lipid synthesis and redox homeostasis. Thus, pexophagy is crucial for peroxisome quality control and turnover [149]. In this process, peroxisome membrane proteins, including the peroxisomal biogenesis factor (PEX) 5 and 70 kDa peroxisomal membrane protein (PMP70), are ubiquitylated by the E3-like ubiquitin ligase complex PEX2-PEX10-PEX12, facilitating recognition by cargo receptors and degradation via autophagy [149]. Alternatively, to prevent pexophagy, the deubiquitinase USP30 and the AAA-type ATPase (PEX1-PEX6-PEX26) remove ubiquitylated membrane proteins. Conversely, peroxisomal dysfunction are linked to peroxisome biogenesis disorders. However, the effect of altered pexophagy in neurodegenerative diseases is poorly understood [150].

Other specific proteins implicated in the selective autophagy of different organelles include the following LIR-motif-containing proteins involved in ER-phagy: CCPG1 (cell cycle progression protein 1), FAM134B (family with sequence similarity 134 member B); ATL3 (atlastin 3), SEC62 (secretory 62 homolog), CALCOCO1 (calcium-binding and coiled-coil domain 1), RTN3 (reticulon 3), and TEX264 (testis-expressed protein 264); they are found in different regions of the ER, and they might have different roles and be tissue-specific [151–160]. The ER is a complex organelle that mediates protein folding, processing and transport in the secretory pathway, calcium storage, lipid synthesis, and intracellular signalling via interactions with other organelles. In common with other organelles, the ER is also subject to turnover and remodelling to ensure proper and optimal functional plasticity [161, 162]. The best characterised network for ER remodelling is the unfolded protein response (UPR), triggered by the presence of lumenal misfolded proteins, with the consequent cytosolic signalling cascades originated by ER-sensing proteins: inositol-requiring enzyme 1*α* (IRE1*α*), protein kinase RNA-like ER kinase (PERK), and activating transcription factor 6 (ATF6). These cascades trigger the translational and transcriptional regulation of redox enzymes, chaperones, foldases, lipid synthesis proteins, autophagy-related proteins (e.g., CCPG1 [152]), and ERAD (ER-associated degradation) genes involved in proteasomal degradation [163].

Under nutrient starvation or ER stress (i.e., lumenal misfolded proteins), ER-phagy is induced via different pathways after UPR activation. Calcium released via the inositol trisphosphate receptor IP3R and other calcium channels activates calcium-dependent proteins, namely, calmodulin-dependent protein kinase (CAMKK), which inhibits mTORC1 [164, 165]; death-associated kinase (DAPK) and DAPK2 which regulate BECLIN1 activation and mTOR inhibition, respectively, [166–168]; and CAMK2B, which phosphorylates FAM134B, promoting its oligomerization [169]. On the other hand, IRE1*α* indirectly activates BECLIN1, thus promoting autophagy initiation, while PERK and ATF6, respectively, activate two autophagy transcription factors, ATF4 (activating transcription factor 4) and CHOP (C/EBP homologous protein) [170, 171] (for a detailed overview, see [172]). ER-phagy can be classified as macro-ER-phagy (commonly referred as “ER-phagy”), where fragments of ER are sequestered into an autophagosome which later fuses with the lysosome, and micro-ER-phagy, when a fragment of the ER is directly engulfed and targeted to the lysosome (for recent reviews, see [173, 174]). Recently, numerous human ER-phagy regulators have been identified in a genome-wide screening after starvation [159], and recent data highlight the importance of ER-phagy in cell survival, with defects in this process being related to infectious diseases and cancer development and progression (for a review, see [175]).

In neurons, the ER extends from the cell body and along the axon to the axonal distal tip. It is crucial for neuronal function (particularly the regulation of the neuronal calcium homeostasis), and the ER tubular network is disrupted in several neurodegenerative diseases [176–178]. Consistent with this, UPR has been recently implicated in memory, synaptic plasticity, dendritic outgrowth and branching, and axonal regeneration [179–182]. In addition, previous studies have highlighted the importance of ER-phagy in neurons. For example, (i) FAM134B deficiency in primary neurons leads to progressive ER stress and affects the survival of sensory neurons [156]; (ii) *RTN3* is linked to AD [183]; and most recently, (iii) Park et al. described that induction of ER stress and consequent ER-phagy is involved in early stages of hypothalamic development and metabolic regulation [184]. However, the role of ER-phagy in neuronal homeostasis and neurodegenerative diseases remain to be fully explored.

## 3. Redox Homeostasis

Reactive oxygen species (ROS) (e.g., hydrogen peroxide (H_2_O_2_) and superoxide (O_2_^•-^)) and reactive nitrogen species (RNS) (e.g., nitric oxide (^•^NO)) are highly reactive molecules generated under both basal and pathological or stress conditions (for a detailed description of free radicals see [185]). They are involved in numerous pathologies, including Parkinson's, Alzheimer's, ALS, diabetes, cancer, and autoimmune disorders [186]. These radicals are important for cellular homeostasis, regulating several cellular functions including cell signalling, proliferation, and survival in response to stress or injury. Reduction and oxidation reactions, where there is a transfer of electrons between chemical species—also known as redox reactions—are focused at the mitochondria, peroxisomes, and ER, although there are additional contributions from alternative organelles depending on the cell type [187, 188]. In addition, cells have different inherent antioxidant mechanisms to control ROS/RNS levels and avoid/alleviate toxicity. Oxidative stress occurs when antioxidant mechanisms are not sufficient, and ROS/RNS levels accumulate, ultimately impacting on normal biological processes and limiting cell survival. Due to the high oxygen demands and lipid contents in the brain, neurons are particularly sensitive to oxidative stress, with some areas being more susceptible than others (e.g., the hippocampus) [189]. For this reason, high levels of oxidative stress are one of the main hallmarks of neurodegenerative diseases, including Parkinson's, aggravating the disorder by affecting protein aggregation, DNA damage, and ultimately, causing neuronal cell death.

### 3.1. Sources and Causes of Redox Imbalance

Mitochondria are the major source of cellular ATP, generated via the electron transport chain (ETC), comprising (i) Complex I (NADH dehydrogenase), which uses NADPH generated in the citric acid cycle for proton translocation from the mitochondrial matrix to the intermembrane space, with electrons being transferred to ubiquinone; (ii) Complex II (succinate dehydrogenase), which uses flavin adenine dinucleotide (FADH_2_) generated from succinate in the citric acid cycle and consequently delivers electrons to the ETC (ubiquinone); (iii) Complex III (cytochrome *c* oxidoreductase), where electrons (from ubiquinone) are transferred to cytochrome *c*; (iv) Complex IV (cytochrome *c* oxidase), where electrons are removed from cytochrome *c* to generate H_2_O with energy released used to translocate protons to the intermembrane space; and (v) Complex V (ATP synthase), for the generation of ATP via proton flow to the matrix (at a ratio of 4H+ : 1ATP) [190]. Complexes I, II, and III are among the major ROS production enzymes in the cell, generating O_2_^•-^ due to electron leakage [191]. In addition, glycerol-3-phosphate dehydrogenase, which catalyses the conversion of glycerol-3-phosphate to dihydroxyacetone phosphate and the generation of FADH_2_ while transferring electrons to ubiquinone in the ETC, generates additional O_2_^•-^. Similarly, also in the inner mitochondrial membrane, the electron transfer to flavoprotein, ubiquinone oxidoreductase, and dihydroorotate dehydrogenase, that, respectively, links fatty acid *β*-oxidation and pyrimidine biosynthesis to electron transfer to the ETC, also generates O_2_^•-^ [192]. The other major source of ROS in mitochondria is the Krebs cycle (or citric acid cycle). This metabolic pathway is performed by aerobic organisms in the mitochondrial matrix, and consists of a series of chemical reactions for the production of ATP, alongside reduced forms of NADH and FADH_2_ to be used in the ETC. Particularly, dihydrolipoamide dehydrogenase (DLD), an E3 component of pyruvate dehydrogenase (for the production of acetyl-CoA from pyruvate), and *α*-ketoglutarate dehydrogenase (catalyses the conversion of *α*-ketoglutarate to succinyl-CoA, producing NADH), generates unwanted O_2_^•-^ via the flavin cofactor of this enzyme. Finally, superoxide dismutase 2 (SOD2) in the mitochondrial matrix, and SOD1 in the intermembrane space, convert O_2_^•-^ into H_2_O_2_, which can potentially be turned into ^•^OH radicals via the Fenton reaction. Mitochondria are also sources of RNS, including ^•^NO that is produced by nitric oxide synthases (NOS) in the oxidation of L-arginine [188, 193]. Peroxisomes are oxidative organelles involved in lipid metabolism of long-chain and branched fatty acids via fatty acid *β*-oxidation, lipid synthesis, purine catabolism, and amino acid and glyoxylate metabolism. Importantly, most enzymes involved in these processes produce ROS. For example, acyl-coA oxidase(s), which catalyse the first step in peroxisomal fatty acid *β*-oxidation, generates H_2_O_2_ [194]. Similarly, xanthine oxidase, cleaved from xanthine dehydrogenase in response to an increase in calcium extracellular levels (e.g., hypoxia) and involved in the purine metabolism to uric acid, generates O_2_^•-^ and H_2_O_2_ [195, 196]. During peroxisomal amino acid metabolism, D-amino acid oxidase (catalyses oxidation of D-amino acids to imino acids) and the L-pipecolic acid oxidase (involved in lysine degradation) generate H_2_O_2_. Other peroxisomal enzymes producing ROS/RNS include L-*α*-hydroxyacid oxidase (involved in oxidation of glycolic acid), polyamine oxidase (involved in polyamine degradation), sarcosine oxidase (metabolises sarcosine, L-pipecolic acid, and L-proline), D-aspartate oxidase (catalyses oxidation of D-aspartate), SOD1, and NOS2 [197, 198].

The ER is involved in diverse functions including protein folding, processing and vesicular transport, calcium storage, lipid synthesis, cell signalling, and xenobiotic toxicity. Particularly, during protein folding, ER oxidoreductin (ERO1) catalyses oxidation of protein disulfide isomerase, involved in disulfide bond formation, generating H_2_O_2_. Similarly, quiescin sulfhydryl oxidase, also present in the Golgi, generates H_2_O_2_ for the introduction of disulfide bonds into unfolded reduced proteins and can compensate for the loss of ERO1 [199, 200]. The other main source of H_2_O_2_ in the ER is the NADPH oxidase 4 (NOX4). Proteins that belong to the NOX family are the only cellular enzymes exclusively involved in the production in ROS by using NAD(P)H for oxygen reduction to produce a superoxide anion [197]. Finally, the microsomal monooxygenase (MMO) system, composed of cytochrome P450 (P450), NADPH-P450 reductase (NPR), and phospholipids, is involved in the oxygenation of several exogenous (xenobiotics) and endogenous substrates (e.g., heme oxygenase and fatty acid desaturase), and is one of the major sources of ROS in the ER via electron leakage from P450 [199].

Other sources of cellular ROS include the plasma membrane and lysosomes, as well as cytosolic reactions [188]. In addition, ROS production can also be induced in response to hypoxia (by acting on the mitochondrial ETC and increasing intracellular calcium levels [196, 201, 202]) and starvation, and more generally following environmental stress (e.g., paraquat), infections, physical exercise, and mental stress; and increased ROS/RNS levels have been observed during aging [203–205].

### 3.2. Dual Roles of ROS and RNS

#### 3.2.1. Beneficial Activities of Free Radicals: Oxidative Eustress

Crucially, ROS and RNS are not only detrimental to cells, but they are also important for cellular homeostasis, regulating numerous important cellular activities, also known as physiological oxidative stress or oxidative eustress. ROS and RNS act as second messengers in signal transduction pathways involved in cell survival, cell to cell communication, and cell growth and proliferation [206–208]. They influence diverse signalling pathways via oxidation of cysteine sulfhydryl groups in protein kinases, including protein kinase A (PKA), PKC, receptor tyrosine kinase (RTK), and Ca^2+^/calmodulin independent protein kinase II (CaMKII). Other pathways that display crosstalk with ROS/RNS include the NF-*κ*B pathway, the MAPK pathway, the PI3K/AKT pathway, ATM signalling, the insulin pathway (e.g., oxidation of protein tyrosine phosphatase 1B (PTP1B)), iron metabolism (e.g., Fenton reaction), calcium signalling (e.g., oxidation of Ca^2+^ channels, pumps, and exchangers), the ubiquitin system (the E1, E2, and E3 enzymes have a group of cysteine residues in their catalytic domains that can be modified by ROS), the UPS (irreversible oxidation of UPS subunits (e.g., 20S)), and the autophagy pathway, as will be described later in detail.

In neurons in particular, physiological levels of ROS are important for (i) axonal growth via cytoskeletal regulation [209]; (ii) progenitor cell growth via PI3K/AKT signalling [210]; (iii) neuronal differentiation (a specific redox state is critical for neuronal development) [211]; (iv) synaptic plasticity, via the control of intracellular calcium release and synaptic vesicle release [212]; and (v) a potential role of NOX and NOS proteins regulating long-term potentiation (LTP), pruning, and dendritic growth [213]. In addition, in the brain, ROS generated by glial cells are also involved in the modulation of synaptic activity and other metabolic compartmentalization/crosstalk with neurons (e.g., astrocytes supply essential GSH precursors for neurons [214] and, in hippocampal pyramidal neurons, Atkins et al. described that ROS are involved in a nonsynaptic glial-neuron crosstalk by modifying the myelin basic protein in oligodendrocytes [215]). Most importantly, the presence of these highly reactive species triggers several antioxidant pathways to counter the accumulation of oxidative stress, and maintain cellular homeostasis, as will be described later.

#### 3.2.2. Negative Effects of Free Radicals: Oxidative Stress

ROS/RNS generated inside organelles can be readily released into the cytoplasm. They diffuse across membranes through aquaporins (e.g., aquaporin 8 for H_2_O_2_ release in mitochondria) and other specific unidentified channels [216]. In the cytosol, these highly reactive molecules modify all classes of macromolecules (i.e., carbohydrates, lipids, proteins, and nucleic acids), influence organellar homeostasis, and ultimately induce cell death [217–219]. Consistent with this, it has been recently described that ROS-induced autophagy contributes to ferroptosis, a form of programmed cell death based on iron accumulation [220]. In particular, protein oxidation can cause loss of activity and/or protein unfolding, with the tendency to induce intracellular and extracellular protein oligomers and aggregates that compromise cell viability. Indeed, this is a primary characteristic of neurodegenerative diseases (e.g., *α*-syn in Parkinson's, tau in Alzheimer's, and HTT in Huntington's) [221]. Lipid peroxidation, triggering degradation of cell membrane components, is also induced in response to oxidative stress, as lipids are susceptible to redox modifications; indeed, such changes have been reported in mDANs in Parkinson's brains [222].

The nucleus is highly susceptible to oxidative stress. Diffusion of ROS/RNS into the nucleus influences diverse pathways/components, including chromatin organisation, DNA methylation, histone function (e.g., nitrated or glutathionylated histones), nucleobases, interactions between DNA and DNA-binding proteins, mutagenesis, transcription via targeting of purines and pyrimidines, single- and double-strand breaks, and abasic site formation [223]. Indeed, oxidative stress can be oncogenic by affecting the expression of oncogenes [224], and the formation of DNA adducts can trigger autoimmune disorders [225].

Yoboue et al. proposed a “redox triangle” formed by ER-mitochondria-peroxisome structures, generating a multiorganellar protein complex called the “redoxosome,” where ROS and RNS accumulate to impact organelle function (e.g., ER-mitochondria calcium exchange, oxidative phosphorylation, and protein folding), an idea that awaits mechanistic validation [197, 226]. Indeed, mitochondria-associated membranes (MAM) or mitochondria-ER contacts (MERCs) are modulators of ROS production; calcium crosstalk and autophagosome formation and aberrant MAM structure and function are linked to defective autophagy during neurodegeneration [227–229].

Ultimately, high levels of oxidative stress can induce cell death via apoptosis, necroptosis, and autophagy-associated programmed cell death. Indeed, ROS/RNS activate the extrinsic death receptor pathways (e.g., tumour necrosis factor receptor family), leading to the activation of caspases, as well as the internal mitochondrial and ER cell death pathways. In mitochondria, ROS can induce apoptosis through diverse pathways, including activation of p53 and JNK, which in turn activate proapoptotic Bcl-2 proteins; oxidation of cardiolipin, leading to cytochrome *c* release into the cytosol; ATP depletion; and the induction of mitochondrial membrane depolarization. Low levels of oxidative stress in the ER activate the unfolded protein response (UPR) to inhibit protein translation, and to induce chaperone expression and protein degradation, as will be described later; whereas high levels of ROS trigger the activation of ER stress-mediated apoptosis via different pathways (e.g., prolonged activation of IRE1*α* triggers proapoptotic cascades, upregulation of the proapoptotic transcription factor CHOP, and activation of proapoptotic Bcl-2 proteins in the ER membrane), some of which are interconnected with mitochondrial pathways, leading to caspase activation and apoptosis [207, 230].

Crucially, the brain is particularly susceptible to oxidative stress damage. Cobley et al. defined 13 reasons why the brain is predisposed to oxidative stress and consequent neurodegeneration: (i) redox signalling (high levels of ROS/RNS can induce proapoptotic pathways via redox modifications); (ii) calcium homeostasis (oxidative stress can lead to calcium overload and affect mitochondrial function, leading to programmed cell death); (iii) excessive glutamate uptake (affecting several cellular pathways and producing excitotoxicity); (iv) glucose metabolism necessary to support neuronal activity (oxidative stress can affect this pathway via the formation of advanced end glycation products (AGE)); (v) mitochondria (there is a high ATP demand in neurons, and elevated ROS/RNS levels affect mitochondrial function and ATP formation); (vi) neurotransmitter metabolism (e.g., generation of H_2_O_2_ by monoamine oxidase (MOA), whose activity is disrupted in Parkinson's); (vii) neurotransmitter oxidation (formation of toxic intermediates); (viii) lower antioxidant response in comparison to other tissues; (ix) microglia activation and astrogliosis (as a big source for ROS/RNS); (x) presence of redox active transition metals (e.g., iron and Fenton reaction); (xi) lipid peroxidation (high levels of fatty acids in the brain); (xii) NOS and NOX for neuronal signalling; and (xiii) RNA oxidation [231]. In addition, oxidative stress also impacts the blood-brain barrier permeability, leading to increased trafficking of immune cells and neuroinflammation, another characteristic of neurodegenerative disorders [232].

### 3.3. Antioxidant Pathways for Controlling Redoxtasis

To counterbalance oxidative stress, the cell has developed several antioxidant pathways, including (i) endogenous antioxidant mechanisms (by the presence of molecules and proteins for the removal of free radicals), (ii) a metabolic switch to the pentose phosphate pathway [233], (iii) transcriptional changes by the activation of specific transcription factors, (iv) posttranscriptional regulation via redox-sensitive microRNAs, (v) activation of chaperones and specific degradation systems to avoid protein aggregation, and (vi) the degradation of damaged organelles [234, 235].

Mitochondria are protected from ROS by the presence of antioxidant enzymes that contain cysteine catalytic residues for the reduction of H_2_O_2_ into H_2_O, and by a defence system for the conversion of O_2_^•-^ into the less harmful radical, H_2_O_2_, comprising superoxide dismutase 2 (SOD2) in the mitochondrial matrix, and SOD1 in the intermembrane space. Glutathione peroxidases (GPX1 and GPX4) in the outer mitochondrial membrane, reduce H_2_O_2_ into H_2_O, using reduced glutathione (GSH) as cofactor. Other mitochondrial antioxidant enzymes include the peroxiredoxins (PRX3 and PRX5), which also catalyse the reduction of H_2_O_2_ into H_2_O [197, 236]. Peroxisomes are the other major centre for antioxidant enzyme function. The main ROS defence system here is catalase, which catalyses the reduction of H_2_O_2_ into H_2_O, and indeed deficiencies in this system are linked to cancer and diabetes. In addition, in this organelle, SOD1 and PRX5 are also involved in the formation and reduction of H_2_O_2_, respectively [197, 237]. The ER also houses antioxidant mechanisms, by the presence of GPX7, GPX8, and PRX4 [197]. Other antioxidant molecules in the cell include ascorbic acid, uric acid, melatonin, ubiquinol, and some vitamins, which neutralize free radicals by donating electrons and other regulators of redox signalling, including the electron donor groups thioredoxins (TXN) and glutaredoxins (GRX) [238]. An additional layer of regulated antioxidant response is via cellular metabolic reconfiguration. Here, cellular metabolism is redirected towards the pentose phosphatase pathway, leading to the formation of NADPH which is used by glutathione reductase for GSH reduction, a crucial step in responsive redoxtasis [233, 239].

Transcriptionally, NRF2 is considered to be a master regulator of redoxtasis, controlling around 1% of human genes that share in common the Antioxidant Response Element (ARE) in their promoters [240–243]. Crucially, redox regulation by NRF2 via increasing reduced TXN is also crucial for the modulation of apoptosis signal-regulating kinase 1 (ASK1) activity, involved in ER-stress neuronal cell death [244, 245]. One of the main mechanisms of regulation that cooperate to maintain NRF2 levels within physiological values is KEAP1 (Kelch-like ECH-associated protein 1), a redox-regulated E3 ubiquitin ligase substrate adaptor that promotes NRF2 degradation under basal conditions. High levels of oxidative stress modify KEAP1 to impair its function, leading to increased NRF2 that translocates to the nucleus [246]. This factor regulates the expression of genes involved in redox homeostasis (like Heme Oxigenase-1 (HO-1)) as well as in metabolic detoxification (like NAD(P)H quinone oxidoreductase (NQO1)), inflammation, and proteostasis [247]. In addition, posttranscriptional antioxidant regulation via microRNAs also target this pathway [234].

Finally, the two major quality control mechanisms in the cell have antioxidant roles in preventing the aggregation of oxidized proteins and/or the persistence of damaged organelles (namely, the ubiquitin-proteasome system (UPS) and autophagy [1, 2, 5–7]). Here, we focus on the interplay between autophagy, redox homeostasis, and transcriptional control.

## 4. Autophagy and Redoxtasis Crosstalk

Under stress conditions (e.g., starvation, hypoxia, and uncouplers), ROS/RNS are induced, and have the potential to influence autophagy via core autophagy protein oxidation, or by altering the activities of transcription factors [248–250]. In addition, several lines of evidence suggest that indirect activation of autophagy in response to ROS damage, including DNA oxidation and lipid peroxidation, is crucial for cell survival [251–253]. As ROS damages organelles and biomolecules, their repair and/or removal by fusion/fission or autophagic degradation (e.g., mitophagy; pexophagy; ER-phagy; aggrephagy) is a crucial facet of any ROS response. Thus, a delicate balance is needed between elevated oxidative stress promoting organelle quality control, and its negative effects on components of the autophagy machinery [254, 255] (Figure 1).

### 4.1. Redox Modifications of Autophagy Proteins: Upstream Pathways/Autophagy Induction

The activity of several proteins upstream of the autophagy pathway is affected by ROS/RNS. These proteins are typically also involved in the regulation of several pathways; thus, their redox modifications influence diverse cellular activities.

#### 4.1.1. Receptor Tyrosine Kinases (RTK) for the Activation of PI3K/AKT via Growth Factors (e.g., EGF)

Reversible oxidative and nitrosative modifications include sulfenylation, glutathionylation, disulfide bonds, acylation, and nitrosylation. These affect RTK receptors (including EGFR, FGF, RET, and VEGFR), affecting their activation, localisation, or trafficking, depending on the modification and residues involved (a recent review collecting all known modifications and effects can be found in [256]). In addition, PTP1B activity, involved in the inhibition of RTK signalling, is also affected by oxidation reactions, including sulfenylation, nitrosylation, and glutathionylation [256].

#### 4.1.2. Phosphatase and Tensin Homolog (PTEN)

PTEN opposes PI3K activity by dephosphorylating PIP3 and inhibiting AKT signalling. Redox modifications affect PTEN activity; for example, (i) H_2_O_2_ oxidation inactivates PTEN catalytic activity by the formation of disulfide bonds (C124-C71), leading to autophagy activation in a noncanonical pathway induced my mTOR activation; and (ii) peroxynitrite inhibits PTEN activity and induces neuronal survival and can be oxidized by a lipid peroxide which is prevented by PRX3 [257–259].

#### 4.1.3. Phosphoinositide-Dependent Kinase 1 (PDK1)

PDK1 is a Ser/Thr kinase that activates AKT. Redox modifications of this protein include nitrosylation in different residues leading to inhibition of its kinase activity [260].

#### 4.1.4. AKT

Several cysteines within the pleckstrin homology domain of AKT have been identified as being reversibly oxidised, forming new disulfide bonds. These modifications affect protein function, including the stabilisation of the PI3P pocket, or its inhibition, depending on the modification. In addition, AKT can be inactivated via glutathionylation, which is reversed by glutaredoxin 1 [261, 262].

#### 4.1.5. TSC2

Nitrosylation of TSC2 impairs its dimerization with TSC1, leading to mTOR activation [263].

#### 4.1.6. mTORC1

Generation of disulfide bonds affects mTOR stability and activity depending on the residues involved. Oxidised mTOR can be rescued by Thioredoxin 1 [264]. Related to this, lysosomes can also sense redox signalling specifically via redox-sensing lysosomal ion channels [265].

#### 4.1.7. AMPK

Disulfide bonds, sulfenylation, and glutathionylations have been described to be present in both *α* and *β* AMPK subunits, affecting AMPK activity depending on the modified residues (e.g., disulfide bonds result in AMPK inhibition, and this is reversed by Thioredoxin 1 but other redox modifications result in AMPK activation). In addition, free radicals induce calcium release (e.g., in hypoxia) leading indirectly to the activation of the AMPK via CaMKII activation. Similarly, it has been reported that the induction of autophagy, as a consequence of ROS production in starvation conditions and ATP depletion, is via activation of the AMPK pathway [266–270].

#### 4.1.8. Ataxia Telangiectasia Mutated Protein Kinase (ATM)

ATM is a threonine/serine kinase involved in the DNA damage/repair response. Crucially, ATM is also involved in the induction of pexophagy to maintain redox balance. In response to ROS, ATM activates MAPK, and ATM is transported into peroxisomes via the PEX5 import receptor. Here, it phosphorylates PEX5 triggering ubiquitylation via the E3-like ubiquitin ligase complex, PEX2-PEX10-PEX12, and later recognition by cargo receptors [271, 272]. H_2_O_2_ treatment induces nuclear ATM redox disulfide bond formation, indirectly promoting the downstream expression of proteins involved in the pentose phosphate pathway, and an activator effect via nytrosilation has also been suggested [273–275].

#### 4.1.9. Sirtuin 1 (SIRT1)

A class III histone deacetylase regulating numerous cell activities (e.g., glucose metabolism, chromatin silencing, inflammation, and lipid metabolism) [276], SIRT1 is involved in autophagy via the release of nuclear LC3 during starvation and the deacetylation of ATG5 and ATG7 [277, 278]. In addition, in response to ROS, it is involved in the activation via deacetylation of several autophagy transcription factors, including FOXOs, p53, NRF2, HIF-1*α*, NF-*κ*B, PPARs, and FXR [276, 279]. Conversely, SIRT1 translocation to the nucleus is induced indirectly by the presence of ROS, but SIRT1 can also be modified via oxidation, inhibiting its activity [280–282].

#### 4.1.10. UPR

Numerous redox modifications have been described for the ER stress-sensing proteins, IRE1*α*, PERK, and ATF6 (for a recent review, see [283]). For example, cysteine sulfenylation of cytosolic IRE1*α* blocks UPR activation, but it induces the antioxidant NRF2 pathway [284]. Other modifications include activation of PERK kinase activity via nitrosylation [285], and disulfide bridge formation in ATF6 in unstressed ER [286].

#### 4.1.11. The Cytoskeleton

It is also important to mention that redox modifications can also affect cytoskeletal dynamics [287], and thus, indirectly, autophagy efficiency.

### 4.2. Redox Modifications of Autophagy Proteins: Autophagy Proteins Involved in the Assembly Pathway and Selective Autophagy

Proteins involved in the autophagosome assembly pathway can also be targeted by ROS/RNS. These redox modifications affect the efficiency and productivity of autophagosome biogenesis (Figure 1).

#### 4.2.1. ATG4

ATG8 processing mediated by ATG4 proteins needs to be spatiotemporally regulated to support both autophagy initiation and the availability of a pool of primed ATG8 [288]. Scherz-Shouval et al. found that ROS, produced during starvation, are essential for autophagy via regulation of ATG4. They described a cysteine residue near the catalytic site that is a target for oxidation, thus increasing autophagy initiation by blocking ATG4-mediated delipidation in the vicinity of the expanding autophagosome (exemplified by ATG4A) [289]. Later, Qiao et al. described that ROS induces the formation of a prooxidant complex called REDD1-TXNIP, which inhibits ATG4B function leading to autophagy activation [290]. In addition, Perez-Perez et al. described an inhibitory redox modification in ATG4 in yeast, via the formation of disulfide bonds outside the catalytic site, which can be reversed by Thioredoxin 1 [291]. Finally, nitrosylated ATG4 has been observed in the hippocampal neurons of diabetic rats, and in vitro, in neuronal cells in hyperglycemia conditions leading to neurotoxicity [292].

#### 4.2.2. ATG3 and ATG7

Frudd et al. described that these proteins can be modified by oxidation, including glutathionylation and disulfide bond formation, affecting their function, and leading to autophagy inhibition. Conversely, while inactive, ATG3 and ATG7 form covalent thioester complexes with LC3, preventing their oxidation. However, after autophagy induction, their interactions become more transient, thus increasing susceptibility to redox modifications [293].

#### 4.2.3. BECLIN1

Redox modifications affect BECLIN1 function indirectly: under normal conditions, BECLIN1 forms a complex with proapoptotic BCL-2, which inhibits BECLIN1 activity; however, under autophagy-inducing conditions, BECLIN1 dissociates to establish the PI3KC3 complex. Kitada et al. described that BCL-2 is a target of redox modification, particularly nitrosylation, stabilising the interaction with BECLIN1, and thus preventing autophagy induction [276].

#### 4.2.4. p62/SQSTM1

Carroll et al. identified two cysteine residues in p62/SQSTM1 that can be redox modified, forming disulfide bonds that promote its oligomerisation to enable autophagy induction [294]. They further highlighted the potential effect of p62/SQSTM1 oxidation in aging [294].

#### 4.2.5. Parkin/PKRN

Numerous redox modifications have been described for Parkin, including nitrosylation, sulfonation, and methionine oxidation. Particularly, Chung et al. described that S-nitrosylation reduces Parkin E3 ligase activity, thus affecting its protective function [295], although some discrepancies were reported by a different group [296]. Similarly, Ozawa et al. reported a new site of nitrosylation in Parkin that leads to mitophagy induction via activation of its ligase activity [297]. In addition, Meng et al. described that Parkin can be sulfonated in an in vitro Parkinson's model, leading to protein aggregation, and possibly contributing to the formation of Lewy's bodies in Parkinson's [298]. However, in a recent article (at preprint stage at the point of writing this review), Tokarew et al. highlighted the importance of Parkin's own oxidation in neuroprotection [299]. Previously, Vandiver et al. showed that Parkin can also undergo sulfhydration, enhancing its catalytic activity and its protective function [300]. In addition, they described that in Parkinson's brains, Parkin is highly nitrosylated, but that sulfhydration is reduced [300]. Lee et al. recently reported that Parkin can also undergo methionine oxidation at M192, a residue mutated in early onset Parkinson's [301], and that this is reversed by methionine sulfoxide reductase B2 (MSRB2) released in response to damaged mitochondria, thereby promoting mitophagy [302]. Finally, El Kodsi et al. have reported that Parkin can be glutathionylated in an antioxidant reaction (at preprint stage at the point of writing this review) [303].

#### 4.2.6. PINK1

Oh et al. described that PINK1 can be nitrosylated, inhibiting its kinase activity, and this posttranslational modification is present in Parkinson's mice models where Parkin recruitment is reduced, restricting mitophagy [304].

#### 4.2.7. Protein Deglycase (DJ-1)

DJ-1 overexpression induces mitophagy via the activation of ERK in mDANs, and this protects against rotenone-induced cell death [305]. Indeed, mutations in DJ-1 are linked to familial Parkinson's and some studies suggest that oxidised DJ-1 could potentially be used as a biomarker for Parkinson's [306]. Canet-Aviles et al. observed that cysteine-sulfinic acid formation in DJ-1 is necessary for mitochondrial targeting and neuroprotection [307], and consistent with this, Zhou et al. demonstrated that the sulfinic DJ-1 isoform prevents *α*-synuclein fibrillation [308]. Conversely, in the presence of high levels of oxidative stress, DJ-1 is oxidised to the sulfonic form; this isoform is inactive and predisposed to aggregate formation, and indeed, this overoxidised isoform has been detected in brains from Parkinson's patients [309, 310]. In addition, Ozawa et al. highlighted the crucial role of DJ-1 in the nitrosylation of Parkin, and suggested that DJ-1 inactivation reduces mitophagy, leading to mitochondrial dysfunction and Parkinson's pathogenesis [311].

### 4.3. Redox Modifications of Autophagy Proteins: Autophagy Transcriptional Control

Autophagy gene expression is influenced in different tissues by diverse transcription factors, microRNAs (miRNAs), and by epigenetic modifications [312]. In the last decade, several studies have pointed out that “nuclear” control of autophagy is key for the regulation of the autophagy process, including short-term and long-term outcomes [312]. Currently, numerous transcription factors involved in the regulation of this process have been described. Notable amongst these is transcription factor EB (TFEB), a member of the basic helix-loop-helix leucine-zipper family of transcription factors. TFEB is considered to be a master regulator of autophagy that, under starvation conditions, translocates to the nucleus, where it regulates more than 200 lysosomal-related genes and autophagy genes (including *ATG4*, *ATG9B*, *BECLIN1*, *LC3B*, *GABARAPL1*, *ATG16*, *WIPI*, *UVRAG*, and *p62/SQSTM1*) by binding to CLEAR (coordinated lysosomal expression and regulation network) sequences in their promoters. These genes are involved in autophagosome biogenesis, autophagosome-lysosome fusion, and lysosomal biogenesis [313, 314]. TFEB translocation is regulated primarily by phosphorylation [313, 315], via ERK2 (at S142), and via the autophagy inhibitor mTORC1 (at S211 and S142), to retain TFEB in the cytoplasm by binding to 14-3-3 proteins [314, 316, 317]. Phosphorylated TFEB is also targeted to the proteasome via the E3-like enzyme, STIP1 homology and U-Box-containing protein 1 (STUB1), thereby controlling its stability [318]. Calcineurin, activated by lysosomal calcium release via mucolipin 1 (MCOLN1), binds and dephosphorylates TFEB, causing dissociation from 14-3-3 proteins and translocation to the nucleus [319–321]. Cytoplasmic-nuclear shuttling of TFEB is also observed after refeeding, here modulated via mTORC1 phosphorylation at residues close to the nuclear exported signal (NES) (S142 and S138), with translocation mediated by exportin 1 [322]. An increase in the phosphorylated form of TFEB and dysregulation of autophagy has been correlated with the progression of neurodegenerative diseases, including Parkinson's [323, 324]. In addition, overexpression of TFEB has been reported to be beneficial in numerous disease models via clearance of aggregated protein (e.g., tau in Alzheimer's, *α*-syn in Parkinson's, and HTT in Huntington's [325–327]).

In this review, we focus on transcription factors whose activity is regulated by redox modifications, thus affecting autophagy transcriptional control.

#### 4.3.1. TFEB

In addition to the well-characterised control of TFEB activities via phosphorylation (above), Wang et al. described the regulation of TFEB (and other members of the MiT family) via ROS-mediated cysteine oxidation (C212). This inhibits the interaction of TFEB with Rag GTPases, and induces its nuclear translocation, thus inducing the expression of autophagy/lysosomal genes independently of mTORC1 (although its role in neurodegenerative diseases remains elusive) [328].

#### 4.3.2. The FOXO Family

In particular, FOXO1 and FOXO3 have been identified as autophagy transcription factors, regulating the expression of numerous autophagy-related genes [329–331]. In addition, FOXOs also regulate the expression of antioxidant genes, including *SOD1*, *SOD2*, and *GPX1* [332]. Under basal conditions, FOXOs are phosphorylated by AKT and retained in the cytosol through binding to 14-3-3 [333]. Under stress conditions, they become activated and can either translocate to the nucleus or regulate autophagy in the cytosol—acetylated FOXO1 (under oxidative stress conditions) can bind to ATG7 and activate it [329, 334, 335]. Crucially, it was shown recently that FOXO3a can be degraded by the autophagy pathway, suggesting a negative feedback mechanism for this transcription factor [336]. Mainly, examples of indirect redox regulation of the FOXO family have been described, although direct modifications via formation of disulfide bonds between FOXOs and other proteins have also been reported (e.g., disulfide bridges between FOXO4 and transportin-1 to induce nuclear translocation in response to ROS [337] and disulfide bond heterotrimers between FOXO3, PRX1, and Importin-7/Importin-8, inducing an antioxidant response [338, 339]). Gomez-Puerto et al. reported that FOXO3 is phosphorylated by MAPK and nuclear translocated in response to H_2_O_2_ treatment in human mesenchymal stem cells, thus leading to autophagy induction that is crucial for osteogenic differentiation; however, a direct redox modification has not yet been described [340]. Other examples of indirect redox regulation of the FOXO family include redox modifications of signalling proteins upstream of FOXO, including SIRT1 and AKT, as previously described [332].

#### 4.3.3. NRF2

The master regulator of oxidative stress, NRF2 contributes to the regulation of autophagy-gene expression under these conditions (e.g., *p62/SQSTM1*, *NDP52*, *ULK1*, *ATG2B*, *ATG4*, *ATG5*, and *GABARAPL1*) [341]. Crucially, as mentioned before, the main canonical redox regulator of NRF2 is the NRF2-inhibitor protein KEAP1. Several cysteine residues in KEAP1 can be oxidised leading to conformational changes and thereby preventing NRF2 degradation [342]. In addition, p62/SQSTM1 also binds to KEAP1, marking it for degradation; meanwhile, TFEB represses the NRF2-ubiquitin ligase, DCAF11 (DDB1- and CUL4-associated factor 11)), ultimately promoting NRF2 translocation to the nucleus [343, 344] to establish a feed-forward loop. Finally, there is some evidence indicating that NRF2 is also subject to redox cysteine modifications, promoting NRF2 nuclear translocation; consistent with this, mutations in these cysteines enhance interactions with KEAP1, thus increasing NRF2 degradation [345]. Conversely, recent evidence suggests that NRF2 also regulates CMA via *LAMP2A* expression [346]. In addition, NRF2 directly regulates *HIF-1α* expression, and interacts with ATF4 [347, 348], master regulators of O_2_ homeostasis and contributors to autophagy-gene expression (in mild hypoxia, HIF-1*α* activates the transcription of mitophagy genes (e.g., *NIX*); whereas in severe hypoxia, ATF4 regulates the expression of autophagy genes (e.g., *ULK1* and *LC3B*) [349, 350]). Both, in turn, are regulated by ROS, and act as antioxidant and antiapoptotic proteins [351–354].

#### 4.3.4. P53

As one of the best characterised transcription factors, p53 has been reported to regulate antioxidant genes (e.g., *GPX1*) [355], autophagy genes after DNA damage (e.g., *ULK1*, *ATG4*, *ATG7*, and *ATG10*), and to induce TFEB nuclear translocation [356]. It also stabilises NRF2 indirectly by regulating the expression of p21 and SESN2, prominent KEAP1 interactors [357]. However, cytoplasmic p53 inhibits autophagy via posttranscriptional downregulation of LC3A [358]. Several redox modifications have been reported in p53, including glutathionylation and nitrosylation at residues near the DNA-binding domain, with the former causing inhibition of p53 DNA binding [359]. Indeed, previous data suggest that glutathionylated p53 may be involved in Alzheimer's neurodegeneration [360]; however, nitrosylation seems to be essential for DNA binding and antioxidant gene expression [361].

#### 4.3.5. NF-*κ*B

Under basal conditions, the proinflammatory transcription factor, NF-*κ*B, is inactivated in the cytosol where it interacts with I*κ*B (inhibitor of *κ*B) preventing its nuclear translocation. Previous data suggest that oxidative stress can induce or inhibit the NF-*κ*B pathway, depending on conditions [362]. As one example of indirect regulation, I*κ*B is phosphorylated under oxidative stress, leading to its polyubiquitination and consequent degradation [363]. The consequent elevation of nuclear NF-*κ*B upregulates the expression of several anti-inflammatory and antioxidant genes (e.g., *HO-1*, *Thioredoxin 1*, *GPX1*, *NOS2*, and *SOD2*) [364], and can induce or inhibit autophagy depending on the context (i.e., NF-*κ*B mainly inhibits autophagy, but it can also activate the expression of the autophagy genes including *BECN1* and *p62/SQSTM1* [365–367]). ROS can also directly regulate NF-*κ*B activity. Disulfide bonds in cysteine in the DNA-binding domain inhibits DNA binding, and this can be rescued by Thioredoxin 1 [368]. Similarly, other redox modifications in NF-*κ*B including glutathionylation and nitrosylation also inhibit DNA binding [369, 370].

#### 4.3.6. Other Transcription Factors

Examples of other transcription factors involved in both the regulation of autophagy-related and antioxidant genes include (i) the peroxisome proliferator-activated receptors (PPARs) that upregulate autophagy and can be directly regulated by redox modifications (e.g., nitrosylation) [365, 371, 372] and (ii) the transcription factor, farnesoid X receptor (FXR), mainly expressed in liver and intestine, a nuclear receptor involved in metabolism [373–375]. Under fed conditions in liver, FXR inhibits autophagy-gene regulation directly (e.g., *ULK1*, *ATG2*, *ATG5*, *ATG7*, *WIPI*, *GABARAP*, and *TFEB*) [376].

## 5. Autophagy and Oxidative Stress in Parkinson's

Parkinson's is one of the most common neurodegenerative disorders, second only in prominence to Alzheimer's, and it affects 1-3% of the population aged over 60 [377, 378]. Life expectancy can be lower in many Parkinson's patients [379], due to an increased risk of developing other diseases including infections (pneumonia being the most common cause of death in Parkinson's) [380, 381], certain types of cancers (e.g., brain and breast cancer; although, generally, there is an inverse association between cancer and Parkinson's [382]), and cardiovascular disease [383]. In the UK, the number of Parkinson's patients in 2018 according to the Parkinson's UK website [384] was estimated to be 145,500, and this is predicted to reach 250,000 by 2065. This incidence is affected by age, gender, environmental factors [385], and genetics (10-15% of Parkinson's cases are familial [386], with several recognised Parkinson's-associated genes [387]). In patients, the main symptoms are motor problems including bradykinesia (slowness of movement), hypokinesia (paucity of movements), postural instability (balance impairment), tremor at rest, muscle rigidity, and gait problems (walking abnormalities), with mild cognitive impairments, sleep disorders, and impulsive behaviours also common [388–390].

**Figure 2:**
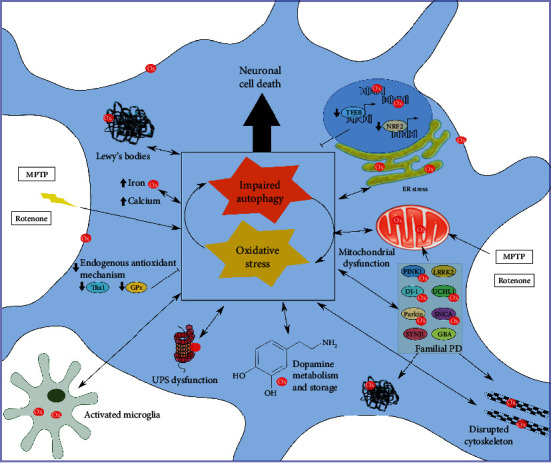
Oxidative stress and autophagy dysregulation in Parkinson's. Oxidative stress and autophagy dysregulation are interconnected in the dopaminergic neurons affected in Parkinson's. In addition, several conditions contribute to this destructive imbalance leading to neuronal death and progressive neurodegeneration, including a reduction in antioxidant pathways (e.g., a reduction in endogenous antioxidant mechanisms and antioxidant transcription factors); ER stress; mitochondrial dysfunction; mutations in key proteins modulating these processes (familial Parkinson's); disruption of the cytoskeleton; UPS dysfunction; neuroinflammation; high levels of calcium and iron, leading to neurotoxicity; neurotoxins (e.g., MPTP and rotenone); and *α*-syn aggregation in Lewy's bodies.

At the cellular level, Parkinson's is characterised by the loss of mDANs, initially in the substantia nigra pars compacta (SNc). As the condition progresses, defects in serotonergic, noradrenergic, cholinergic, GABAergic, and glutamatergic neuronal pathways can also be observed [391]. Although the exact causes of neuronal loss in Parkinson's are not known, the hallmarks that characterise this disease include (i) the accumulation of *α*-syn-rich Lewy's bodies; (ii) increased oxidative stress accompanied by a reduction in antioxidants; (iii) neuroinflammation; (iv) mitochondrial dysfunction; (v) ER stress; (vi) and disruption in protein quality control, including autophagy dysregulation [392]. Crucially, mDANs appear to be particularly sensitive to autophagy deficits, and are frequently exposed to high levels of oxidative stress [96, 231, 325, 393–396]. In the following sections, we will summarise how these two processes are interconnected, and the links with other Parkinson's hallmarks (Figure 2).

### 5.1. Midbrain Dopaminergic Neurons and Degeneration of the Nigrostriatal Pathway

mDANs are localized in the mesencephalon, and are characterised by the production of the catecholaminergic neurotransmitter, dopamine [397]. Dopamine belongs to the monoamine neurotransmitter group that also contains serotonergic or 5-hydroxytryptamine (5-HT) and noradrenergic neurotransmitters [398]. Dopamine biosynthesis occurs by a two-step process in the mDAN cytosol, and is considered the key element of the oxidative stress theory in Parkinson's: (i) tyrosine is hydroxylated to L-DOPA by tyrosine hydroxylase (TH), an enzyme that can also oxidise L-DOPA leading to ROS production [399]; and (ii) L-DOPA is then decarboxylated to dopamine by the aromatic amino acid decarboxylase (AADC), which can be further oxidised as will be described later. Subsequently, dopamine is incorporated into synaptic vesicles via the vesicular monoamine transporter 2 (VMAT2). Inside these vesicles, dopamine is stabilised by the acidic pH. Ultimately, the neurotransmitter is released into the synapse for signal transduction [400]. Dopamine receptors include D1-like receptors (D1 and D5) and D2-like receptors (D2, D3, and D4). [401], and these differ in their localisations within the brain, their modes of action (D1-like receptors activate adenylate cyclase (AC) whereas D2-like receptors inhibit its activation), and their functional influences (e.g., locomotion, emotion, appetite, learning, attention, reward, and memory) [401]. Dopamine reuptake from the extracellular space into presynaptic neurons is regulated by the dopamine transporter (DAT) [402].

There are three different clusters of mDANs, designated as follows: (i) A8, those originating from the retrorubral field (RRF); (ii) A10, those found in the ventral tegmental area (VTA) forming the mesolimbic (to the nucleus accumbens) and mesocortic (to the frontal cortex) dopaminergic pathways; and (iii) A9, the cells located in the SNc that project to the striatum. Functionally, mDANs involved in emotion-based behavior are found in the A8 and A10 groups, whereas those responsible for voluntary movement control are specified as A9 [403]. As A9 SNc mDANs are the first to be lost in Parkinson's, it is worth focusing on the enhanced vulnerability of these cells. In healthy individuals, voluntary movement is controlled in the basal ganglia via a direct (to increase motor activity) and an indirect (to decrease motor activity) pathway that conveys signals to the motor cortex via the thalamus, and from there, on to the spinal cord [404]. In Parkinson's, loss of mDANs correlates with a reduction in dopamine release, leading to under activation of the direct pathway and hyperactivation of the indirect pathway, with an overall increase in thalamus inhibition and reduced voluntary movement being the net effect [405, 406].

Perhaps the most compelling explanation for the vulnerability of mDANs is that their atypical morphology and physiology require that these cells operate close to their energy demand/supply threshold [407]. A9 mDANs have very long, unmyelinated axons, with extensive arborisation, and abundant synapses with unique electrophysiological properties [408–411]. They display autonomous low-frequency pace-making activity, controlled by L-type (Cav1) Ca^2+^ channels, to provide tonic dopamine release to the striatum [407]. This burdens them with the additional challenge of coping with excess cytosolic Ca^2+^. Unfortunately, SNc mDANs have intrinsically low Ca^2+^-buffering capacity, unlike their counterparts in the neighbouring VTA, thus placing an extra reliance on energy-dependent Ca^2+^ efflux and Ca^2+^ sequestration in mitochondria [412–415]. Although high mitochondrial Ca^2+^ supports enhanced oxidative phosphorylation (OXPHOS) rates [416], this comes at the expense of increased mitochondrial oxidative stress [417]. In addition, increased mitochondrial activity results in high levels of cellular iron content due to the numerous mitochondrial enzymes using it as a cofactor. Numerous publications have reported an increased vulnerability of mDANs to iron-induced oxidative stress (e.g., dopamine oxidation), and iron chelators have a neuroprotective effect [418–421]. Indeed, the generation of ROS may result from a disruption of aerobic metabolism. The resulting steady decline in mitochondrial fitness ultimately leads to apoptosis [412, 414], and for this reason, efficient mitochondrial quality control mechanisms are needed to maintain a healthy mitochondrial population in these cells. In addition, their characteristic neuronal morphology, with extensive arborisation and numerous axonal terminals, creates a high energetic demand to sustain their abundant synapses, indicated by the higher density of axonal mitochondria and high dependency on the cellular trafficking machinery [417, 422].

Perhaps most tellingly, neurotoxins that target mitochondria can induce selective mDAN cell death. A neurotoxin used to generate Parkinson's mouse models, 1-methyl 4-phenyl-1,2,3,6-tetrahydropyridine (MPTP), inhibits mitochondrial Complex I in the ETC, causing ROS damage and mitochondrial dysfunction [423]. MPTP can cross the blood-brain barrier, it is metabolised to MPP^+^ and transported by DAT into mDANs [424]. Another Complex I inhibitor is the hydrophobic toxin rotenone, and this can also cross the blood-brain barrier to freely diffuse into cells. Several studies suggest a selective vulnerability of dopaminergic neurons to this compound, and it has been widely used as a Parkinson's-inducing model [425–429]. Rotenone acts by inducing an increase in ROS production, elevating mitochondria bioenergetics, dysregulating intracellular calcium homeostasis, dysregulating autophagy, and altering lipid and glutamine metabolism [417, 430–433]. Other examples of neurotoxins affecting mitochondrial function that are commonly used as Parkinson's models include 6-hydroxydopamine (6-OHDA) and paraquat, causing neurodegeneration via mitochondrial dysfunction and increase in free radicals [424, 434]. In the following sections, we discuss how redox imbalance in the Parkinson's brain affects autophagy pathways to exacerbate the disease.

### 5.2. Autophagy Dysregulation in Parkinson's and Its Interplay with Oxidative Stress

Elevated levels of oxidative stress in Parkinson's correlate with lipid peroxidation, nucleic acid oxidation, elevation of intracellular calcium and increased iron content, and protein oxidation and nitration [395, 435]. In addition, antioxidant deficiencies have been observed in Parkinson's (e.g., low levels of thioredoxin reductase 1 and glutathione peroxidase) [436–438], and might be involved in the aggravation of the disease at early stages (e.g., in early stages of an *α*-syn Parkinson's model, Nrf2 deficiency increased dopaminergic cell death, neuroinflammation, and protein aggregation [439]),while upregulation of antioxidant pathways appear to be beneficial as a potential therapeutic target (e.g., upregulation of the NRF2 pathway prevents neuronal death in MPTP and *α*-syn Parkinson's mice models [440, 441] and restores defective locomotor activity in a *Drosophila* Parkinson's model [442]). On the other hand, increased immunoreactivity of ROS-producing enzymes, particularly NOX complexes, has also been observed in Parkinson's [443], and indeed, NOX2 activation in a rotenone-induced Parkinson's model impairs autophagy and induces cell death [444], while inactivation of NOX complexes has a neuroprotective effect [445].

One of the main features of oxidative stress in mDANs is the oxidation of dopamine. In dopaminergic neurons, cytosolic excess dopamine is oxidised to the metabolite aminochrome or other toxic dopamine quinones. These are intermediates in the normal process of dopamine oxidation to neuromelanin, a dark polymer pigment that accumulates with age in the SNc and has neuroprotective roles as a metal chelator (e.g., preventing iron-mediated oxidative damage) [446, 447]. Aminochrome is toxic, and has been proposed to play an important role in the neurodegenerative process through different mechanisms: (i) the formation and stabilisation of neurotoxic protofibrils of *α*-syn aggregates [448]; (ii) mitochondrial dysfunction by inhibiting Complex I [449]; (iii) cytoskeletal disruption and impairment of axonal transport, with restricted autophagosome-lysosome fusion and lysosomal dysfunction [450, 451]; and (iv) neuroinflammation via the activation of microglia and astrocytes [452]. Consequently, aminochrome contributes to autophagy impairment both cell autonomously [453], and noncell autonomously via neuroinflammation, which itself is linked to glial autophagy dysfunction (e.g., the inflammatory cytokine, TNF*α*, impairs autophagy flux in microglia via mTORC1 [454]).

Over the previous decade, numerous lines of evidence have highlighted the importance of autophagy in neuronal homeostasis. In the absence of autophagy in dopaminergic neurons (in *Atg7* knockout mice), inclusions containing *α*-syn and p62/SQSTM1 are observed predominantly in neurites, and these increase with age, preempting neurodegeneration and motor dysfunction [96]. Particularly, the location of autophagic structures and cargo needs to be considered due to the unique characteristics of autophagosome assembly, maturation, and trafficking in neurons (e.g., fewer autophagosomes in the soma might be an indication of disrupted retrograde transport from the distal axon, including oxidative modifications that can affect the cytoskeleton) [287, 455–457]. This implicates autophagy in mDAN protection, and indeed, genetic autophagy induction (*BECN1*, *TFEB*, and *LAMP2A* overexpression) in *α*-syn (*SNCA*) overexpression mouse models ameliorates synaptic and dendritic pathology [325, 458, 459], while induction via rapamycin (mTORC1 inhibitor) treatment in induced pluripotent stem cell- (iPSC-) derived neurons promotes clearance of *α*-syn aggregates and reduces oxidative stress levels in a paraquat-induced Parkinson's mice model [105, 460]. Conversely, Hunn et al. in 2019 showed that impaired macroautophagy (*Atg7* conditional knockout) in a *SNCA* mouse model led to dopamine release and improved motor movement, while aggravating pathology as reported by increased p62/SQSTM1 inclusions and neuronal death [461]. In addition, it is known that inefficient mitophagy plays an important role in the pathogenesis of Parkinson's, including an accumulation of mitochondrial ROS [462], an observation that correlates with the high vulnerability of SNc mDANs with high energetic demands. Mutations in *PINK1*, *PRKN/Parkin*, and *FBXO7*—three proteins involved in the recognition of damaged mitochondria—are linked to familial Parkinson's (identified as *PARK2*, *PARK6*, and *PARK15*, respectively) [463]. In addition, as previously described, redox modifications have been described for PINK1 and Parkin, highlighting the interplay between these two pathways. Indeed, in hiPSC-based Parkinson's disease models, PINK1 nitrosylation—also observed in transgenic Parkinson's mice models—correlates with reduced Parkin recruitment efficiency and mitophagy disruption [304]. Similarly, oxidised forms of Parkin have been described in Parkinson's, including Parkin sulfonation linked to protein aggregation (including Lewy's body formation and associated redox changes) in an in vitro MPTP-induced Parkinson's model (for a description of all oxidised Parkin forms, see Section 4.2) [295, 298, 300]. In addition, in zebrafish, loss of *Nipsnap1*—a mitochondrial matrix protein involved in PINK1/Parkin-independent mitophagy and highly expressed in mDANs—caused Parkinson's hallmarks [9, 135]. In the light of these findings, an autophagy inducer, Nilotinib, is currently in clinical trial for Parkinson's [464, 465].

Apart from the mutations found in mitophagy genes, several genes linked to familial Parkinson's either directly or indirectly modulate autophagy (including *UCHL1*, *DJ-1*, *LRRK2*, *ATP13A2*, *USP24*, *HTRA2*, *VPS35*, *SYNJ1*, *VPS13C*, and *GBA*) [387, 466], and some of them that are also linked to and/or regulated by ROS/RNS are discussed below.

#### 5.2.1. UCHL1 (Ubiquitin C-Terminal Hydrolase L1, PARK5)

UCHL1 is a deubiquitylating enzyme [467]. It can impair autophagosome formation, with the *UCHL1* I93M mutant overriding this suppression [468]. In response to oxidative stress, UCHL1 promotes cell survival in cancer cells; however, in rotenone-induced Parkinson's mouse models, Kumar et al. reported that UCHL1 undergoes nitrosylation, disrupting its deubiquitinase activity and causing structural instability and aggregation, thereby promoting *α*-syn aggregation [469, 470].

#### 5.2.2. DJ-1 (Protein Deglycase, PARK7)

DJ-1 acts in parallel with the PINK1/Parkin pathway, playing an important antioxidant role to protect mDANs against oxidative damage [471]. However, its roles are widespread in the cell (for a recent review, see [472]). DJ-1 is a cytosolic protein, but under stress conditions (e.g., oxidative stress), it translocates to mitochondria and to the nucleus. It contributes to (i) dopamine production via activation of TH and ADCC; (ii) regulation of mitochondrial activity via interactions with the antiapoptotic protein BCL-xL and Complex I; (iii) the upregulation of CMA, acting as a molecular chaperone interacting with *α*-syn; and (iv) the regulation of transcriptional activity via activation of NF-*κ*B, p53, and NRF2 pathways [308, 472]. Crucially, through NF-*κ*B regulation, DJ-1 controls the expression of mitochondrial uncoupling proteins, UCP4 and UCP5, that decrease mitochondrial membrane potential, thereby suppressing ROS production; meanwhile, DJ-1 binds to Complex I via NDUFA4 to maintain its activity, and is thus crucial for mDAN survival [472]. As previously described, DJ-1 is also involved in the nitrosylation of Parkin1 [311], and DJ-1 itself can be oxidised. This is crucial for neuroprotection, and indeed, levels of oxidised DJ-1 are reduced in Parkinson's patients [307, 473], and low levels of DJ-1 increase vulnerability to oxidative stress [474].

#### 5.2.3. LRRK2 (Leucine-Rich Repeat Kinase 2, PARK8)

LRRK2 is degraded by CMA, and its most common mutation (G2019S) increases its kinase activity, restricting its degradation [475–477]. G2019S LRRK2 can also inhibit CMA activity, affecting CMA substrate degradation in general [475, 478]. LRRK2 comprises multiple domains, and thus regulates several, distinct functions, including neurite outgrowth, vesicle trafficking, nuclear organisation, mitochondrial homeostasis, and autophagy, via different pathways [479]: (i) it activates endophilin A, a neuron-specific protein involved in recruitment of ATG3 [118]; (ii) it modulates mitophagy via Rab10 and Parkin interactions [480, 481]; (iii) it regulates autophagy by activating ERK, MAPK, and PI3KC3-C1 [482]; and (iv) it modulates lysosomal pH via interactions with the proton pump [483]. Although yet to be confirmed in mammalian cells, yeast wild-type LRRK2 appears able to protect against oxidative stress, depending on mitochondrial function and endocytosis, and an increase in dopamine oxidation has been reported in mutated LRRK2 neurons [484, 485]. Indeed, LRRK2 function may be regulated by ROS, as arsenite and H_2_O_2_ treatments downregulate LRRK2 phosphorylation, preventing binding to 14-3-3 in vitro [486, 487].

Mutations in the lysosomal enzyme, glucocerebrosidase (*GBA*)—in which homozygous mutations lead to Gaucher's disease—are one of the most common risk factors for Parkinson's, and GBA deficiency is associated with mitochondrial dysfunction and oxidative stress [488, 489]. In addition, Li et al. reported in iPSC-derived mDANs from Parkinson's patients with mutations in GBA, autophagic and lysosomal defects, with impaired calcium homeostasis and mitochondrial dysfunction in mouse and neuroblastoma cells and increase in oxidative stress [490].

Finally, as we previously mentioned, glial cells—i.e., astrocytes, microglia, and oligodendrocytes—represent about 90% of all cells in the brain, and are critical for maintaining neuronal homeostasis (e.g., synapse functions, metabolism, neurodevelopment, myelination, neuroinflammation, and axonal regeneration) and alterations of neuron-glia signalling pathways are associated with neurodegenerative diseases, including Parkinson's [491–493]. A potential intercellular regulation of neuronal autophagy by glial cells has been reported in induced pluripotent stem cell- (iPSC-) derived motor neurons using conditioned media from iPSC-derived astrocytes from ALS patients [494]. In addition, overexpression in astrocytes of the oxidative stress regulator and autophagy transcription factor NRF2 promotes *α*-synuclein degradation in an *α*-synuclein mutant (A53T) mouse model [341, 495]. Similarly, di Domenico et al. showed that iPSC-derived astrocytes derived from Parkinson's patients presented deficient CMA, impaired macroautophagy, and *α*-syn aggregates, and this was rescued with a CMA activator [496]. However, the exact mechanism for the regulation of neuronal autophagy by this pathway remains elusive. On the other hand, recent studies support a model of direct transfer of cellular garbage from neurons to glial cells for their degradation, especially relevant for mitochondria, in a process termed “transmitophagy” [497]. In this study, damaged mitochondria in the axons of retinal ganglion cells were engulfed and degraded by neighbouring astrocytes [497]. In addition, in *C. elegans*, neurons release vesicles called exophers in a process that is enhanced during stress or when autophagy is inhibited, and these contain protein aggregates and organelles that are subsequently engulfed by adjacent cells [498].

### 5.3. Parkinson's Hallmarks Linking Autophagy Disruption with Increased Oxidative Stress

Certain hallmarks of Parkinson's are thought to exacerbate pathology through the disruption of autophagic flux. Here, we will describe how different Parkinson's features are linked to autophagy disruption and oxidative stress damage.

#### 5.3.1. Lewy's Bodies

The best characterised feature of Parkinson's is the presence of Lewy's bodies; however, these are not observed in all Parkinson's cases, and are also found in healthy patients where they are referred as incidental LB disease [499]. *α*-Syn is a major component of this fibrillar aggregate; however, more than 70 additional molecules have been identified as coconstituents (including DJ-1, PINK1, Parkin (sulfonylated Parkin leads to protein aggregation and contributes to Lewy's body formation [298]), and LRRK2) [500]. Mutations in *SNCA* (e.g., A53T and A30P), posttranslational modifications (e.g., phosphorylation, ubiquitination, and oxidation), and autophagy dysfunction increase the rate of oligomerisation, and thus the formation of inclusions [501, 502]. Importantly, several redox modifications have been described for *α*-syn. For example, Giasson et al. reported that *α*-syn can be nitrated at specific tyrosine residues, and these modifications are found in Lewy's bodies [503]. Jiang and Chang demonstrated the presence of disulfide bonds in *α*-syn that enhance its propensity to aggregate [504]. Ponzini et al. described that *α*-syn methionine oxidation inhibits secondary structure formation [505]. In addition, mitochondria-associated ER membranes—whose functions are compromised in Parkinson's—are also the residence of a subpopulation of *α*-syn, and mutations or overexpression of *α*-syn enhances the extent of contact sites and affect mitochondrial function [506–509].

Overall, accumulation of *α*-syn in oligomers affects neurotransmitter release, synaptic vesicle recycling and trafficking, and autophagy (both CMA and macroautophagy); meanwhile, it increases ROS/RNS levels (e.g., *α*-syn oligomers induce Parkin, DJ-1, and UCHL1 nitrosylation), triggers microglial activation, impacts on mitochondrial homeostasis, and induces ER stress and calcium homeostatic imbalance [501, 510–518]. In its aggregated form, *α*-syn can block CMA, thereby preventing the degradation of itself and other CMA substrates [513]. Aggregates of *α*-syn also stimulate cell death via oxidative-nitrosative stress, and this [507]further enhances *α*-syn persistence leading to a compensatory response of increased macroautophagy and an accumulation of aggregates in autophagosomes [2]. Conversely, *α*-syn aggregates can also impair macroautophagy (e.g., the A30P *SNCA* mutant impairs macroautophagy via inactivation of c-Jun and activation of the transcriptional repressor, ZKSCAN3 [515]) [519, 520]. In addition, several studies have highlighted the presence of different mechanisms for toxic *α*-syn aggregates to be transferred to other cells, including via exosomes, direct penetration, endocytosis, nanotubes, *trans*-synaptic junctions, or receptor-mediated internalisation, all of which are predicted to spread pathology within the brain [510, 521], and indeed, exocytosis and prion-like intercellular transfer of *α*-syn increase with oxidative stress and autophagy impairment [522, 523].

#### 5.3.2. Neuroinflammation

Another Parkinson's hallmark that may impact on or be affected by redox imbalance is neuroinflammation [524]. Neuroinflammation is an immune response mainly controlled by microglia and astrocytes in order to respond to an injury, and remove cell debris, and triggered in response to toxic molecules. Microglia and astrocytes act as antioxidant systems to remove excess ROS/RNS; however, in Parkinson's, free radical levels exceed the detoxifying capacity, which can be also compromised due to genetic mutations and autophagy disruption. Oxidative stress exacerbates this chronic response due to the release of oxidised molecules, including neuromelanin, aminochrone, and *α*-syn [525]. In turn, chronic neuroinflammation is thought to increase oxidative stress by inducing reactive astrogliosis and microgliosis, leading to the production of ROS/RNS, and contributing to mDAN death [526, 527]. In addition, neuroinflammation is also closely related to autophagy dysfunction. Indeed, TNF*α* impairs autophagy flux in microglia, and autophagy induction promotes microglia polarisation towards a M2 neuroprotective phenotype [454]. Similarly, neuroinflammation in premotor neurons in stress-induced hypertension rats blocks autophagy flux [528], and mitochondrial antiviral signaling (MAVS) in microglia is involved in microglial activation and this is negatively regulated by autophagy [529]. In addition, autophagy regulates the inflammasome—an innate immune system complex—which in turn inhibits autophagy, and directly regulates IL-1*β* signalling [530–532]. Indeed, autophagy-deficient microglia lead to an increase in inflammasome activation and causes Parkinson's-like symptoms in mice [533]. Similarly, autophagy inhibition contributes to the exacerbated proinflammatory response in microglia, while autophagy dysfunction in astrocytes might contribute to the progression of the disease; these are reviewed in detail elsewhere [534, 535].

#### 5.3.3. Impairment of the UPS

Disrupted protein quality control in Parkinson's is linked to impairment of the UPS, involved in the selective degradation of the majority of abnormal proteins in the cell [536]. Oxidised and damaged proteins are mainly degraded by the proteasome and the autophagy pathway. *α*-Syn aggregates, mitochondrial dysfunction, oxidative stress, familial Parkinson's (e.g., Parkin and UCHL1 mutations correlate with reduction in proteasome function), and other conditions impair UPS function, which in turn, leads to an increase in oxidised and damaged protein levels, increased toxic iron, and increasing vulnerability of mDANs [537–539]. Indeed, in an UPS-impaired Parkinson's mouse model, UPS inhibition activated the autophagy-lysosomal system in mDANs [540]. UPS and autophagy are closely related and dynamically interconnected, where p62/SQSTM1 appears to be one of the main modulators [541–543]. In addition, previous evidence indicates that proteasome function is modulated by redox modifications [544].

#### 5.3.4. ER Stress

ER stress/dysfunction and chronic UPR activation are further Parkinson's hallmarks [545]. Disruptions in the protein quality control systems and increase in oxidative stress levels contribute to an increase in misfolded proteins in the ER, leading to an exacerbated ER stress and a chronic UPR activation. When ER stress is too severe, it contributes to the generation of oxidative stress and the UPR initiates programmed cell death [546]. Consistent with this, numerous lines of evidence highlight a specific vulnerability of mDANs to ER stress and protein misfolding [518]. For example, CHOP depletion in mice has a neuroprotective role in mDANs against 6-OHDA, but not in a MPTP Parkinson's model [547]. Similarly, mDANs deficient of the UPR transcription factor X-box binding protein 1 (XBP1) were resistant to 6-OHDA; however, XBP1 downregulation in the SNc caused increased neurodegeneration linked to ER stress, and local SNc XBP1 overexpression had a neuroprotective effect against 6-OHDA or MPTP [548, 549]. Indeed, similar results were found with ATF6 overexpression protecting against MPTP neurodegeneration [550]. MPTP treatment also affects calcium homeostasis in the ER via inhibition of the store-operated calcium entry (SOCE) leading to calcium imbalance [551]. In addition, ER-mitochondria associations—required for calcium homeostasis, mitochondrial function, autophagy, and ER functionality [227]—are altered in Parkinson's, as is the mitochondrial UPR [507, 552]. Overall, the crucial role of ER stress in Parkinson's pathology suggest a key role of ER-phagy; however, the influence of ER-phagy in Parkinson's initiation and progress remains elusive.

#### 5.3.5. Peroxisomal Dysfunction: A New Player?

Finally, it is also important to mention that the other major source of ROS/RNS in the cell, peroxisomes, are also affected in Parkinson's. Indeed, peroxisomes are required for neuronal homeostasis and function, and peroxisomal dysfunction has been suggested to contribute to *α*-syn aggregation [553–555]. In addition, recently, Jo et al. identified the mitochondrial chaperone HSAP9—associated with Parkinson's [556]—acting as a pexophagy regulator in vitro and in vivo; HSAP9 downregulation in neuroblastoma cells increased pexophagy activity, and this could not be rescued by HSAP9 mutated forms found in Parkinson's patients [557]. However, the precise roles of peroxisomes and pexophagy in Parkinson's pathology remain unclear.

Overall, autophagy dysfunction and increased oxidative stress are two closely related hallmarks present in dopaminergic neurons in Parkinson's, aggravated by other Parkinson's features including mitochondrial dysfunction, elevated iron and calcium levels, increase in dopamine oxidation, UPS dysfunction, ER stress, neuroinflammation, and *α*-syn aggregation.

## 6. Overview and Conclusions

Autophagy is an intracellular process required for the maintenance of cellular homeostasis, being particularly crucial in neurons as they are postmitotic cells highly vulnerable to stress. Dysfunctional autophagy typically correlates with neurodegenerative diseases, with mitophagy being a particularly important link due to the increased vulnerability of mDANs to autophagy deficits and mitochondrial dysfunction [412, 417]. The regulation of autophagy is closely related to redox homeostasis. To maintain homeostasis, cells have developed antioxidant mechanisms to control the level of free radicals in the cell, including the turnover of ROS-damaged organelles. Upon stress or injury, autophagy is one of the main antioxidant pathways in the cell via the degradation of damaged organelles (e.g., degradation of the major source of free radicals in the cell including mitophagy, ER-phagy, and pexophagy) as well as damaged/misfolded proteins. Basal ROS/RNS levels are also important in the cell as they are involved in cellular signalling, highlighting the beneficial effect of these radicals for cell survival. Indeed, oxidative modifications in redox-sensitive amino acids in proteins involved in the autophagy pathway have been described, including (i) those involved in the upstream pathway; (ii) those directly involved in the process, and iii) those involved with transcriptional regulation of autophagy, highlighting the interplay between these two processes.

However, when the balance of antioxidant mechanisms and ROS/RNS generation is disrupted because the antioxidant defence is reduced (e.g., dysfunctional autophagy), or because ROS generation is increased, oxidative stress is initiated. This can damage the cell, including triggering neuronal cell death programmes, the primary driver in Parkinson's. In addition, dopaminergic neurons are particularly vulnerable to autophagy deficits and high levels of oxidative stress. Consistent with this, different factors contribute to the disruption of the autophagy pathway linked to an increase in oxidative stress, including (i) dopamine oxidation leading to the formation of the toxic molecules like aminochromone; (ii) familial Parkinson's-associated genes involved in autophagy and oxidative stress regulation; (iii) mitochondrial dysfunction, including an increase in calcium and iron levels; (iv) neurotoxins affecting almost exclusively dopaminergic neurons (e.g., MPTP, rotenone); (v) oxidation of biomolecules (e.g., lipid peroxidation, DNA oxidation, and *α*-synuclein oxidation); (vi) UPS dysfunction; (vii) disruption in the cytoskeletal transport; (viii) neuroinflammation; (ix) ER stress and chronic activation of the UPR; and possibly (x) peroxisomal dysfunction.

Currently, there are only symptomatic treatments for Parkinson's, and no disease-modifying therapies have been described. The most commonly used approaches to treat motor deficiencies are based on pharmacological stimulation of the dopaminergic pathway—e.g., levodopa (L-DOPA, dopamine precursor), dopamine agonists [558], and nonpharmacological treatments such as deep brain stimulation (DBS) [559]. However, none of these are capable of delaying or stopping the progression of the disease. Other promising therapies, some of them yet not tested in humans, have been developed over recent years, including stem cell-based approaches (stem cell and induced pluripotent stem cells derived from patients' fibroblasts have emerged as a powerful tool to obtain a renewable source of dopaminergic neurons that can integrate in the brain [560, 561]), the use of neurotrophic factors (e.g., BDNF and GDNF (glial-derived neurotrophic factor)), antioxidants as neuroprotective compounds (e.g., NOS inhibitors, iron chelators, and NRF2 activators [438, 441, 562, 563]), gene therapy (e.g., viral-gene expression of TH, AADC, or VMAT2 to induce dopamine release, or NURR1 expression which appears to have a neuroprotective role), and immunotherapy (e.g., for the clearance of *α*-synuclein aggregates) [564–567]. In addition, another divergent approach is enhancing the autophagy process. The most tested autophagy enhancers are the mTORC1 inhibitor, rapamycin, and the inositol monophosphatase inhibitor, lithium, each of which significantly reduced *α*-synuclein aggregates and cell death in Parkinson's models [568, 569]. However, they are nonselective for autophagy, affecting other pathways, and thus treatment with these compounds presented numerous side effects [570]. For this reason, recent strategies have focused on specific targeting of autophagy components (e.g., TFEB and Beclin1) [324, 325, 458, 571–573], or the lysosome, including increased acidification and overexpression of LAMP2A (targeting CMA) [459, 571, 574–576].

Overall, there is a strong evidence for the interplay of autophagy and redox homeostasis and how it plays a crucial role in Parkinson's. However, there is still a lot to explore and future research would contribute to a better understanding of this tight relationship and potential target for selective therapies.

## Figures and Tables

**Table 1:** Key proteins involved in the various stages of autophagy and their general roles.

Protein	Functions	Stages
*ATG proteins*		
ULK1/2	Serine/threonine kinase that forms complexes with ATG13, FIP200, and ATG101, involved in ATG9 recruitment and the activation of the PI3KC3 complex.	Initiation/nucleation
ATG2A/B	ATG2A interacts with WIPI4, tethering the omegasome to the ER. ATG2-GABARAP interaction is critical for autophagosome closure.	Elongation and maturation (closure)
ATG3	E2-like enzyme coordinating ATG8 conjugation to PE.	Elongation
ATG4A/B/C/D	Cysteine protease that activates (priming) and recycles (delipidation) ATG8s by cleavage of pro-ATG8 and ATG8-PE, respectively for autophagosome formation and possibly maturation).	Elongation
ATG5	Conjugates to ATG12, and acts as an E3-like enzyme for ATG8 conjugation to PE.	Elongation
BECLIN1	Regulatory subunit of the PI3KC3 Complex I.	Initiation/nucleation
ATG7	E1-like enzyme. Coordinates conjugation of ATG12 to ATG5, and ATG8 conjugation to PE.	Elongation
LC3A/B/C	Conjugates to the lipid, PE. Involved in membrane tethering, and phagophore expansion and closure. Coordinates cargo recruitment by binding to autophagy receptors. Binding to FYCO1 promotes microtubule-based transport and autophagosome maturation. Regulates autophagosome-lysosome fusion (binding to PLEKHM1 and HOPS).	Elongation, maturation, and fusion
GABARAPs	Parallel functions with LC3A/BC, although GABARAPs seem to be particularly important for autophagosome maturation.	Elongation, maturation, and fusion
ATG9	Transmembrane protein involved in delivery of membrane to the PAS/autophagosome assembly site.	Initiation/nucleation
ATG10	E2-like enzyme. Coordinates the conjugation of ATG12 to ATG5.	Elongation
ATG12	Conjugates to ATG5, forming the E3-like enzyme for conjugation of ATG8 to PE.	Elongation
ATG13	Regulatory subunit of the ULK1/2 complex.	Initiation/nucleation
ATG14L1	Core component of PI3KC3 Complex I, required for ER localization. Stabilises SNARE complexes for autophagosome-lysosome fusion.	Initiation/nucleation and fusion
ATG16L1	Forms a complex with ATG12-ATG5. Provides E3-like activity for conjugation of ATG8 to PE.	Elongation
ATG17	Scaffolding protein for the recruitment of ATG9 vesicles. ESCRT recruitment.	Initiation/nucleation and maturation (closure)
ATG101	Core component of the ULK1 complex.	Initiation/nucleation
FIP200	Core component of the ULK1 complex.	Initiation/nucleation
WIPI1/2/3/4	PI3P effector protein. WIPI2 recruits the ATG12-ATG5-ATG16L1 complex at the phagophore.	Elongation
*Non-ATG proteins*		
ALFY	PI3P effector protein involved in the degradation of protein aggregates.	Elongation
AMBRA1	Regulator of the PI3KC3 complex.	Initiation/nucleation
AMPK	Serine/threonine kinase. Autophagy activator via phosphorylation of ULK1 and inhibition of mTOR.	Initiation/nucleation
Basson	Scaffold protein in neuronal active zone. Involved in ATG5 sequestration.	Initiation
DFCP1	PI3P effector protein. Efficient omegasome marker.	Elongation
Endophilin A	Adaptor protein involved in synaptic vesicle recycling and ATG3 recruitment.	Initiation
ESCRT	Membrane fission.	Maturation (closure)
FBXO7	E3-like enzyme involved in mitochondrial Parkin recruitment.	Initiation/nucleation (mitophagy)
FYCO1	Rab7 effector. Binds to PI3P and LC3. Mediates anterograde kinesin-driven transport.	Maturation (trafficking)
mTORC1	Serine/threonine kinase complex. Autophagy inhibitor via phosphorylation of ULK1.	Initiation/nucleation
Parkin	E3-like enzyme. Ubiquitination of mitochondrial surface proteins.	Initiation/nucleation (mitophagy)
PEX5	Protein family involved in peroxisome biogenesis and pexophagy. PEX5 is ubiquitinated by the PEX2-PEX10-PEX12 E3-like complex and it is recognised by cargo receptors.	Initiation (pexophagy)
Piccolo	Scaffold protein in the neuronal active zone. Involved in ATG5 sequestration.	Initiation
PINK1	Serine/threonine kinase. Drives the phosphorylation of ubiquitin and Parkin, for robust mitochondrial Parkin recruitment.	Initiation/nucleation (mitophagy)
RAB7	Autophagosome trafficking (interaction with FYCO1 or RILP) and autophagosome-lysosome fusion (interaction with PLEKHM1).	Maturation and fusion
Synaptojanin	Enzyme involved in neuronal membrane trafficking. Promotes autophagosome maturation.	Maturation
SNAREs	On the autophagosome, STX17 and SNAP29, and on the lysosome, VAMP7 or VAMP8, mediates membrane fusion supported by HOPS and ATG14L1.	Fusion
TBK1	Serine/threonine kinase. Increases the binding affinity of autophagy receptors	Elongation
UVRAG	Core component of PI3KC3 Complex II.	Maturation and fusion
VPS15	Adaptor protein and core component of PI3KC3 complex.	Initiation/nucleation
VPS34	Catalytic subunit of the PI3KC3 complex.	Initiation/nucleation
*Autophagy receptors*		
P62, NDP52, OPTN, NRB1, TAX1BP1, NIX, FUNDC1, CCPG1, RTN3, SEC62, ATL3, CALCOCO1, FAM134B, TEX264	Binding to ubiquitinated substrates and ATG8s.	Cargo recruitment to the phagophore

## References

[1] Wang C., Wang X. (2015). The interplay between autophagy and the ubiquitin-proteasome system in cardiac proteotoxicity. *Biochimica et Biophysica Acta (BBA)-Molecular Basis of Disease*.

[2] Pajares M., Jiménez-Moreno N., Dias I. H. K. (2015). Redox control of protein degradation. *Redox Biology*.

[3] Zheng Q., Huang T., Zhang L. (2016). Dysregulation of ubiquitin-proteasome system in neurodegenerative diseases. *Frontiers in Aging Neuroscience*.

[4] Schneider J. L., Cuervo A. M. (2014). Autophagy and human disease: emerging themes. *Current Opinion in Genetics & Development*.

[5] Kleiger G., Mayor T. (2014). Perilous journey: a tour of the ubiquitin-proteasome system. *Trends in Cell Biology*.

[6] Dikic I. (2017). Proteasomal and autophagic degradation systems. *Annual Review of Biochemistry*.

[7] Dikic I., Elazar Z. (2018). Mechanism and medical implications of mammalian autophagy. *Nature Reviews Molecular Cell Biology*.

[8] Cuervo A. M. (2008). Autophagy and aging: keeping that old broom working. *Trends in Genetics*.

[9] Gao F., Yang J., Wang D. (2017). Mitophagy in Parkinson’s disease: pathogenic and therapeutic implications. *Frontiers in Neurology*.

[10] Wong Y. C., Holzbaur E. L. F. (2015). Autophagosome dynamics in neurodegeneration at a glance. *Journal of Cell Science*.

[11] Tandon M., Othman A. H., Ashok V., Stein G. S., Pratap J. (2018). The role of Runx2 in facilitating autophagy in metastatic breast cancer cells. *Journal of Cellular Physiology*.

[12] Ramesh J., Ronsard L., Gao A., Venugopal B. (2019). Autophagy intertwines with different diseases—recent strategies for therapeutic approaches. *Diseases*.

[13] Thorburn A. (2018). Autophagy and disease. *The Journal of Biological Chemistry*.

[14] Yin X., Zhou C., Li J. (2019). Autophagy in bone homeostasis and the onset of osteoporosis. *Bone Research*.

[15] Oku M., Sakai Y. (2018). Three distinct types of microautophagy based on membrane dynamics and molecular machineries. *BioEssays*.

[16] Bonhoure A., Vallentin A., Martin M. (2017). Acetylation of translationally controlled tumor protein promotes its degradation through chaperone-mediated autophagy. *European Journal of Cell Biology*.

[17] Lv L., Li D., Zhao D. (2011). Acetylation targets the M2 isoform of pyruvate kinase for degradation through chaperone-mediated autophagy and promotes tumor growth. *Molecular Cell*.

[18] Kaushik S., Cuervo A. M. (2018). The coming of age of chaperone-mediated autophagy. *Nature Reviews Molecular Cell Biology*.

[19] Caballero B., Wang Y., Diaz A. (2018). Interplay of pathogenic forms of human tau with different autophagic pathways. *Aging Cell*.

[20] Glick D., Barth S., Macleod K. F. (2010). Autophagy: cellular and molecular mechanisms. *The Journal of Pathology*.

[21] Feng Y., He D., Yao Z., Klionsky D. J. (2014). The machinery of macroautophagy. *Cell Research*.

[22] Ktistakis N. T., Tooze S. A. (2016). Digesting the expanding mechanisms of autophagy. *Trends in Cell Biology*.

[23] Lamb C. A., Yoshimori T., Tooze S. A. (2013). The autophagosome: origins unknown, biogenesis complex. *Nature Reviews Molecular Cell Biology*.

[24] Takeshige K., Baba M., Tsuboi S., Noda T., Ohsumi Y. (1992). Autophagy in yeast demonstrated with proteinase-deficient mutants and conditions for its induction. *The Journal of Cell Biology*.

[25] Tsukada M., Ohsumi Y. (1993). Isolation and characterization of autophagy-defective mutants of *Saccharomyces cerevisiae*. *FEBS Letters*.

[26] Harnett M. M., Pineda M. A., Latré de Laté P. (2017). From Christian de Duve to Yoshinori Ohsumi: more to autophagy than just dining at home. *Biomedical Journal*.

[27] Filomeni G., De Zio D., Cecconi F. (2015). Oxidative stress and autophagy: the clash between damage and metabolic needs. *Cell Death and Differentiation*.

[28] Gatica D., Lahiri V., Klionsky D. J. (2018). Cargo recognition and degradation by selective autophagy. *Nature Cell Biology*.

[29] Shaid S., Brandts C. H., Serve H., Dikic I. (2013). Ubiquitination and selective autophagy. *Cell Death and Differentiation*.

[30] Kirkin V., McEwan D. G., Novak I., Dikic I. (2009). A role for ubiquitin in selective autophagy. *Molecular Cell*.

[31] Zaffagnini G., Martens S. (2016). Mechanisms of selective autophagy. *Journal of Molecular Biology*.

[32] Walker S. A., Ktistakis N. T. (2020). Autophagosome biogenesis machinery. *Journal of Molecular Biology*.

[33] Lai S. C., Devenish R. J. (2012). LC3-associated phagocytosis (LAP): connections with host autophagy. *Cell*.

[34] Khaminets A., Behl C., Dikic I. (2016). Ubiquitin-dependent and independent signals in selective autophagy. *Trends in Cell Biology*.

[35] Zhang M., Kenny S. J., Ge L., Xu K., Schekman R. (2015). Translocation of interleukin-1*β* into a vesicle intermediate in autophagy-mediated secretion. *eLife*.

[36] Stolz A., Ernst A., Dikic I. (2014). Cargo recognition and trafficking in selective autophagy. *Nature Cell Biology*.

[37] Fazeli G., Wehman A. M. (2017). Safely removing cell debris with LC3-associated phagocytosis. *Biology of the Cell*.

[38] Deng Z., Purtell K., Lachance V., Wold M. S., Chen S., Yue Z. (2017). Autophagy receptors and neurodegenerative diseases. *Trends in Cell Biology*.

[39] Pickford F., Masliah E., Britschgi M. (2008). The autophagy-related protein beclin 1 shows reduced expression in early Alzheimer disease and regulates amyloid beta accumulation in mice. *The Journal of Clinical Investigation*.

[40] Cho S. J., Yun S. M., Jo C. (2014). SUMO1 promotes A*β* production via the modulation of autophagy. *Autophagy*.

[41] Rui Y. N., Xu Z., Patel B. (2015). Huntingtin functions as a scaffold for selective macroautophagy. *Nature Cell Biology*.

[42] Stefanis L., Emmanouilidou E., Pantazopoulou M., Kirik D., Vekrellis K., Tofaris G. K. (2019). How is alpha-synuclein cleared from the cell?. *Journal of Neurochemistry*.

[43] Melia T. J., Lystad A. H., Simonsen A. (2020). Autophagosome biogenesis: from membrane growth to closure. *The Journal of Cell Biology*.

[44] Zachari M., Ganley I. G. (2017). The mammalian ULK1 complex and autophagy initiation. *Essays in Biochemistry*.

[45] Rabanal-Ruiz Y., Korolchuk V. I. (2018). mTORC1 and nutrient homeostasis: the central role of the lysosome. *International Journal of Molecular Sciences*.

[46] Papinski D., Kraft C. (2016). Regulation of autophagy by signaling through the Atg1/ULK1 complex. *Journal of Molecular Biology*.

[47] Egan D. F., Chun M. G. H., Vamos M. (2015). Small molecule inhibition of the autophagy kinase ULK1 and identification of ULK1 substrates. *Molecular Cell*.

[48] Yeh Y. Y., Wrasman K., Herman P. K. (2010). Autophosphorylation within the Atg1 activation loop is required for both kinase activity and the induction of autophagy in *Saccharomyces cerevisiae*. *Genetics*.

[49] Mercer T. J., Gubas A., Tooze S. A. (2018). A molecular perspective of mammalian autophagosome biogenesis. *The Journal of Biological Chemistry*.

[50] Ktistakis N. T. (2020). ER platforms mediating autophagosome generation. *Biochimica et Biophysica Acta - Molecular and Cell Biology of Lipids*.

[51] Wei Y., Liu M., Li X., Liu J., Li H. (2018). Origin of the autophagosome membrane in mammals. *BioMed Research International*.

[52] Rao Y., Perna M. G., Hofmann B., Beier V., Wollert T. (2016). The Atg1-kinase complex tethers Atg9-vesicles to initiate autophagy. *Nature Communications*.

[53] Karanasios E., Walker S. A., Okkenhaug H. (2016). Autophagy initiation by ULK complex assembly on ER tubulovesicular regions marked by ATG9 vesicles. *Nature Communications*.

[54] Zhou C., Ma K., Gao R. (2017). Regulation of mATG9 trafficking by Src- and ULK1-mediated phosphorylation in basal and starvation-induced autophagy. *Cell Research*.

[55] Johansen T., Lamark T. (2020). Selective autophagy: ATG8 family proteins, LIR motifs and cargo receptors. *Journal of Molecular Biology*.

[56] Matsunaga K., Morita E., Saitoh T. (2010). Autophagy requires endoplasmic reticulum targeting of the PI3-kinase complex via Atg14L. *The Journal of Cell Biology*.

[57] Cianfanelli V., de Zio D., di Bartolomeo S., Nazio F., Strappazzon F., Cecconi F. (2015). Ambra1 at a glance. *Journal of Cell Science*.

[58] Axe E. L., Walker S. A., Manifava M. (2008). Autophagosome formation from membrane compartments enriched in phosphatidylinositol 3-phosphate and dynamically connected to the endoplasmic reticulum. *The Journal of Cell Biology*.

[59] Hayashi-Nishino M., Fujita N., Noda T., Yamaguchi A., Yoshimori T., Yamamoto A. (2009). A subdomain of the endoplasmic reticulum forms a cradle for autophagosome formation. *Nature Cell Biology*.

[60] Cianfanelli V., D’Orazio M., Cecconi F. (2015). AMBRA1 and BECLIN 1 interplay in the crosstalk between autophagy and cell proliferation. *Cell Cycle*.

[61] Dragich J. M., Kuwajima T., Hirose-Ikeda M. (2016). Autophagy linked FYVE (Alfy/WDFY3) is required for establishing neuronal connectivity in the mammalian brain. *eLife*.

[62] Filimonenko M., Isakson P., Finley K. D. (2010). The selective macroautophagic degradation of aggregated proteins requires the PI3P-binding protein Alfy. *Molecular Cell*.

[63] Dooley H. C., Wilson M. I., Tooze S. A. (2015). WIPI2B links PtdIns3P to LC3 lipidation through binding ATG16L1. *Autophagy*.

[64] Fracchiolla D., Chang C., Hurley J. H., Martens S. (2020). A PI3K-WIPI2 positive feedback loop allosterically activates LC3 lipidation in autophagy. *The Journal of Cell Biology*.

[65] Geng J., Klionsky D. J. (2008). The Atg8 and Atg12 ubiquitin-like conjugation systems in macroautophagy. “Protein modifications: beyond the usual suspects” review series. *EMBO Reports*.

[66] Mizushima N., Noda T., Yoshimori T. (1998). A protein conjugation system essential for autophagy. *Nature*.

[67] Bai H., Inoue J., Kawano T., Inazawa J. (2012). A transcriptional variant of the LC3A gene is involved in autophagy and frequently inactivated in human cancers. *Oncogene*.

[68] Schaaf M. B. E., Keulers T. G., Vooijs M. A., Rouschop K. M. A. (2016). LC3/GABARAP family proteins: autophagy-(un)related functions. *The FASEB Journal*.

[69] Maruyama T., Noda N. N. (2017). Autophagy-regulating protease Atg4: structure, function, regulation and inhibition. *The Journal of Antibiotics*.

[70] Mizushima N. (2020). The ATG conjugation systems in autophagy. *Current Opinion in Cell Biology*.

[71] Lystad A. H., Carlsson S. R., Simonsen A. (2019). Toward the function of mammalian ATG12-ATG5-ATG16L1 complex in autophagy and related processes. *Autophagy*.

[72] Zhou F., Wu Z., Zhao M., Segev N., Liang Y. (2019). Autophagosome closure by ESCRT: Vps21/RAB5-regulated ESCRT recruitment via an Atg17-Snf7 interaction. *Autophagy*.

[73] Kishi-Itakura C., Koyama-Honda I., Itakura E., Mizushima N. (2014). Ultrastructural analysis of autophagosome organization using mammalian autophagy-deficient cells. *Journal of Cell Science*.

[74] Nguyen T. N., Padman B. S., Usher J., Oorschot V., Ramm G., Lazarou M. (2016). Atg8 family LC3/GABARAP proteins are crucial for autophagosome-lysosome fusion but not autophagosome formation during PINK1/Parkin mitophagy and starvation. *The Journal of Cell Biology*.

[75] Osawa T., Kotani T., Kawaoka T. (2019). Atg2 mediates direct lipid transfer between membranes for autophagosome formation. *Nature Structural & Molecular Biology*.

[76] Bozic M., van den Bekerom L., Milne B. A. (2020). A conserved ATG2-GABARAP family interaction is critical for phagophore formation. *EMBO Reports*.

[77] Pankiv S., Alemu E. A., Brech A. (2010). FYCO1 is a Rab7 effector that binds to LC3 and PI3P to mediate microtubule plus end-directed vesicle transport. *The Journal of Cell Biology*.

[78] Nakamura S., Yoshimori T. (2017). New insights into autophagosome-lysosome fusion. *Journal of Cell Science*.

[79] Harrison R. E., Bucci C., Vieira O. V., Schroer T. A., Grinstein S. (2003). Phagosomes fuse with late endosomes and/or lysosomes by extension of membrane protrusions along microtubules: role of Rab7 and RILP. *Molecular and Cellular Biology*.

[80] Köchl R., Hu X. W., Chan E. Y. W., Tooze S. A. (2006). Microtubules facilitate autophagosome formation and fusion of autophagosomes with endosomes. *Traffic*.

[81] Fass E., Shvets E., Degani I., Hirschberg K., Elazar Z. (2006). Microtubules support production of starvation-induced autophagosomes but not their targeting and fusion with lysosomes. *The Journal of Biological Chemistry*.

[82] Jahreiss L., Menzies F. M., Rubinsztein D. C. (2008). The itinerary of autophagosomes: from peripheral formation to kiss-and-run fusion with lysosomes. *Traffic*.

[83] Joachim J., Razi M., Judith D. (2017). Centriolar satellites control GABARAP ubiquitination and GABARAP-mediated autophagy. *Current Biology*.

[84] Jiang P., Nishimura T., Sakamaki Y. (2014). The HOPS complex mediates autophagosome-lysosome fusion through interaction with syntaxin 17. *Molecular Biology of the Cell*.

[85] Reggiori F., Ungermann C. (2017). Autophagosome maturation and fusion. *Journal of Molecular Biology*.

[86] Diao J., Liu R., Rong Y. (2015). ATG14 promotes membrane tethering and fusion of autophagosomes to endolysosomes. *Nature*.

[87] Itakura E., Kishi-Itakura C., Mizushima N. (2012). The hairpin-type tail-anchored SNARE syntaxin 17 targets to autophagosomes for fusion with endosomes/lysosomes. *Cell*.

[88] Kriegenburg F., Ungermann C., Reggiori F. (2018). Coordination of autophagosome-lysosome fusion by Atg8 family members. *Current Biology*.

[89] Wilkinson D. S., Jariwala J. S., Anderson E. (2015). Phosphorylation of LC3 by the Hippo kinases STK3/STK4 is essential for autophagy. *Molecular Cell*.

[90] Wang C., Wang H., Zhang D. (2018). Phosphorylation of ULK1 affects autophagosome fusion and links chaperone-mediated autophagy to macroautophagy. *Nature Communications*.

[91] Lieberman O. J., Sulzer D. (2019). The synaptic autophagy cycle. *Journal of Molecular Biology*.

[92] Lim J., Yue Z. (2015). Neuronal aggregates: formation, clearance, and spreading. *Developmental Cell*.

[93] Walden H., Muqit M. M. K. (2017). Ubiquitin and Parkinson’s disease through the looking glass of genetics. *The Biochemical Journal*.

[94] Hyttinen J. M. T., Amadio M., Viiri J., Pascale A., Salminen A., Kaarniranta K. (2014). Clearance of misfolded and aggregated proteins by aggrephagy and implications for aggregation diseases. *Ageing Research Reviews*.

[95] Waguri S., Komatsu M. (2009). Chapter 9. Biochemical and morphological detection of inclusion bodies in autophagy-deficient mice. *Methods in Enzymology*.

[96] Sato S., Uchihara T., Fukuda T. (2018). Loss of autophagy in dopaminergic neurons causes Lewy pathology and motor dysfunction in aged mice. *Scientific Reports*.

[97] Kulkarni A., Chen J., Maday S. (2018). Neuronal autophagy and intercellular regulation of homeostasis in the brain. *Current Opinion in Neurobiology*.

[98] Lieberman O. J., McGuirt A. F., Tang G., Sulzer D. (2019). Roles for neuronal and glial autophagy in synaptic pruning during development. *Neurobiology of Disease*.

[99] Ban B. K., Jun M. H., Ryu H. H., Jang D. J., Ahmad S. T., Lee J. A. (2013). Autophagy negatively regulates early axon growth in cortical neurons. *Molecular and Cellular Biology*.

[100] Hernandez D., Torres C. A., Setlik W. (2012). Regulation of presynaptic neurotransmission by macroautophagy. *Neuron*.

[101] Nixon R. A. (2013). The role of autophagy in neurodegenerative disease. *Nature Medicine*.

[102] Komatsu M., Wang Q. J., Holstein G. R. (2007). Essential role for autophagy protein Atg7 in the maintenance of axonal homeostasis and the prevention of axonal degeneration. *Proceedings of the National Academy of Sciences of the United States of America*.

[103] Chu C. T. (2019). Mechanisms of selective autophagy and mitophagy: implications for neurodegenerative diseases. *Neurobiology of Disease*.

[104] Yoshii S. R., Kuma A., Akashi T. (2016). Systemic analysis of Atg5-null mice rescued from neonatal lethality by transgenic ATG5 expression in neurons. *Developmental Cell*.

[105] Ramirez-Moreno M. J., Duarte-Jurado A. P., Gopar-Cuevas Y. (2019). Autophagy stimulation decreases dopaminergic neuronal death mediated by oxidative stress. *Molecular Neurobiology*.

[106] Barmada S. J., Serio A., Arjun A. (2014). Autophagy induction enhances TDP43 turnover and survival in neuronal ALS models. *Nature Chemical Biology*.

[107] Hara T., Nakamura K., Matsui M. (2006). Suppression of basal autophagy in neural cells causes neurodegenerative disease in mice. *Nature*.

[108] Komatsu M., Waguri S., Chiba T. (2006). Loss of autophagy in the central nervous system causes neurodegeneration in mice. *Nature*.

[109] Frake R. A., Ricketts T., Menzies F. M., Rubinsztein D. C. (2015). Autophagy and neurodegeneration. *The Journal of Clinical Investigation*.

[110] Maday S., Holzbaur E. L. F. (2016). Compartment-specific regulation of autophagy in primary neurons. *The Journal of Neuroscience*.

[111] Maday S., Holzbaur E. L. F. (2014). Autophagosome biogenesis in primary neurons follows an ordered and spatially regulated pathway. *Developmental Cell*.

[112] Maday S., Wallace K. E., Holzbaur E. L. F. (2012). Autophagosomes initiate distally and mature during transport toward the cell soma in primary neurons. *The Journal of Cell Biology*.

[113] Ashrafi G., Schlehe J. S., LaVoie M. J., Schwarz T. L. (2014). Mitophagy of damaged mitochondria occurs locally in distal neuronal axons and requires PINK1 and Parkin. *The Journal of Cell Biology*.

[114] Fu M. M., Holzbaur E. L. F. (2014). MAPK8IP1/JIP1 regulates the trafficking of autophagosomes in neurons. *Autophagy*.

[115] Stavoe A. K. H., Hill S. E., Hall D. H., Colón-Ramos D. A. (2016). KIF1A/UNC-104 transports ATG-9 to regulate neurodevelopment and autophagy at synapses. *Developmental Cell*.

[116] Quadri M., Fang M., Picillo M. (2013). Mutation in the SYNJ1 gene associated with autosomal recessive, early-onset Parkinsonism. *Human Mutation*.

[117] Schuske K. R., Richmond J. E., Matthies D. S. (2003). Endophilin is required for synaptic vesicle endocytosis by localizing synaptojanin. *Neuron*.

[118] Soukup S. F., Kuenen S., Vanhauwaert R. (2016). A LRRK2-dependent endophilin A phosphoswitch is critical for macroautophagy at presynaptic terminals. *Neuron*.

[119] Vanhauwaert R., Kuenen S., Masius R. (2017). The SAC1 domain in synaptojanin is required for autophagosome maturation at presynaptic terminals. *The EMBO Journal*.

[120] Okerlund N. D., Schneider K., Leal-Ortiz S. (2017). Bassoon controls presynaptic autophagy through Atg5. *Neuron*.

[121] Shehata M., Matsumura H., Okubo-Suzuki R., Ohkawa N., Inokuchi K. (2012). Neuronal stimulation induces autophagy in hippocampal neurons that is involved in AMPA receptor degradation after chemical long-term depression. *The Journal of Neuroscience*.

[122] Pfeffer S. R. (2010). Unconventional secretion by autophagosome exocytosis. *The Journal of Cell Biology*.

[123] Sulzer D., Mosharov E., Talloczy Z., Zucca F. A., Simon J. D., Zecca L. (2008). Neuronal pigmented autophagic vacuoles: lipofuscin, neuromelanin, and ceroid as macroautophagic responses during aging and disease. *Journal of Neurochemistry*.

[124] Stavoe A. K., Gopal P. P., Gubas A., Tooze S. A., Holzbaur E. L. F. (2019). Expression of WIPI2B counteracts age-related decline in autophagosome biogenesis in neurons. *eLife*.

[125] Ryan B. J., Hoek S., Fon E. A., Wade-Martins R. (2015). Mitochondrial dysfunction and mitophagy in Parkinson’s: from familial to sporadic disease. *Trends in Biochemical Sciences*.

[126] Liu J., Liu W., Li R., Yang H. (2019). Mitophagy in Parkinson’s disease: from pathogenesis to treatment. *Cell*.

[127] Anding A. L., Baehrecke E. H. (2017). Cleaning house: selective autophagy of organelles. *Developmental Cell*.

[128] Van Houten B., Hunter S. E., Meyer J. N. (2016). Mitochondrial DNA damage induced autophagy, cell death, and disease. *Frontiers in Bioscience*.

[129] Zhao R. Z., Jiang S., Zhang L., Yu Z. B. (2019). Mitochondrial electron transport chain, ROS generation and uncoupling (review). *International Journal of Molecular Medicine*.

[130] Youle R. J., van der Bliek A. M. (2012). Mitochondrial fission, fusion, and stress. *Science*.

[131] von Stockum S., Marchesan E., Ziviani E. (2018). Mitochondrial quality control beyond PINK1/Parkin. *Oncotarget*.

[132] Strappazzon F., Nazio F., Corrado M. (2015). AMBRA1 is able to induce mitophagy via LC3 binding, regardless of PARKIN and p62/SQSTM1. *Cell Death & Differentiation*.

[133] Di Rita A., Peschiaroli A., D’Acunzo P. (2018). HUWE1 E3 ligase promotes PINK1/PARKIN-independent mitophagy by regulating AMBRA1 activation via IKK*α*. *Nature Communications*.

[134] Chu C. T., Ji J., Dagda R. K. (2013). Cardiolipin externalization to the outer mitochondrial membrane acts as an elimination signal for mitophagy in neuronal cells. *Nature Cell Biology*.

[135] Princely Abudu Y., Pankiv S., Mathai B. J. (2019). NIPSNAP1 and NIPSNAP2 act as “Eat Me” signals for mitophagy. *Developmental Cell*.

[136] Sekine S., Youle R. J. (2018). PINK1 import regulation; a fine system to convey mitochondrial stress to the cytosol. *BMC Biology*.

[137] Yamano K., Matsuda N., Tanaka K. (2016). The ubiquitin signal and autophagy: an orchestrated dance leading to mitochondrial degradation. *EMBO Reports*.

[138] Geisler S., Holmström K. M., Skujat D. (2010). PINK1/Parkin-mediated mitophagy is dependent on VDAC1 and p62/SQSTM1. *Nature Cell Biology*.

[139] Burchell V. S., Nelson D. E., Sanchez-Martinez A. (2013). The Parkinson’s disease-linked proteins Fbxo7 and Parkin interact to mediate mitophagy. *Nature Neuroscience*.

[140] Zachari M., Gudmundsson S. R., Li Z. (2019). Selective autophagy of mitochondria on a ubiquitin-endoplasmic-reticulum platform. *Developmental Cell*.

[141] Richter B., Sliter D. A., Herhaus L. (2016). Phosphorylation of OPTN by TBK1 enhances its binding to Ub chains and promotes selective autophagy of damaged mitochondria. *Proceedings of the National Academy of Sciences of the United States of America*.

[142] Matsumoto G., Shimogori T., Hattori N., Nukina N. (2015). TBK1 controls autophagosomal engulfment of polyubiquitinated mitochondria through p62/SQSTM1 phosphorylation. *Human Molecular Genetics*.

[143] Lazarou M., Sliter D. A., Kane L. A. (2015). The ubiquitin kinase PINK1 recruits autophagy receptors to induce mitophagy. *Nature*.

[144] Turco E., Witt M., Abert C. (2019). FIP200 claw domain binding to p62 promotes autophagosome formation at ubiquitin condensates. *Molecular Cell*.

[145] Antón Z., Landajuela A., Hervás J. H. (2016). Human Atg8-cardiolipin interactions in mitophagy: specific properties of LC3B, GABARAPL2 and GABARAP. *Autophagy*.

[146] Kagan V. E., Jiang J., Huang Z. (2016). NDPK-D (NM23-H4)-mediated externalization of cardiolipin enables elimination of depolarized mitochondria by mitophagy. *Cell Death and Differentiation*.

[147] Rogov V., Dötsch V., Johansen T., Kirkin V. (2014). Interactions between autophagy receptors and ubiquitin-like proteins form the molecular basis for selective autophagy. *Molecular Cell*.

[148] Cho D. H., Kim Y. S., Jo D. S., Choe S. K., Jo E. K. (2018). Pexophagy: molecular mechanisms and implications for health and diseases. *Molecules and Cells*.

[149] Germain K., Kim P. K. (2020). Pexophagy: a model for selective autophagy. *International Journal of Molecular Sciences*.

[150] Uzor N. E., McCullough L. D., Tsvetkov A. S. (2020). Peroxisomal dysfunction in neurological diseases and brain aging. *Frontiers in Cellular Neuroscience*.

[151] Conway O., Akpinar H. A., Rogov V. V., Kirkin V. (2019). Selective autophagy receptors in neuronal health and disease. *Journal of Molecular Biology*.

[152] Smith M. D., Harley M. E., Kemp A. J. (2018). CCPG1 is a non-canonical autophagy cargo receptor essential for ER-phagy and pancreatic ER proteostasis. *Developmental Cell*.

[153] Chino H., Hatta T., Natsume T., Mizushima N. (2019). Intrinsically disordered protein TEX264 mediates ER-phagy. *Molecular Cell*.

[154] An H., Ordureau A., Paulo J. A., Shoemaker C. J., Denic V., Harper J. W. (2019). TEX264 is an endoplasmic reticulum-resident ATG8-interacting protein critical for ER remodeling during nutrient stress. *Molecular Cell*.

[155] Fumagalli F., Noack J., Bergmann T. J. (2016). Translocon component Sec62 acts in endoplasmic reticulum turnover during stress recovery. *Nature Cell Biology*.

[156] Khaminets A., Heinrich T., Mari M. (2015). Regulation of endoplasmic reticulum turnover by selective autophagy. *Nature*.

[157] Chen Q., Xiao Y., Chai P., Zheng P., Teng J., Chen J. (2019). ATL3 is a tubular ER-phagy receptor for GABARAP-mediated selective autophagy. *Current Biology*.

[158] Grumati P., Morozzi G., Hölper S. (2017). Full length RTN3 regulates turnover of tubular endoplasmic reticulum via selective autophagy. *eLife*.

[159] Liang J. R., Lingeman E., Luong T. (2020). A genome-wide ER-phagy screen highlights key roles of mitochondrial metabolism and ER-resident UFmylation. *Cell*.

[160] Nthiga T. M., Kumar Shrestha B., Sjøttem E. (2020). CALCOCO1 acts with VAMP-associated proteins to mediate ER-phagy. *The EMBO Journal*.

[161] Smith M., Wilkinson S. (2017). ER homeostasis and autophagy. *Essays in Biochemistry*.

[162] Dikic I. (2018). Open questions: why should we care about ER-phagy and ER remodelling?. *BMC Biology*.

[163] Senft D., Ronai Z. A. (2015). UPR, autophagy, and mitochondria crosstalk underlies the ER stress response. *Trends in Biochemical Sciences*.

[164] Borodkina A. V., Shatrova A. N., Deryabin P. I. (2016). Calcium alterations signal either to senescence or to autophagy induction in stem cells upon oxidative stress. *Aging (Albany NY)*.

[165] Høyer-Hansen M., Bastholm L., Szyniarowski P. (2007). Control of macroautophagy by calcium, calmodulin-dependent kinase kinase-beta, and Bcl-2. *Molecular Cell*.

[166] Zalckvar E., Berissi H., Eisenstein M., Kimchi A. (2014). Phosphorylation of Beclin 1 by DAP-kinase promotes autophagy by weakening its interactions with Bcl-2 and Bcl-XL. *Autophagy*.

[167] Simon B., Huart A. S., Temmerman K. (2016). Death-associated protein kinase activity is regulated by coupled calcium/calmodulin binding to two distinct sites. *Structure*.

[168] Ber Y., Shiloh R., Gilad Y., Degani N., Bialik S., Kimchi A. (2015). DAPK2 is a novel regulator of mTORC1 activity and autophagy. *Cell Death and Differentiation*.

[169] Jiang X., Wang X., Ding X. (2020). FAM134B oligomerization drives endoplasmic reticulum membrane scission for ER-phagy. *The EMBO Journal*.

[170] B’chir W., Chaveroux C., Carraro V. (2014). Dual role for CHOP in the crosstalk between autophagy and apoptosis to determine cell fate in response to amino acid deprivation. *Cellular Signalling*.

[171] B’chir W., Maurin A.-C., Carraro V. (2013). The eIF2*α*/ATF4 pathway is essential for stress-induced autophagy gene expression. *Nucleic Acids Research*.

[172] Song S., Tan J., Miao Y., Zhang Q. (2018). Crosstalk of ER stress-mediated autophagy and ER-phagy: involvement of UPR and the core autophagy machinery. *Journal of Cellular Physiology*.

[173] Wilkinson S. (2019). ER-phagy: shaping up and destressing the endoplasmic reticulum. *The FEBS Journal*.

[174] Chino H., Mizushima N. (2020). ER-phagy: quality control and turnover of endoplasmic reticulum. *Trends in Cell Biology*.

[175] Grumati P., Dikic I., Stolz A. (2018). ER-phagy at a glance. *Journal of Cell Science*.

[176] Karagas N. E., Venkatachalam K. (2019). Roles for the endoplasmic reticulum in regulation of neuronal calcium homeostasis. *Cell*.

[177] Öztürk Z., O’Kane C. J., Pérez-Moreno J. J. (2020). Axonal endoplasmic reticulum dynamics and its roles in neurodegeneration. *Frontiers in Neuroscience*.

[178] Paillusson S., Stoica R., Gomez-Suaga P. (2016). There’s something wrong with my MAM; the ER-mitochondria axis and neurodegenerative diseases. *Trends in Neurosciences*.

[179] Martínez G., Vidal R. L., Mardones P. (2016). Regulation of memory formation by the transcription factor XBP1. *Cell Reports*.

[180] Trinh M. A., Klann E. (2013). Translational control by eIF2*α* kinases in long-lasting synaptic plasticity and long-term memory. *Neurobiology of Learning and Memory*.

[181] Saito A., Cai L., Matsuhisa K. (2018). Neuronal activity-dependent local activation of dendritic unfolded protein response promotes expression of brain-derived neurotrophic factor in cell soma. *Journal of Neurochemistry*.

[182] Oñate M., Court F. A., Hetz C. (2016). Bursting the unfolded protein response accelerates axonal regeneration. *Neural Regeneration Research*.

[183] Zou Y., He W., Wang K. (2018). Identification of rare RTN3 variants in Alzheimer’s disease in Han Chinese. *Human Genetics*.

[184] Park S., Aintablian A., Coupe B., Bouret S. G. (2020). The endoplasmic reticulum stress-autophagy pathway controls hypothalamic development and energy balance regulation in leptin-deficient neonates. *Nature Communications*.

[185] Phaniendra A., Jestadi D. B., Periyasamy L. (2015). Free radicals: properties, sources, targets, and their implication in various diseases. *Indian Journal of Clinical Biochemistry*.

[186] Sies H., Jones D. P. (2020). Reactive oxygen species (ROS) as pleiotropic physiological signalling agents. *Nature Reviews Molecular Cell Biology*.

[187] Wanders R. J., Waterham H. R., Ferdinandusse S. (2016). Metabolic interplay between peroxisomes and other subcellular organelles including mitochondria and the endoplasmic reticulum. *Frontiers in Cell and Development Biology*.

[188] di Meo S., Reed T. T., Venditti P., Victor V. M. (2016). Role of ROS and RNS sources in physiological and pathological conditions. *Oxidative Medicine and Cellular Longevity*.

[189] Salim S. (2016). Oxidative stress and the central nervous system. *The Journal of Pharmacology and Experimental Therapeutics*.

[190] Keane P. C., Kurzawa M., Blain P. G., Morris C. M. (2011). Mitochondrial dysfunction in Parkinson’s disease. *Parkinson’s Disease*.

[191] Liu Y., Fiskum G., Schubert D. (2002). Generation of reactive oxygen species by the mitochondrial electron transport chain. *Journal of Neurochemistry*.

[192] Zorov D. B., Juhaszova M., Sollott S. J. (2014). Mitochondrial reactive oxygen species (ROS) and ROS-induced ROS release. *Physiological Reviews*.

[193] Andreyev A. Y., Kushnareva Y. E., Murphy A. N., Starkov A. A. (2015). Mitochondrial ROS metabolism: 10 years later. *Biochemistry*.

[194] Osumi T., Hashimoto T. (1978). Acyl-CoA oxidase of rat liver: a new enzyme for fatty acid oxidation. *Biochemical and Biophysical Research Communications*.

[195] Kelley E. E., Khoo N. K. H., Hundley N. J., Malik U. Z., Freeman B. A., Tarpey M. M. (2010). Hydrogen peroxide is the major oxidant product of xanthine oxidase. *Free Radical Biology & Medicine*.

[196] Harrison R. (2002). Structure and function of xanthine oxidoreductase: where are we now?. *Free Radical Biology & Medicine*.

[197] Yoboue E. D., Sitia R., Simmen T. (2018). Redox crosstalk at endoplasmic reticulum (ER) membrane contact sites (MCS) uses toxic waste to deliver messages. *Cell Death & Disease*.

[198] Schrader M., Fahimi H. D. (2006). Peroxisomes and oxidative stress. *Biochimica et Biophysica Acta (BBA) - Molecular Cell Research*.

[199] Zeeshan H., Lee G., Kim H. R., Chae H. J. (2016). Endoplasmic reticulum stress and associated ROS. *International Journal of Molecular Sciences*.

[200] Kodali V. K., Thorpe C. (2010). Oxidative protein folding and the Quiescin-sulfhydryl oxidase family of flavoproteins. *Antioxidants & Redox Signaling*.

[201] Kondoh M., Ohga N., Akiyama K. (2013). Hypoxia-induced reactive oxygen species cause chromosomal abnormalities in endothelial cells in the tumor microenvironment. *PLoS One*.

[202] Fuhrmann D. C., Brune B. (2017). Mitochondrial composition and function under the control of hypoxia. *Redox Biology*.

[203] Chittiboyina S., Bai Y., Lelievre S. A. (2018). Microenvironment-cell nucleus relationship in the context of oxidative stress. *Frontiers in Cell and Development Biology*.

[204] Davalli P., Mitic T., Caporali A., Lauriola A., D’Arca D. (2016). ROS, cell senescence, and novel molecular mechanisms in aging and age-related diseases. *Oxidative Medicine and Cellular Longevity*.

[205] López-Otín C., Blasco M. A., Partridge L., Serrano M., Kroemer G. (2013). The hallmarks of aging. *Cell*.

[206] Bae Y. S., Oh H., Rhee S. G., Yoo Y. D. (2011). Regulation of reactive oxygen species generation in cell signaling. *Molecules and Cells*.

[207] Zhang J., Wang X., Vikash V. (2016). ROS and ROS-mediated cellular signaling. *Oxidative Medicine and Cellular Longevity*.

[208] Ray P. D., Huang B. W., Tsuji Y. (2012). Reactive oxygen species (ROS) homeostasis and redox regulation in cellular signaling. *Cellular Signalling*.

[209] Gasperini R. J., Pavez M., Thompson A. C. (2017). How does calcium interact with the cytoskeleton to regulate growth cone motility during axon pathfinding?. *Molecular and Cellular Neurosciences*.

[210] Olguin-Albuerne M., Moran J. (2018). Redox signaling mechanisms in nervous system development. *Antioxidants & Redox Signaling*.

[211] Oswald M. C. W., Garnham N., Sweeney S. T., Landgraf M. (2018). Regulation of neuronal development and function by ROS. *FEBS Letters*.

[212] Beckhauser T. F., Francis-Oliveira J., De Pasquale R. (2016). Reactive oxygen species: physiological and physiopathological effects on synaptic plasticity. *Journal of Experimental Neuroscience*.

[213] Massaad C. A., Klann E. (2011). Reactive oxygen species in the regulation of synaptic plasticity and memory. *Antioxidants & Redox Signaling*.

[214] Fernandez-Fernandez S., Almeida A., Bolanos J. P. (2012). Antioxidant and bioenergetic coupling between neurons and astrocytes. *The Biochemical Journal*.

[215] Atkins C. M., Sweatt J. D. (1999). Reactive oxygen species mediate activity-dependent neuron-glia signaling in output fibers of the hippocampus. *The Journal of Neuroscience*.

[216] Tamma G., Valenti G., Grossini E. (2018). Aquaporin membrane channels in oxidative stress, cell signaling, and aging: recent advances and research trends. *Oxidative Medicine and Cellular Longevity*.

[217] Dawson V. L., Dawson T. M. (1996). Free radicals and neuronal cell death. *Cell Death and Differentiation*.

[218] Silva J. P., Coutinho O. P. (2010). Free radicals in the regulation of damage and cell death—basic mechanisms and prevention. *Drug Discoveries & Therapeutics*.

[219] Salisbury D., Bronas U. (2015). Reactive oxygen and nitrogen species: impact on endothelial dysfunction. *Nursing Research*.

[220] Park E., Chung S. W. (2019). ROS-mediated autophagy increases intracellular iron levels and ferroptosis by ferritin and transferrin receptor regulation. *Cell Death & Disease*.

[221] Lévy E., el Banna N., Baïlle D. (2019). Causative links between protein aggregation and oxidative stress: a review. *International Journal of Molecular Sciences*.

[222] Dexter D. T., Carter C. J., Wells F. R. (1989). Basal lipid peroxidation in substantia nigra is increased in Parkinson’s disease. *Journal of Neurochemistry*.

[223] Kreuz S., Fischle W. (2016). Oxidative stress signaling to chromatin in health and disease. *Epigenomics*.

[224] Sadikovic B., al-Romaih K., Squire J., Zielenska M. (2008). Cause and consequences of genetic and epigenetic alterations in human cancer. *Current Genomics*.

[225] Perl A. (2013). Oxidative stress in the pathology and treatment of systemic lupus erythematosus. *Nature Reviews Rheumatology*.

[226] Ivashchenko O., van Veldhoven P. P., Brees C., Ho Y. S., Terlecky S. R., Fransen M. (2011). Intraperoxisomal redox balance in mammalian cells: oxidative stress and interorganellar cross-talk. *Molecular Biology of the Cell*.

[227] Janikiewicz J., Szymański J., Malinska D. (2018). Mitochondria-associated membranes in aging and senescence: structure, function, and dynamics. *Cell Death & Disease*.

[228] Hamasaki M., Furuta N., Matsuda A. (2013). Autophagosomes form at ER-mitochondria contact sites. *Nature*.

[229] Yang J. Y., Yang W. Y. (2013). Bit-by-bit autophagic removal of parkin-labelled mitochondria. *Nature Communications*.

[230] Redza-Dutordoir M., Averill-Bates D. A. (2016). Activation of apoptosis signalling pathways by reactive oxygen species. *Biochimica et Biophysica Acta (BBA) - Molecular Cell Research*.

[231] Cobley J. N., Fiorello M. L., Bailey D. M. (2018). 13 reasons why the brain is susceptible to oxidative stress. *Redox Biology*.

[232] Haorah J., Ramirez S. H., Schall K., Smith D., Pandya R., Persidsky Y. (2007). Oxidative stress activates protein tyrosine kinase and matrix metalloproteinases leading to blood-brain barrier dysfunction. *Journal of Neurochemistry*.

[233] Stincone A., Prigione A., Cramer T. (2015). The return of metabolism: biochemistry and physiology of the pentose phosphate pathway. *Biological Reviews of the Cambridge Philosophical Society*.

[234] Espinosa-Diez C., Miguel V., Mennerich D. (2015). Antioxidant responses and cellular adjustments to oxidative stress. *Redox Biology*.

[235] Pajares M., Cuadrado A., Engedal N., Jirsova Z., Cahova M. (2018). The role of free radicals in autophagy regulation: implications for ageing. *Oxidative Medicine and Cellular Longevity*.

[236] Mailloux R. J. (2018). Mitochondrial antioxidants and the maintenance of cellular hydrogen peroxide levels. *Oxidative Medicine and Cellular Longevity*.

[237] Sandalio L. M., Romero-Puertas M. C. (2015). Peroxisomes sense and respond to environmental cues by regulating ROS and RNS signalling networks. *Annals of Botany*.

[238] Ighodaro O. M., Akinloye O. A. (2018). First line defence antioxidants—superoxide dismutase (SOD), catalase (CAT) and glutathione peroxidase (GPX): their fundamental role in the entire antioxidant defence grid. *Alexandria Journal of Medicine*.

[239] Grant C. M. (2008). Metabolic reconfiguration is a regulated response to oxidative stress. *Journal of Biology*.

[240] Vomund S., Schäfer A., Parnham M., Brüne B., von Knethen A. (2017). Nrf2, the master regulator of anti-oxidative responses. *International Journal of Molecular Sciences*.

[241] Yamamoto M., Kensler T. W., Motohashi H. (2018). The KEAP1-NRF2 system: a thiol-based sensor-effector apparatus for maintaining redox homeostasis. *Physiological Reviews*.

[242] Cuadrado A., Rojo A. I., Wells G. (2019). Therapeutic targeting of the NRF2 and KEAP1 partnership in chronic diseases. *Nature Reviews Drug Discovery*.

[243] Schmidlin C. J., Dodson M. B., Madhavan L., Zhang D. D. (2019). Redox regulation by NRF2 in aging and disease. *Free Radical Biology & Medicine*.

[244] Homma K., Katagiri K., Nishitoh H., Ichijo H. (2009). Targeting ASK1 in ER stress-related neurodegenerative diseases. *Expert Opinion on Therapeutic Targets*.

[245] Tebay L. E., Robertson H., Durant S. T. (2015). Mechanisms of activation of the transcription factor Nrf2 by redox stressors, nutrient cues, and energy status and the pathways through which it attenuates degenerative disease. *Free Radical Biology & Medicine*.

[246] Yamazaki H., Tanji K., Wakabayashi K., Matsuura S., Itoh K. (2015). Role of the Keap1/Nrf2 pathway in neurodegenerative diseases. *Pathology International*.

[247] Hayes J. D., Dinkova-Kostova A. T. (2014). The Nrf2 regulatory network provides an interface between redox and intermediary metabolism. *Trends in Biochemical Sciences*.

[248] Garza-Lombó C., Pappa A., Panayiotidis M. I., Franco R. (2020). Redox homeostasis, oxidative stress and mitophagy. *Mitochondrion*.

[249] Montagna C., Rizza S., Maiani E., Piredda L., Filomeni G., Cecconi F. (2016). To eat, or NOt to eat: S-nitrosylation signaling in autophagy. *The FEBS Journal*.

[250] Tegeder I. (2019). Nitric oxide mediated redox regulation of protein homeostasis. *Cellular Signalling*.

[251] Hill B. G., Haberzettl P., Ahmed Y., Srivastava S., Bhatnagar A. (2008). Unsaturated lipid peroxidation-derived aldehydes activate autophagy in vascular smooth-muscle cells. *The Biochemical Journal*.

[252] Haberzettl P., Hill B. G. (2013). Oxidized lipids activate autophagy in a JNK-dependent manner by stimulating the endoplasmic reticulum stress response. *Redox Biology*.

[253] Galati S., Boni C., Gerra M. C., Lazzaretti M., Buschini A. (2019). Autophagy: a player in response to oxidative stress and DNA damage. *Oxidative Medicine and Cellular Longevity*.

[254] Meijles D. N., Zoumpoulidou G., Markou T. (2019). The cardiomyocyte “redox rheostat”: redox signalling via the AMPK-mTOR axis and regulation of gene and protein expression balancing survival and death. *Journal of Molecular and Cellular Cardiology*.

[255] Soubannier V., McLelland G. L., Zunino R. (2012). A vesicular transport pathway shuttles cargo from mitochondria to lysosomes. *Current Biology*.

[256] Dustin C. M., Heppner D. E., Lin M. C. J., van der Vliet A. (2020). Redox regulation of tyrosine kinase signalling: more than meets the eye. *Journal of Biochemistry*.

[257] Kim J. H., Choi T. G., Park S. (2018). Mitochondrial ROS-derived PTEN oxidation activates PI3K pathway for mTOR-induced myogenic autophagy. *Cell Death and Differentiation*.

[258] Delgado-Esteban M., Martin-Zanca D., Andres-Martin L., Almeida A., Bolaños J. P. (2007). Inhibition of PTEN by peroxynitrite activates the phosphoinositide-3-kinase/Akt neuroprotective signaling pathway. *Journal of Neurochemistry*.

[259] Zhang Y., Park J., Han S. J. (2019). Peroxiredoxin III protects tumor suppressor PTEN from oxidation by 15-hydroperoxy-eicosatetraenoic acid. *Oxidative Medicine and Cellular Longevity*.

[260] Liu J. Z., Duan J., Ni M. (2017). S-nitrosylation inhibits the kinase activity of tomato phosphoinositide-dependent kinase 1 (PDK1). *The Journal of Biological Chemistry*.

[261] Su Z., Burchfield J. G., Yang P. (2019). Global redox proteome and phosphoproteome analysis reveals redox switch in Akt. *Nature Communications*.

[262] Liu X., Jann J., Xavier C., Wu H. (2015). Glutaredoxin 1 (Grx1) protects human retinal pigment epithelial cells from oxidative damage by preventing AKT glutathionylation. *Investigative Ophthalmology & Visual Science*.

[263] Lopez-Rivera E., Jayaraman P., Parikh F. (2014). Inducible nitric oxide synthase drives mTOR pathway activation and proliferation of human melanoma by reversible nitrosylation of TSC2. *Cancer Research*.

[264] Oka S. I., Hirata T., Suzuki W. (2017). Thioredoxin-1 maintains mechanistic target of rapamycin (mTOR) function during oxidative stress in cardiomyocytes. *The Journal of Biological Chemistry*.

[265] Yu J., Yang J. (2019). Ion channels as potential redox sensors in lysosomes. *Channels*.

[266] Shao D., Oka S. I., Liu T. (2014). A redox-dependent mechanism for regulation of AMPK activation by Thioredoxin1 during energy starvation. *Cell Metabolism*.

[267] Hinchy E. C., Gruszczyk A. V., Willows R. (2018). Mitochondria-derived ROS activate AMP-activated protein kinase (AMPK) indirectly. *The Journal of Biological Chemistry*.

[268] Mungai P. T., Waypa G. B., Jairaman A. (2011). Hypoxia triggers AMPK activation through reactive oxygen species-mediated activation of calcium release-activated calcium channels. *Molecular and Cellular Biology*.

[269] Li L., Chen Y., Gibson S. B. (2013). Starvation-induced autophagy is regulated by mitochondrial reactive oxygen species leading to AMPK activation. *Cellular Signalling*.

[270] Hardie D. G. (2011). AMPK and autophagy get connected. *The EMBO Journal*.

[271] Tripathi D. N., Zhang J., Jing J., Dere R., Walker C. L. (2016). A new role for ATM in selective autophagy of peroxisomes (pexophagy). *Autophagy*.

[272] Zhang J., Tripathi D. N., Jing J. (2015). ATM functions at the peroxisome to induce pexophagy in response to ROS. *Nature Cell Biology*.

[273] Zhang Y., Lee J. H., Paull T. T. (2018). Mitochondrial redox sensing by the kinase ATM maintains cellular antioxidant capacity. *Science Signaling*.

[274] Tripathi D. N., Chowdhury R., Trudel L. J. (2013). Reactive nitrogen species regulate autophagy through ATM-AMPK-TSC2-mediated suppression of mTORC1. *Proceedings of the National Academy of Sciences of the United States of America*.

[275] Guo Z., Kozlov S., Lavin M. F., Person M. D., Paull T. T. (2010). ATM activation by oxidative stress. *Science*.

[276] Kitada M., Ogura Y., Koya D., Hayat M. A. (2016). Chapter 3. Role of Sirt1 as a regulator of autophagy. *Autophagy: Cancer, Other Pathologies, Inflammation, Immunity, Infection, and Aging*.

[277] Huang R., Xu Y., Wan W. (2015). Deacetylation of nuclear LC3 drives autophagy initiation under starvation. *Molecular Cell*.

[278] Lee I. H., Cao L., Mostoslavsky R. (2008). A role for the NAD-dependent deacetylase Sirt1 in the regulation of autophagy. *Proceedings of the National Academy of Sciences of the United States of America*.

[279] Price N. L., Gomes A. P., Ling A. J. Y. (2012). SIRT1 is required for AMPK activation and the beneficial effects of resveratrol on mitochondrial function. *Cell Metabolism*.

[280] Ou X., Lee M. R., Huang X., Messina-Graham S., Broxmeyer H. E. (2014). SIRT1 positively regulates autophagy and mitochondria function in embryonic stem cells under oxidative stress. *Stem Cells*.

[281] Nasrin N., Kaushik V. K., Fortier E. (2009). JNK1 phosphorylates SIRT1 and promotes its enzymatic activity. *PLoS One*.

[282] Zee R. S., Yoo C. B., Pimentel D. R. (2010). Redox regulation of sirtuin-1 by S-glutathiolation. *Antioxidants & Redox Signaling*.

[283] Zhang Z., Zhang L., Zhou L., Lei Y., Zhang Y., Huang C. (2019). Redox signaling and unfolded protein response coordinate cell fate decisions under ER stress. *Redox Biology*.

[284] Hourihan J. M., Moronetti Mazzeo L. E., Fernández-Cárdenas L. P., Blackwell T. K. (2016). Cysteine sulfenylation directs IRE-1 to activate the SKN-1/Nrf2 antioxidant response. *Molecular Cell*.

[285] Nakato R., Ohkubo Y., Konishi A. (2015). Regulation of the unfolded protein response via S-nitrosylation of sensors of endoplasmic reticulum stress. *Scientific Reports*.

[286] Nadanaka S., Okada T., Yoshida H., Mori K. (2007). Role of disulfide bridges formed in the luminal domain of ATF6 in sensing endoplasmic reticulum stress. *Molecular and Cellular Biology*.

[287] Wilson C., González-Billault C. (2015). Regulation of cytoskeletal dynamics by redox signaling and oxidative stress: implications for neuronal development and trafficking. *Frontiers in Cellular Neuroscience*.

[288] Kauffman K. J., Yu S., Jin J. (2018). Delipidation of mammalian Atg8-family proteins by each of the four ATG4 proteases. *Autophagy*.

[289] Scherz-Shouval R., Shvets E., Fass E., Shorer H., Gil L., Elazar Z. (2007). Reactive oxygen species are essential for autophagy and specifically regulate the activity of Atg4. *The EMBO Journal*.

[290] Qiao S., Dennis M., Song X. (2015). A REDD1/TXNIP pro-oxidant complex regulates ATG4B activity to control stress-induced autophagy and sustain exercise capacity. *Nature Communications*.

[291] Pérez-Pérez M. E., Zaffagnini M., Marchand C. H., Crespo J. L., Lemaire S. D. (2014). The yeast autophagy protease Atg4 is regulated by thioredoxin. *Autophagy*.

[292] Li Y., Zhang Y., Wang L. (2017). Autophagy impairment mediated by S-nitrosation of ATG4B leads to neurotoxicity in response to hyperglycemia. *Autophagy*.

[293] Frudd K., Burgoyne T., Burgoyne J. R. (2018). Oxidation of Atg3 and Atg7 mediates inhibition of autophagy. *Nature Communications*.

[294] Carroll B., Otten E. G., Manni D. (2018). Oxidation of SQSTM1/p62 mediates the link between redox state and protein homeostasis. *Nature Communications*.

[295] Chung K. K., Thomas B., Li X. (2004). S-nitrosylation of parkin regulates ubiquitination and compromises parkin’s protective function. *Science*.

[296] Yao D., Gu Z., Nakamura T. (2004). Nitrosative stress linked to sporadic Parkinson’s disease: S-nitrosylation of parkin regulates its E3 ubiquitin ligase activity. *Proceedings of the National Academy of Sciences of the United States of America*.

[297] Ozawa K., Komatsubara A. T., Nishimura Y. (2013). S-nitrosylation regulates mitochondrial quality control via activation of parkin. *Scientific Reports*.

[298] Meng F., Yao D., Shi Y. (2011). Oxidation of the cysteine-rich regions of parkin perturbs its E3 ligase activity and contributes to protein aggregation. *Molecular Neurodegeneration*.

[299] Tokarew J. M., El-Kodsi D. N., Lengacher N. A. (2020). *Oxidative modifications of parkin underlie its selective neuroprotection in adult human brain*.

[300] Vandiver M. S., Paul B. D., Xu R. (2013). Sulfhydration mediates neuroprotective actions of parkin. *Nature Communications*.

[301] Foroud T., Uniacke S. K., Liu L. (2003). Heterozygosity for a mutation in the parkin gene leads to later onset Parkinson disease. *Neurology*.

[302] Lee S. H., Lee S., du J. (2019). Mitochondrial MsrB2 serves as a switch and transducer for mitophagy. *EMBO Molecular Medicine*.

[303] El Kodsi D. N., Tokarew J. M., Sengupta R. (2020). *Parkinson disease-linked parkin mediates redox reactions that lower oxidative stress in mammalian brain*.

[304] Oh C. K., Sultan A., Platzer J. (2017). S-nitrosylation of PINK1 attenuates PINK1/Parkin-dependent mitophagy in hiPSC-based Parkinson’s disease models. *Cell Reports*.

[305] Gao H., Yang W., Qi Z. (2012). DJ-1 protects dopaminergic neurons against rotenone-induced apoptosis by enhancing ERK-dependent mitophagy. *Journal of Molecular Biology*.

[306] Saito Y. (2014). Oxidized DJ-1 as a possible biomarker of Parkinson’s disease. *Journal of Clinical Biochemistry and Nutrition*.

[307] Canet-Avilés R. M., Wilson M. A., Miller D. W. (2004). The Parkinson’s disease protein DJ-1 is neuroprotective due to cysteine-sulfinic acid-driven mitochondrial localization. *Proceedings of the National Academy of Sciences of the United States of America*.

[308] Zhou W., Zhu M., Wilson M. A., Petsko G. A., Fink A. L. (2006). The oxidation state of DJ-1 regulates its chaperone activity toward alpha-synuclein. *Journal of Molecular Biology*.

[309] Kiss R., Zhu M., Jójárt B. (2017). Structural features of human DJ-1 in distinct Cys106 oxidative states and their relevance to its loss of function in disease. *Biochimica et Biophysica Acta - General Subjects*.

[310] Ariga H., Takahashi-Niki K., Kato I., Maita H., Niki T., Iguchi-Ariga S. M. M. (2013). Neuroprotective function of DJ-1 in Parkinson’s disease. *Oxidative Medicine and Cellular Longevity*.

[311] Ozawa K., Tsumoto H., Miura Y. (2020). DJ-1 is indispensable for the S-nitrosylation of Parkin, which maintains function of mitochondria. *Scientific Reports*.

[312] Di Malta C., Cinque L., Settembre C. (2019). Transcriptional regulation of autophagy: mechanisms and diseases. *Frontiers in Cell and Developmental Biology*.

[313] Settembre C., di Malta C., Polito V. A. (2011). TFEB links autophagy to lysosomal biogenesis. *Science*.

[314] Settembre C., Zoncu R., Medina D. L. (2012). A lysosome-to-nucleus signalling mechanism senses and regulates the lysosome via mTOR and TFEB. *The EMBO Journal*.

[315] Martini-Stoica H., Xu Y., Ballabio A., Zheng H. (2016). The autophagy-lysosomal pathway in neurodegeneration: a TFEB perspective. *Trends in Neurosciences*.

[316] Li S., Song Y., Quach C. (2019). Transcriptional regulation of autophagy-lysosomal function in BRAF-driven melanoma progression and chemoresistance. *Nature Communications*.

[317] Xu Y., Ren J., He X., Chen H., Wei T., Feng W. (2019). YWHA/14-3-3 proteins recognize phosphorylated TFEB by a noncanonical mode for controlling TFEB cytoplasmic localization. *Autophagy*.

[318] Sha Y., Rao L., Settembre C., Ballabio A., Eissa N. T. (2017). STUB1 regulates TFEB-induced autophagy-lysosome pathway. *The EMBO Journal*.

[319] Settembre C., Ballabio A. (2014). TFEB regulates autophagy: an integrated coordination of cellular degradation and recycling processes. *Autophagy*.

[320] Medina D. L., di Paola S., Peluso I. (2015). Lysosomal calcium signalling regulates autophagy through calcineurin and TFEB. *Nature Cell Biology*.

[321] Raben N., Puertollano R. (2016). TFEB and TFE3: linking lysosomes to cellular adaptation to stress. *Annual Review of Cell and Developmental Biology*.

[322] Napolitano G., Esposito A., Choi H. (2018). mTOR-dependent phosphorylation controls TFEB nuclear export. *Nature Communications*.

[323] Dehay B., Martinez-Vicente M., Caldwell G. A. (2013). Lysosomal impairment in Parkinson’s disease. *Movement Disorders*.

[324] Kilpatrick K., Zeng Y., Hancock T., Segatori L. (2015). Genetic and chemical activation of TFEB mediates clearance of aggregated *α*-synuclein. *PLoS One*.

[325] Decressac M., Mattsson B., Weikop P., Lundblad M., Jakobsson J., Bjorklund A. (2013). TFEB-mediated autophagy rescues midbrain dopamine neurons from *α*-synuclein toxicity. *Proceedings of the National Academy of Sciences of the United States of America*.

[326] Tsunemi T., Ashe T. D., Morrison B. E. (2012). PGC-1*α* rescues Huntington’s disease proteotoxicity by preventing oxidative stress and promoting TFEB function. *Science Translational Medicine*.

[327] Polito V. A., Li H., Martini-Stoica H. (2014). Selective clearance of aberrant tau proteins and rescue of neurotoxicity by transcription factor EB. *EMBO Molecular Medicine*.

[328] Wang H., Wang N., Xu D. (2020). Oxidation of multiple MiT/TFE transcription factors links oxidative stress to transcriptional control of autophagy and lysosome biogenesis. *Autophagy*.

[329] Zhao J., Brault J. J., Schild A. (2007). FoxO3 coordinately activates protein degradation by the autophagic/lysosomal and proteasomal pathways in atrophying muscle cells. *Cell Metabolism*.

[330] Yang L., Hu H. M., Zielinska-Kwiatkowska A., Chansky H. A. (2010). FOXO1 is a direct target of EWS-Fli1 oncogenic fusion protein in Ewing’s sarcoma cells. *Biochemical and Biophysical Research Communications*.

[331] Audesse A. J., Dhakal S., Hassell L. A., Gardell Z., Nemtsova Y., Webb A. E. (2019). FOXO3 directly regulates an autophagy network to functionally regulate proteostasis in adult neural stem cells. *PLoS Genetics*.

[332] Klotz L. O., Sánchez-Ramos C., Prieto-Arroyo I., Urbánek P., Steinbrenner H., Monsalve M. (2015). Redox regulation of FoxO transcription factors. *Redox Biology*.

[333] Hay N. (2011). Interplay between FOXO, TOR, and Akt. *Biochimica et Biophysica Acta (BBA) - Molecular Cell Research*.

[334] Zhao Y., Yang J., Liao W. (2010). Cytosolic FoxO1 is essential for the induction of autophagy and tumour suppressor activity. *Nature Cell Biology*.

[335] Cheng Z. (2019). The FoxO-autophagy axis in health and disease. *Trends in Endocrinology and Metabolism*.

[336] Fitzwalter B. E., Towers C. G., Sullivan K. D. (2018). Autophagy inhibition mediates apoptosis sensitization in cancer therapy by relieving FOXO3a turnover. *Developmental Cell*.

[337] Putker M., Madl T., Vos H. R. (2013). Redox-dependent control of FOXO/DAF-16 by transportin-1. *Molecular Cell*.

[338] Hopkins B. L., Nadler M., Skoko J. J. (2018). A peroxidase peroxiredoxin 1-specific redox regulation of the novel FOXO3 microRNA target let-7. *Antioxidants & Redox Signaling*.

[339] Putker M., Vos H. R., van Dorenmalen K. (2015). Evolutionary acquisition of cysteines determines FOXO paralog-specific redox signaling. *Antioxidants & Redox Signaling*.

[340] Gómez-Puerto M. C., Verhagen L. P., Braat A. K., Lam E. W. F., Coffer P. J., Lorenowicz M. J. (2016). Activation of autophagy by FOXO3 regulates redox homeostasis during osteogenic differentiation. *Autophagy*.

[341] Pajares M., Jiménez-Moreno N., García-Yagüe Á. J. (2016). Transcription factor NFE2L2/NRF2 is a regulator of macroautophagy genes. *Autophagy*.

[342] Dinkova-Kostova A. T., Kostov R. V., Canning P. (2017). Keap1, the cysteine-based mammalian intracellular sensor for electrophiles and oxidants. *Archives of Biochemistry and Biophysics*.

[343] Jiang T., Harder B., Rojo de la Vega M., Wong P. K., Chapman E., Zhang D. D. (2015). p62 links autophagy and Nrf2 signaling. *Free Radical Biology & Medicine*.

[344] Park J. Y., Kim S., Sohn H. Y., Koh Y. H., Jo C. (2019). TFEB activates Nrf2 by repressing its E3 ubiquitin ligase DCAF11 and promoting phosphorylation of p62. *Scientific Reports*.

[345] Dodson M., Redmann M., Rajasekaran N. S., Darley-Usmar V., Zhang J. (2015). KEAP1-NRF2 signalling and autophagy in protection against oxidative and reductive proteotoxicity. *The Biochemical Journal*.

[346] Pajares M., Rojo A. I., Arias E., Díaz-Carretero A., Cuervo A. M., Cuadrado A. (2018). Transcription factor NFE2L2/NRF2 modulates chaperone-mediated autophagy through the regulation of LAMP2A. *Autophagy*.

[347] Kasai S., Yamazaki H., Tanji K., Engler M. J., Matsumiya T., Itoh K. (2019). Role of the ISR-ATF4 pathway and its cross talk with Nrf2 in mitochondrial quality control. *Journal of Clinical Biochemistry and Nutrition*.

[348] Lacher S. E., Levings D. C., Freeman S., Slattery M. (2018). Identification of a functional antioxidant response element at the HIF1A locus. *Redox Biology*.

[349] Pike L. R. G., Singleton D. C., Buffa F. (2013). Transcriptional up-regulation of ULK1 by ATF4 contributes to cancer cell survival. *The Biochemical Journal*.

[350] Zhang H., Bosch-Marce M., Shimoda L. A. (2008). Mitochondrial autophagy is an HIF-1-dependent adaptive metabolic response to hypoxia. *The Journal of Biological Chemistry*.

[351] Li F., Sonveaux P., Rabbani Z. N. (2007). Regulation of HIF-1alpha stability through S-nitrosylation. *Molecular Cell*.

[352] Wang Y., Yang J., Yang K. (2012). The biphasic redox sensing of SENP3 accounts for the HIF-1 transcriptional activity shift by oxidative stress. *Acta Pharmacologica Sinica*.

[353] Lange P. S., Chavez J. C., Pinto J. T. (2008). ATF4 is an oxidative stress-inducible, prodeath transcription factor in neurons in vitro and in vivo. *The Journal of Experimental Medicine*.

[354] Li H. S., Zhou Y. N., Li L. (2019). HIF-1*α* protects against oxidative stress by directly targeting mitochondria. *Redox Biology*.

[355] Kenzelmann Broz D., Spano Mello S., Bieging K. T. (2013). Global genomic profiling reveals an extensive p53-regulated autophagy program contributing to key p53 responses. *Genes & Development*.

[356] Brady O. A., Jeong E., Martina J. A., Pirooznia M., Tunc I., Puertollano R. (2018). The transcription factors TFE3 and TFEB amplify p53 dependent transcriptional programs in response to DNA damage. *eLife*.

[357] Bae S. H., Sung S. H., Oh S. Y. (2013). Sestrins activate Nrf2 by promoting p62-dependent autophagic degradation of Keap1 and prevent oxidative liver damage. *Cell Metabolism*.

[358] Scherz-Shouval R., Weidberg H., Gonen C., Wilder S., Elazar Z., Oren M. (2010). p53-dependent regulation of autophagy protein LC3 supports cancer cell survival under prolonged starvation. *Proceedings of the National Academy of Sciences of the United States of America*.

[359] Velu C. S., Niture S. K., Doneanu C. E., Pattabiraman N., Srivenugopal K. S. (2007). Human p53 is inhibited by glutathionylation of cysteines present in the proximal DNA-binding domain during oxidative stress. *Biochemistry*.

[360] Domenico F. D., Cenini G., Sultana R. (2009). Glutathionylation of the pro-apoptotic protein p53 in Alzheimer’s disease brain: implications for AD pathogenesis. *Neurochemical Research*.

[361] Baldelli S., Ciriolo M. R. (2016). Altered S-nitrosylation of p53 is responsible for impaired antioxidant response in skeletal muscle during aging. *Aging*.

[362] Lingappan K. (2018). NF-*κ*B in oxidative stress. *Current Opinion in Toxicology*.

[363] Yamamoto Y., Gaynor R. B. (2004). IkappaB kinases: key regulators of the NF-kappaB pathway. *Trends in Biochemical Sciences*.

[364] Morgan M. J., Liu Z. G. (2011). Crosstalk of reactive oxygen species and NF-*κ*B signaling. *Cell Research*.

[365] Zhang Z., Guo M., Zhao S. (2015). The update on transcriptional regulation of autophagy in normal and pathologic cells: a novel therapeutic target. *Biomedicine & Pharmacotherapy*.

[366] Yogev O., Goldberg R., Anzi S., Yogev O., Shaulian E. (2010). Jun proteins are starvation-regulated inhibitors of autophagy. *Cancer Research*.

[367] Moreau K., Ghislat G., Hochfeld W. (2015). Transcriptional regulation of Annexin A2 promotes starvation-induced autophagy. *Nature Communications*.

[368] Matthews J. R., Wakasugi N., virelizier J. L., Yodoi J., Hay R. T. (1992). Thioredoxin regulates the DNA binding activity of NF-kappa B by reduction of a disulphide bond involving cysteine 62. *Nucleic Acids Research*.

[369] Pineda-Molina E., Klatt P., Vázquez J. (2001). Glutathionylation of the p50 subunit of NF-kappaB: a mechanism for redox-induced inhibition of DNA binding. *Biochemistry*.

[370] Matthews J. R., Botting C. H., Panico M., Morris H. R., Hay R. T. (1996). Inhibition of NF-kappaB DNA binding by nitric oxide. *Nucleic Acids Research*.

[371] Korbecki J., Bobinski R., Dutka M. (2019). Self-regulation of the inflammatory response by peroxisome proliferator-activated receptors. *Inflammation Research*.

[372] Yin R., Fang L., Li Y. (2015). Pro-inflammatory macrophages suppress PPAR*γ* activity in adipocytes via S-nitrosylation. *Free Radical Biology & Medicine*.

[373] Wang Y. D., Chen W. D., Moore D. D., Huang W. (2008). FXR: a metabolic regulator and cell protector. *Cell Research*.

[374] Wang Y. D., Chen W. D., Li C. (2015). Farnesoid X receptor antagonizes JNK signaling pathway in liver carcinogenesis by activating SOD3. *Molecular Endocrinology*.

[375] Wang B., Zhang H., Luan Z. (2020). Farnesoid X receptor (FXR) activation induces the antioxidant protein metallothionein 1 expression in mouse liver. *Experimental Cell Research*.

[376] Seok S., Fu T., Choi S. E. (2014). Transcriptional regulation of autophagy by an FXR-CREB axis. *Nature*.

[377] Hirsch L., Jette N., Frolkis A., Steeves T., Pringsheim T. (2016). The incidence of Parkinson’s disease: a systematic review and meta-analysis. *Neuroepidemiology*.

[378] Mhyre T. R., Boyd J. T., Hamill R. W., Maguire-Zeiss K. A. (2012). Parkinson’s disease. *Sub-Cellular Biochemistry*.

[379] Ishihara L. S., Cheesbrough A., Brayne C., Schrag A. (2007). Estimated life expectancy of Parkinson’s patients compared with the UK population. *Journal of Neurology, Neurosurgery, and Psychiatry*.

[380] Bäckström D., Granåsen G., Domellöf M. E. (2018). Early predictors of mortality in parkinsonism and Parkinson disease: a population-based study. *Neurology*.

[381] Caggiu E., Arru G., Hosseini S. (2019). Inflammation, infectious triggers, and Parkinson’s disease. *Frontiers in Neurology*.

[382] Feng D. D., Cai W., Chen X. (2015). The associations between Parkinson’s disease and cancer: the plot thickens. *Translational Neurodegeneration*.

[383] Hong C. T., Hu H. H., Chan L., Bai C. H. (2018). Prevalent cerebrovascular and cardiovascular disease in people with Parkinson’s disease: a meta-analysis. *Clinical Epidemiology*.

[384] *CS2960 Incidence and Prevalence Report Branding Summary Report; Parkinson’s Disease Society of the United Kingdom*.

[385] Ball N., Teo W. P., Chandra S., Chapman J. (2019). Parkinson’s disease and the environment. *Frontiers in Neurology*.

[386] Verstraeten A., Theuns J., Van Broeckhoven C. (2015). Progress in unraveling the genetic etiology of Parkinson disease in a genomic era. *Trends in Genetics*.

[387] Del Rey N. L. G., Quiroga-Varela A., Garbayo E. (2018). Advances in Parkinson’s disease: 200 years later. *Frontiers in Neuroanatomy*.

[388] Mazzoni P., Shabbott B., Cortes J. C. (2012). Motor control abnormalities in Parkinson’s disease. *Cold Spring Harbor Perspectives in Medicine*.

[389] Roheger M., Kalbe E., Liepelt-Scarfone I. (2018). Progression of cognitive decline in Parkinson’s disease. *Journal of Parkinson’s Disease*.

[390] Scullin M. K., Sollinger A. B., Land J. (2013). Sleep and impulsivity in Parkinson’s disease. *Parkinsonism & Related Disorders*.

[391] Brichta L., Greengard P., Flajolet M. (2013). Advances in the pharmacological treatment of Parkinson’s disease: targeting neurotransmitter systems. *Trends in Neurosciences*.

[392] Maiti P., Manna J., Dunbar G. L. (2017). Current understanding of the molecular mechanisms in Parkinson’s disease: targets for potential treatments. *Translational Neurodegeneration*.

[393] Kett L. R., Stiller B., Bernath M. M. (2015). *α*-Synuclein-independent histopathological and motor deficits in mice lacking the endolysosomal Parkinsonism protein Atp13a2. *The Journal of Neuroscience*.

[394] Friedman L. G., Lachenmayer M. L., Wang J. (2012). Disrupted autophagy leads to dopaminergic axon and dendrite degeneration and promotes presynaptic accumulation of *α*-synuclein and LRRK2 in the brain. *The Journal of Neuroscience*.

[395] Guo J. D., Zhao X., Li Y., Li G. R., Liu X. L. (2018). Damage to dopaminergic neurons by oxidative stress in Parkinson’s disease (review). *International Journal of Molecular Medicine*.

[396] Trist B. G., Hare D. J., Double K. L. (2019). Oxidative stress in the aging substantia nigra and the etiology of Parkinson’s disease. *Aging Cell*.

[397] Chinta S. J., Andersen J. K. (2005). Dopaminergic neurons. *The International Journal of Biochemistry & Cell Biology*.

[398] Purves D. (2012). *Neuroscience*.

[399] Haavik J., Almas B., Flatmark T. (1997). Generation of reactive oxygen species by tyrosine hydroxylase: a possible contribution to the degeneration of dopaminergic neurons?. *Journal of Neurochemistry*.

[400] Meiser J., Weindl D., Hiller K. (2013). Complexity of dopamine metabolism. *Cell Communication and Signaling: CCS*.

[401] Mishra A., Singh S., Shukla S. (2018). Physiological and functional basis of dopamine receptors and their role in neurogenesis: possible implication for Parkinson’s disease. *Journal of Experimental Neuroscience*.

[402] Vaughan R. A., Foster J. D. (2013). Mechanisms of dopamine transporter regulation in normal and disease states. *Trends in Pharmacological Sciences*.

[403] Luo S. X., Huang E. J. (2016). Dopaminergic neurons and brain reward pathways: from neurogenesis to circuit assembly. *The American Journal of Pathology*.

[404] Sgroi S., Tonini R. (2018). Opioidergic modulation of striatal circuits, implications in Parkinson’s disease and levodopa induced dyskinesia. *Frontiers in Neurology*.

[405] Pretegiani E., Optican L. M. (2017). Eye movements in Parkinson’s disease and inherited parkinsonian syndromes. *Frontiers in Neurology*.

[406] Galvan A., Devergnas A., Wichmann T. (2015). Alterations in neuronal activity in basal ganglia-thalamocortical circuits in the parkinsonian state. *Frontiers in Neuroanatomy*.

[407] Dragicevic E., Schiemann J., Liss B. (2015). Dopamine midbrain neurons in health and Parkinson’s disease: emerging roles of voltage-gated calcium channels and ATP-sensitive potassium channels. *Neuroscience*.

[408] Tagliaferro P., Burke R. E. (2016). Retrograde axonal degeneration in Parkinson disease. *Journal of Parkinson’s Disease*.

[409] Paladini C. A., Robinson S., Morikawa H., Williams J. T., Palmiter R. D. (2003). Dopamine controls the firing pattern of dopamine neurons via a network feedback mechanism. *Proceedings of the National Academy of Sciences of the United States of America*.

[410] Bolam J. P., Pissadaki E. K. (2012). Living on the edge with too many mouths to feed: why dopamine neurons die. *Movement Disorders*.

[411] Grace A. A., Bunney B. S. (1984). The control of firing pattern in nigral dopamine neurons: single spike firing. *The Journal of Neuroscience*.

[412] Surmeier D. J., Obeso J. A., Halliday G. M. (2017). Selective neuronal vulnerability in Parkinson disease. *Nature Reviews Neuroscience*.

[413] Foehring R. C., Zhang X. F., Lee J. C. F., Callaway J. C. (2009). Endogenous calcium buffering capacity of substantia nigral dopamine neurons. *Journal of Neurophysiology*.

[414] Guzman J. N., Sánchez-Padilla J., Chan C. S., Surmeier D. J. (2009). Robust pacemaking in substantia nigra dopaminergic neurons. *The Journal of Neuroscience*.

[415] Philippart F., Destreel G., Merino-Sepulveda P., Henny P., Engel D., Seutin V. (2016). Differential somatic Ca^2+^ channel profile in midbrain dopaminergic neurons. *The Journal of Neuroscience*.

[416] Denton R. M., McCormack J. G. (1980). The role of calcium in the regulation of mitochondrial metabolism. *Biochemical Society Transactions*.

[417] Pacelli C., Giguère N., Bourque M. J., Lévesque M., Slack R. S., Trudeau L. É. (2015). Elevated mitochondrial bioenergetics and axonal arborization size are key contributors to the vulnerability of dopamine neurons. *Current Biology*.

[418] Lan J., Jiang D. H. (1997). Desferrioxamine and vitamin E protect against iron and MPTP-induced neurodegeneration in mice. *Journal of Neural Transmission*.

[419] Sian-Hülsmann J., Mandel S., Youdim M. B. H., Riederer P. (2011). The relevance of iron in the pathogenesis of Parkinson’s disease. *Journal of Neurochemistry*.

[420] Martin W. R. W., Wieler M., Gee M. (2008). Midbrain iron content in early Parkinson disease: a potential biomarker of disease status. *Neurology*.

[421] Nuñez M. T., Chana-Cuevas P. (2018). New perspectives in iron chelation therapy for the treatment of neurodegenerative diseases. *Pharmaceuticals*.

[422] Hunn B. H. M., Cragg S. J., Bolam J. P., Spillantini M. G., Wade-Martins R. (2015). Impaired intracellular trafficking defines early Parkinson’s disease. *Trends in Neurosciences*.

[423] William Langston J., Forno L. S., Rebert C. S., Irwin I. (1984). Selective nigral toxicity after systemic administration of 1-methyl-4-phenyl-1,2,5,6-tetrahydropyrine (MPTP) in the squirrel monkey. *Brain Research*.

[424] Zeng X. S., Geng W. S., Jia J. J. (2018). Neurotoxin-induced animal models of Parkinson disease: pathogenic mechanism and assessment. *ASN Neuro*.

[425] Ren Y., Liu W., Jiang H., Jiang Q., Feng J. (2005). Selective vulnerability of dopaminergic neurons to microtubule depolymerization. *The Journal of Biological Chemistry*.

[426] Ahmadi F. A., Linseman D. A., Grammatopoulos T. N. (2003). The pesticide rotenone induces caspase-3-mediated apoptosis in ventral mesencephalic dopaminergic neurons. *Journal of Neurochemistry*.

[427] Ahmadi F. A., Grammatopoulos T. N., Poczobutt A. M. (2008). Dopamine selectively sensitizes dopaminergic neurons to rotenone-induced apoptosis. *Neurochemical Research*.

[428] Alam M., Schmidt W. J. (2002). Rotenone destroys dopaminergic neurons and induces parkinsonian symptoms in rats. *Behavioural Brain Research*.

[429] Betarbet R., Sherer T. B., MacKenzie G., Garcia-Osuna M., Panov A. V., Greenamyre J. T. (2000). Chronic systemic pesticide exposure reproduces features of Parkinson’s disease. *Nature Neuroscience*.

[430] Tabata Y., Imaizumi Y., Sugawara M. (2018). T-type calcium channels determine the vulnerability of dopaminergic neurons to mitochondrial stress in familial Parkinson disease. *Stem Cell Reports*.

[431] Worth A. J., Basu S. S., Snyder N. W., Mesaros C., Blair I. A. (2014). Inhibition of neuronal cell mitochondrial complex I with rotenone increases lipid *β*-oxidation, supporting acetyl-coenzyme A levels. *The Journal of Biological Chemistry*.

[432] Terron A., Bal-Price A., Paini A. (2018). An adverse outcome pathway for parkinsonian motor deficits associated with mitochondrial complex I inhibition. *Archives of Toxicology*.

[433] Wu F., Xu H. D., Guan J. J. (2015). Rotenone impairs autophagic flux and lysosomal functions in Parkinson’s disease. *Neuroscience*.

[434] Kim J., Shin S. D., Jeong S., Suh G. J., Kwak Y. H. (2017). Effect of prohibiting the use of Paraquat on pesticide-associated mortality. *BMC Public Health*.

[435] Jiménez-Jiménez F., Alonso-Navarro H., Herrero M., García-Martín E., Agúndez J. (2016). An update on the role of nitric oxide in the neurodegenerative processes of Parkinson’s disease. *Current Medicinal Chemistry*.

[436] Liu Z., Jing Y., Yin J., Mu J., Yao T., Gao L. (2013). Downregulation of thioredoxin reductase 1 expression in the substantia nigra pars compacta of Parkinson’s disease mice. *Neural Regeneration Research*.

[437] Venkateshappa C., Harish G., Mythri R. B., Mahadevan A., Srinivas Bharath M. M., Shankar S. K. (2012). Increased oxidative damage and decreased antioxidant function in aging human substantia nigra compared to striatum: implications for Parkinson’s disease. *Neurochemical Research*.

[438] Filograna R., Beltramini M., Bubacco L., Bisaglia M. (2016). Anti-oxidants in Parkinson’s disease therapy: a critical point of view. *Current Neuropharmacology*.

[439] Lastres-Becker I., Ulusoy A., Innamorato N. G. (2012). *α*-Synuclein expression and Nrf2 deficiency cooperate to aggravate protein aggregation, neuronal death and inflammation in early-stage Parkinson’s disease. *Human Molecular Genetics*.

[440] Jazwa A., Rojo A. I., Innamorato N. G., Hesse M., Fernández-Ruiz J., Cuadrado A. (2011). Pharmacological targeting of the transcription factor Nrf2 at the basal ganglia provides disease modifying therapy for experimental parkinsonism. *Antioxidants & Redox Signaling*.

[441] Lastres-Becker I., García-Yagüe A. J., Scannevin R. H. (2016). Repurposing the NRF2 activator dimethyl fumarate as therapy against synucleinopathy in Parkinson’s disease. *Antioxidants & Redox Signaling*.

[442] Barone M. C., Sykiotis G. P., Bohmann D. (2011). Genetic activation of Nrf2 signaling is sufficient to ameliorate neurodegenerative phenotypes in a *Drosophila* model of Parkinson’s disease. *Disease Models & Mechanisms*.

[443] Belarbi K., Cuvelier E., Destée A., Gressier B., Chartier-Harlin M. C. (2017). NADPH oxidases in Parkinson’s disease: a systematic review. *Molecular Neurodegeneration*.

[444] Pal R., Bajaj L., Sharma J. (2016). NADPH oxidase promotes Parkinsonian phenotypes by impairing autophagic flux in an mTORC1-independent fashion in a cellular model of Parkinson’s disease. *Scientific Reports*.

[445] Barua S., Kim J. Y., Yenari M. A., Lee J. E. (2019). The role of NOX inhibitors in neurodegenerative diseases. *IBRO Reports*.

[446] Segura-Aguilar J., Paris I., Muñoz P., Ferrari E., Zecca L., Zucca F. A. (2014). Protective and toxic roles of dopamine in Parkinson’s disease. *Journal of Neurochemistry*.

[447] Zecca L., Casella L., Albertini A. (2008). Neuromelanin can protect against iron-mediated oxidative damage in system modeling iron overload of brain aging and Parkinson’s disease. *Journal of Neurochemistry*.

[448] Norris E. H., Giasson B. I., Hodara R. (2005). Reversible inhibition of alpha-synuclein fibrillization by dopaminochrome-mediated conformational alterations. *The Journal of Biological Chemistry*.

[449] Van Laar V. S., Mishizen A. J., Cascio M., Hastings T. G. (2009). Proteomic identification of dopamine-conjugated proteins from isolated rat brain mitochondria and SH-SY5Y cells. *Neurobiology of Disease*.

[450] Paris I., Perez-Pastene C., Cardenas S. (2010). Aminochrome induces disruption of actin, alpha-, and beta-tubulin cytoskeleton networks in substantia-nigra-derived cell line. *Neurotoxicity Research*.

[451] Burbulla L. F., Song P., Mazzulli J. R. (2017). Dopamine oxidation mediates mitochondrial and lysosomal dysfunction in Parkinson’s disease. *Science*.

[452] Santos C. C., Araújo F. M., Ferreira R. S. (2017). Aminochrome induces microglia and astrocyte activation. *Toxicology In Vitro*.

[453] Muñoz P., Huenchuguala S., Paris I., Segura-Aguilar J. (2012). Dopamine oxidation and autophagy. *Parkinson’s Disease*.

[454] Jin M. M., Wang F., Qi D. (2018). A critical role of autophagy in regulating microglia polarization in neurodegeneration. *Frontiers in Aging Neuroscience*.

[455] Pellegrini L., Wetzel A., Grannó S., Heaton G., Harvey K. (2017). Back to the tubule: microtubule dynamics in Parkinson’s disease. *Cellular and Molecular Life Sciences*.

[456] Lee S., Sato Y., Nixon R. A. (2011). Lysosomal proteolysis inhibition selectively disrupts axonal transport of degradative organelles and causes an Alzheimer’s-like axonal dystrophy. *The Journal of Neuroscience*.

[457] Cristofani R., Crippa V., Rusmini P. (2017). Inhibition of retrograde transport modulates misfolded protein accumulation and clearance in motoneuron diseases. *Autophagy*.

[458] Spencer B., Potkar R., Trejo M. (2009). Beclin 1 gene transfer activates autophagy and ameliorates the neurodegenerative pathology in alpha-synuclein models of Parkinson’s and Lewy body diseases. *The Journal of Neuroscience*.

[459] Xilouri M., Brekk O. R., Landeck N. (2013). Boosting chaperone-mediated autophagy in vivo mitigates *α*-synuclein-induced neurodegeneration. *Brain*.

[460] Gao J., Perera G., Bhadbhade M., Halliday G. M., Dzamko N. (2019). Autophagy activation promotes clearance of *α*-synuclein inclusions in fibril-seeded human neural cells. *The Journal of Biological Chemistry*.

[461] Hunn B. H. M., Vingill S., Threlfell S. (2019). Impairment of macroautophagy in dopamine neurons has opposing effects on Parkinsonian pathology and behavior. *Cell Reports*.

[462] Dagda R. K., Cherra S. J., Kulich S. M., Tandon A., Park D., Chu C. T. (2009). Loss of PINK1 function promotes mitophagy through effects on oxidative stress and mitochondrial fission. *The Journal of Biological Chemistry*.

[463] Evans C. S., Holzbaur E. L. F. (2020). Quality control in neurons: mitophagy and other selective autophagy mechanisms. *Journal of Molecular Biology*.

[464] Pagan F., Hebron M., Valadez E. H. (2016). Nilotinib effects in Parkinson’s disease and dementia with Lewy bodies. *Journal of Parkinson’s Disease*.

[465] Pagan F. L., Hebron M. L., Wilmarth B. (2020). Nilotinib effects on safety, tolerability, and potential biomarkers in Parkinson disease: a phase 2 randomized clinical trial. *JAMA Neurology*.

[466] Thayer J. A., Awad O., Hegdekar N. (2020). The PARK10 gene USP24 is a negative regulator of autophagy and ULK1 protein stability. *Autophagy*.

[467] Yasuda T., Nihira T., Ren Y. R. (2009). Effects of UCH-L1 on alpha-synuclein over-expression mouse model of Parkinson’s disease. *Journal of Neurochemistry*.

[468] Yan C., Huo H., Yang C., Zhang T., Chu Y., Liu Y. (2018). Ubiquitin C-terminal hydrolase L1 regulates autophagy by inhibiting autophagosome formation through its deubiquitinating enzyme activity. *Biochemical and Biophysical Research Communications*.

[469] Kumar R., Jangir D. K., Verma G. (2017). S-nitrosylation of UCHL1 induces its structural instability and promotes *α*-synuclein aggregation. *Scientific Reports*.

[470] Shen H., Sikorska M., LeBlanc J., Walker P. R., Liu Q. Y. (2006). Oxidative stress regulated expression of ubiquitin carboxyl-terminal hydrolase-L1: role in cell survival. *Apoptosis*.

[471] Thomas K. J., McCoy M. K., Blackinton J. (2011). DJ-1 acts in parallel to the PINK1/parkin pathway to control mitochondrial function and autophagy. *Human Molecular Genetics*.

[472] Dolgacheva L. P., Berezhnov A. V., Fedotova E. I., Zinchenko V. P., Abramov A. Y. (2019). Role of DJ-1 in the mechanism of pathogenesis of Parkinson’s disease. *Journal of Bioenergetics and Biomembranes*.

[473] Piston D., Alvarez-Erviti L., Bansal V. (2018). DJ-1 is a redox sensitive adapter protein for high molecular weight complexes involved in regulation of catecholamine homeostasis. *Human Molecular Genetics*.

[474] Inden M., Kitamura Y., Takahashi K. (2011). Protection against dopaminergic neurodegeneration in Parkinson’s disease-model animals by a modulator of the oxidized form of DJ-1, a wild-type of familial Parkinson’s disease-linked PARK7. *Journal of Pharmacological Sciences*.

[475] Orenstein S. J., Kuo S. H., Tasset I. (2013). Interplay of LRRK2 with chaperone-mediated autophagy. *Nature Neuroscience*.

[476] Cookson M. R. (2015). LRRK2 pathways leading to neurodegeneration. *Current Neurology and Neuroscience Reports*.

[477] Kelly K., Wang S., Boddu R. (2018). The G2019S mutation in LRRK2 imparts resiliency to kinase inhibition. *Experimental Neurology*.

[478] Sweet E. S., Saunier-Rebori B., Yue Z., Blitzer R. D. (2015). The Parkinson’s disease-associated mutation LRRK2-G2019S impairs synaptic plasticity in mouse hippocampus. *The Journal of Neuroscience*.

[479] Atashrazm F., Dzamko N. (2016). LRRK2 inhibitors and their potential in the treatment of Parkinson’s disease: current perspectives. *Clinical Pharmacology: Advances and Applications*.

[480] Bonello F., Hassoun S. M., Mouton-Liger F. (2019). LRRK2 impairs PINK1/Parkin-dependent mitophagy via its kinase activity: pathologic insights into Parkinson’s disease. *Human Molecular Genetics*.

[481] Wauters F., Cornelissen T., Imberechts D. (2020). LRRK2 mutations impair depolarization-induced mitophagy through inhibition of mitochondrial accumulation of RAB10. *Autophagy*.

[482] Albanese F., Novello S., Morari M. (2019). Autophagy and LRRK2 in the aging brain. *Frontiers in Neuroscience*.

[483] Wallings R., Connor-Robson N., Wade-Martins R. (2019). LRRK2 interacts with the vacuolar-type H+-ATPase pump a1 subunit to regulate lysosomal function. *Human Molecular Genetics*.

[484] Pereira C., Miguel Martins L., Saraiva L. (2014). LRRK2, but not pathogenic mutants, protects against H_2_O_2_ stress depending on mitochondrial function and endocytosis in a yeast model. *Biochimica et Biophysica Acta (BBA) - General Subjects*.

[485] Nguyen M., Krainc D. (2018). LRRK2 phosphorylation of auxilin mediates synaptic defects in dopaminergic neurons from patients with Parkinson’s disease. *Proceedings of the National Academy of Sciences of the United States of America*.

[486] Mamais A., Chia R., Beilina A. (2014). Arsenite stress down-regulates phosphorylation and 14-3-3 binding of leucine-rich repeat kinase 2 (LRRK2), promoting self-association and cellular redistribution. *The Journal of Biological Chemistry*.

[487] Ysselstein D., Nguyen M., Young T. J. (2019). LRRK2 kinase activity regulates lysosomal glucocerebrosidase in neurons derived from Parkinson’s disease patients. *Nature Communications*.

[488] Schöndorf D. C., Aureli M., McAllister F. E. (2014). iPSC-derived neurons from GBA1-associated Parkinson’s disease patients show autophagic defects and impaired calcium homeostasis. *Nature Communications*.

[489] Gegg M. E., Schapira A. H. V. (2016). Mitochondrial dysfunction associated with glucocerebrosidase deficiency. *Neurobiology of Disease*.

[490] Li H., Ham A., Ma T. C. (2019). Mitochondrial dysfunction and mitophagy defect triggered by heterozygous GBA mutations. *Autophagy*.

[491] Salmina A. B. (2009). Neuron-glia interactions as therapeutic targets in neurodegeneration. *Journal of Alzheimer’s Disease*.

[492] Lian H., Zheng H. (2016). Signaling pathways regulating neuron-glia interaction and their implications in Alzheimer’s disease. *Journal of Neurochemistry*.

[493] Booth H. D. E., Hirst W. D., Wade-Martins R. (2017). The role of astrocyte dysfunction in Parkinson’s disease pathogenesis. *Trends in Neurosciences*.

[494] Madill M., McDonagh K., Ma J. (2017). Amyotrophic lateral sclerosis patient iPSC-derived astrocytes impair autophagy via non-cell autonomous mechanisms. *Molecular Brain*.

[495] Gan L., Vargas M. R., Johnson D. A., Johnson J. A. (2012). Astrocyte-specific overexpression of Nrf2 delays motor pathology and synuclein aggregation throughout the CNS in the alpha-synuclein mutant (A53T) mouse model. *The Journal of Neuroscience*.

[496] di Domenico A., Carola G., Calatayud C. (2019). Patient-specific iPSC-derived astrocytes contribute to non-cell-autonomous neurodegeneration in Parkinson’s disease. *Stem Cell Reports*.

[497] Davis C. O., Kim K. Y., Bushong E. A. (2014). Transcellular degradation of axonal mitochondria. *Proceedings of the National Academy of Sciences of the United States of America*.

[498] Melentijevic I., Toth M. L., Arnold M. L. (2017). *C. elegans* neurons jettison protein aggregates and mitochondria under neurotoxic stress. *Nature*.

[499] Markesbery W. R., Jicha G. A., Liu H., Schmitt F. A. (2009). Lewy body pathology in normal elderly subjects. *Journal of Neuropathology and Experimental Neurology*.

[500] Wakabayashi K., Tanji K., Mori F., Takahashi H. (2007). The Lewy body in Parkinson’s disease: molecules implicated in the formation and degradation of alpha-synuclein aggregates. *Neuropathology*.

[501] Zhang G., Xia Y., Wan F. (2018). New perspectives on roles of alpha-synuclein in Parkinson’s disease. *Frontiers in Aging Neuroscience*.

[502] Meade R. M., Fairlie D. P., Mason J. M. (2019). Alpha-synuclein structure and Parkinson’s disease—lessons and emerging principles. *Molecular Neurodegeneration*.

[503] Giasson B. I., Duda J. E., Murray I. V. (2000). Oxidative damage linked to neurodegeneration by selective alpha-synuclein nitration in synucleinopathy lesions. *Science*.

[504] Jiang C., Chang J. Y. (2007). Isomers of human alpha-synuclein stabilized by disulfide bonds exhibit distinct structural and aggregative properties. *Biochemistry*.

[505] Ponzini E., de Palma A., Cerboni L. (2019). Methionine oxidation in *α*-synuclein inhibits its propensity for ordered secondary structure. *The Journal of Biological Chemistry*.

[506] Calì T., Ottolini D., Negro A., Brini M. (2012). *α*-Synuclein controls mitochondrial calcium homeostasis by enhancing endoplasmic reticulum-mitochondria interactions. *The Journal of Biological Chemistry*.

[507] Gómez-Suaga P., Bravo-San Pedro J. M., González-Polo R. A., Fuentes J. M., Niso-Santano M. (2018). ER-mitochondria signaling in Parkinson’s disease. *Cell Death & Disease*.

[508] Guardia-Laguarta C., Area-Gomez E., Schon E. A., Przedborski S. (2015). Novel subcellular localization for alpha-synuclein: possible functional consequences. *Frontiers in Neuroanatomy*.

[509] Paillusson S., Gomez-Suaga P., Stoica R. (2017). *α*-Synuclein binds to the ER-mitochondria tethering protein VAPB to disrupt Ca^2+^ homeostasis and mitochondrial ATP production. *Acta Neuropathologica*.

[510] Lashuel H. A., Overk C. R., Oueslati A., Masliah E. (2013). The many faces of *α*-synuclein: from structure and toxicity to therapeutic target. *Nature Reviews Neuroscience*.

[511] Zhang W., Wang T., Pei Z. (2005). Aggregated alpha-synuclein activates microglia: a process leading to disease progression in Parkinson’s disease. *The FASEB Journal*.

[512] Valdinocci D., Simões R. F., Kovarova J., Cunha-Oliveira T., Neuzil J., Pountney D. L. (2019). Intracellular and intercellular mitochondrial dynamics in Parkinson’s disease. *Frontiers in Neuroscience*.

[513] Martinez-Vicente M., Talloczy Z., Kaushik S. (2008). Dopamine-modified alpha-synuclein blocks chaperone-mediated autophagy. *The Journal of Clinical Investigation*.

[514] Miraglia F., Ricci A., Rota L., Colla E. (2018). Subcellular localization of alpha-synuclein aggregates and their interaction with membranes. *Neural Regeneration Research*.

[515] Lei Z., Cao G., Wei G. (2019). A30P mutant *α*-synuclein impairs autophagic flux by inactivating JNK signaling to enhance ZKSCAN3 activity in midbrain dopaminergic neurons. *Cell Death & Disease*.

[516] Wilkaniec A., Lenkiewicz A. M., Czapski G. A. (2019). Extracellular alpha-synuclein oligomers induce parkin S-nitrosylation: relevance to sporadic Parkinson’s disease etiopathology. *Molecular Neurobiology*.

[517] Kumar R., Kumari R., Kumar S., Jangir D. K., Maiti T. K. (2018). Extracellular *α*-synuclein disrupts membrane nanostructure and promotes S-nitrosylation-induced neuronal cell death. *Biomacromolecules*.

[518] Colla E. (2019). Linking the endoplasmic reticulum to Parkinson’s disease and alpha-synucleinopathy. *Frontiers in Neuroscience*.

[519] Winslow A. R., Chen C. W., Corrochano S. (2010). *α*-Synuclein impairs macroautophagy: implications for Parkinson’s disease. *The Journal of Cell Biology*.

[520] Tanik S. A., Schultheiss C. E., Volpicelli-Daley L. A., Brunden K. R., Lee V. M. Y. (2013). Lewy body-like *α*-synuclein aggregates resist degradation and impair macroautophagy. *The Journal of Biological Chemistry*.

[521] Recasens A., Dehay B. (2014). Alpha-synuclein spreading in Parkinson’s disease. *Frontiers in Neuroanatomy*.

[522] Lee H. J., Cho E. D., Lee K. W., Kim J. H., Cho S. G., Lee S. J. (2013). Autophagic failure promotes the exocytosis and intercellular transfer of *α*-synuclein. *Experimental & Molecular Medicine*.

[523] Musgrove R. E., Helwig M., Bae E. J. (2019). Oxidative stress in vagal neurons promotes parkinsonian pathology and intercellular *α*-synuclein transfer. *The Journal of Clinical Investigation*.

[524] Gelders G., Baekelandt V., Van der Perren A. (2018). Linking neuroinflammation and neurodegeneration in Parkinson’s disease. *Journal of Immunology Research*.

[525] Taylor J. M., Main B. S., Crack P. J. (2013). Neuroinflammation and oxidative stress: co-conspirators in the pathology of Parkinson’s disease. *Neurochemistry International*.

[526] Rizor A., Pajarillo E., Johnson J., Aschner M., Lee E. (2019). Astrocytic oxidative/nitrosative stress contributes to Parkinson’s disease pathogenesis: the dual role of reactive astrocytes. *Antioxidants*.

[527] Peterson L. J., Flood P. M. (2012). Oxidative stress and microglial cells in Parkinson’s disease. *Mediators of Inflammation*.

[528] Du D., Hu L., Wu J. (2017). Neuroinflammation contributes to autophagy flux blockage in the neurons of rostral ventrolateral medulla in stress-induced hypertension rats. *Journal of Neuroinflammation*.

[529] Cheng J., Liao Y., Xiao L. (2017). Autophagy regulates MAVS signaling activation in a phosphorylation-dependent manner in microglia. *Cell Death and Differentiation*.

[530] Sun Q., Fan J., Billiar T. R., Scott M. J. (2017). Inflammasome and autophagy regulation—a two-way street. *Molecular Medicine*.

[531] Liu T. (2019). Regulation of inflammasome by autophagy. *Advances in Experimental Medicine and Biology*.

[532] Cao Z., Wang Y., Long Z., He G. (2019). Interaction between autophagy and the NLRP3 inflammasome. *Acta Biochimica et Biophysica Sinica*.

[533] Cheng J., Liao Y., Dong Y. (2020). Microglial autophagy defect causes parkinson disease-like symptoms by accelerating inflammasome activation in mice. *Autophagy*.

[534] Su P., Zhang J., Wang D. (2016). The role of autophagy in modulation of neuroinflammation in microglia. *Neuroscience*.

[535] Sung K., Jimenez-Sanchez M. (2020). Autophagy in astrocytes and its implications in neurodegeneration. *Journal of Molecular Biology*.

[536] Amm I., Sommer T., Wolf D. H. (2014). Protein quality control and elimination of protein waste: the role of the ubiquitin-proteasome system. *Biochimica et Biophysica Acta (BBA) - Molecular Cell Research*.

[537] McKinnon C., de Snoo M. L., Gondard E. (2020). Early-onset impairment of the ubiquitin-proteasome system in dopaminergic neurons caused by *α*-synuclein. *Acta Neuropathologica Communications*.

[538] Betarbet R., Sherer T. B., Greenamyre J. T. (2005). Ubiquitin-proteasome system and Parkinson’s diseases. *Experimental Neurology*.

[539] Le W. (2014). Role of iron in UPS impairment model of Parkinson’s disease. *Parkinsonism & Related Disorders*.

[540] Shen Y. F., Tang Y., Zhang X. J., Huang K. X., le W. D. (2013). Adaptive changes in autophagy after UPS impairment in Parkinson’s disease. *Acta Pharmacologica Sinica*.

[541] Shin W. H., Park J. H., Chung K. C. (2020). The central regulator p62 between ubiquitin proteasome system and autophagy and its role in the mitophagy and Parkinson’s disease. *BMB Reports*.

[542] Pohl C., Dikic I. (2019). Cellular quality control by the ubiquitin-proteasome system and autophagy. *Science*.

[543] Kocaturk N. M., Gozuacik D. (2018). Crosstalk between mammalian autophagy and the ubiquitin-proteasome system. *Frontiers in Cell and Development Biology*.

[544] Kors S., Geijtenbeek K., Reits E., Schipper-Krom S. (2019). Regulation of proteasome activity by (post-)transcriptional mechanisms. *Frontiers in Molecular Biosciences*.

[545] Martinez A., Lopez N., Gonzalez C., Hetz C. (2019). Targeting of the unfolded protein response (UPR) as therapy for Parkinson’s disease. *Biology of the Cell*.

[546] Lin J. H., Li H., Yasumura D. (2007). IRE1 signaling affects cell fate during the unfolded protein response. *Science*.

[547] Silva R. M., Ries V., Oo T. F. (2005). CHOP/GADD153 is a mediator of apoptotic death in substantia nigra dopamine neurons in an in vivo neurotoxin model of parkinsonism. *Journal of Neurochemistry*.

[548] Valdes P., Mercado G., Vidal R. L. (2014). Control of dopaminergic neuron survival by the unfolded protein response transcription factor XBP1. *Proceedings of the National Academy of Sciences of the United States of America*.

[549] Sado M., Yamasaki Y., Iwanaga T. (2009). Protective effect against Parkinson’s disease-related insults through the activation of XBP1. *Brain Research*.

[550] Egawa N., Yamamoto K., Inoue H. (2011). The endoplasmic reticulum stress sensor, ATF6*α*, protects against neurotoxin-induced dopaminergic neuronal death. *The Journal of Biological Chemistry*.

[551] Selvaraj S., Sun Y., Watt J. A. (2012). Neurotoxin-induced ER stress in mouse dopaminergic neurons involves downregulation of TRPC1 and inhibition of AKT/mTOR signaling. *The Journal of Clinical Investigation*.

[552] Cooper J. F., Machiela E., Dues D. J., Spielbauer K. K., Senchuk M. M., van Raamsdonk J. M. (2017). Activation of the mitochondrial unfolded protein response promotes longevity and dopamine neuron survival in Parkinson’s disease models. *Scientific Reports*.

[553] Wang S., Horn P. J., Liou L. C. (2013). A peroxisome biogenesis deficiency prevents the binding of alpha-synuclein to lipid droplets in lipid-loaded yeast. *Biochemical and Biophysical Research Communications*.

[554] Yakunin E., Moser A., Loeb V. (2010). Alpha-synuclein abnormalities in mouse models of peroxisome biogenesis disorders. *Journal of Neuroscience Research*.

[555] Berger J., Dorninger F., Forss-Petter S., Kunze M. (2016). Peroxisomes in brain development and function. *Biochimica et Biophysica Acta (BBA) - Molecular Cell Research*.

[556] Burbulla L. F., Schelling C., Kato H. (2010). Dissecting the role of the mitochondrial chaperone mortalin in Parkinson’s disease: functional impact of disease-related variants on mitochondrial homeostasis. *Human Molecular Genetics*.

[557] Jo D. S., Park S. J., Kim A. K. (2020). Loss of HSPA9 induces peroxisomal degradation by increasing pexophagy. *Autophagy*.

[558] Zhang J., Tan L. C.-S. (2016). Revisiting the medical management of Parkinson’s disease: levodopa versus dopamine agonist. *Current Neuropharmacology*.

[559] Kalia S. K., Sankar T., Lozano A. M. (2013). Deep brain stimulation for Parkinson’s disease and other movement disorders. *Current Opinion in Neurology*.

[560] Barker R. A., Gotz M., Parmar M. (2018). New approaches for brain repair-from rescue to reprogramming. *Nature*.

[561] Fan Y., Winanto, Ng S. Y. (2020). Replacing what’s lost: a new era of stem cell therapy for Parkinson’s disease. *Translational Neurodegeneration*.

[562] Tufekci K. U., Civi Bayin E., Genc S., Genc K. (2011). The Nrf2/ARE pathway: a promising target to counteract mitochondrial dysfunction in Parkinson’s disease. *Parkinson’s Disease*.

[563] Minhas R., Bansal Y., Bansal G. (2020). Inducible nitric oxide synthase inhibitors: a comprehensive update. *Medicinal Research Reviews*.

[564] Stoker T. B., Torsney K. M., Barker R. A. (2018). Emerging treatment approaches for Parkinson’s disease. *Frontiers in Neuroscience*.

[565] Axelsen T. M., Woldbye D. P. D. (2018). Gene therapy for Parkinson’s disease, an update. *Journal of Parkinson’s Disease*.

[566] Parmar M. (2018). Towards stem cell based therapies for Parkinson’s disease. *Development*.

[567] Wang Z., Gao G., Duan C., Yang H. (2019). Progress of immunotherapy of anti-*α*-synuclein in Parkinson’s disease. *Biomedicine & Pharmacotherapy*.

[568] Malagelada C., Jin Z. H., Jackson-Lewis V., Przedborski S., Greene L. A. (2010). Rapamycin protects against neuron death in in vitro and in vivo models of Parkinson’s disease. *The Journal of Neuroscience*.

[569] Li X. Z., Chen X. P., Zhao K., Bai L. M., Zhang H., Zhou X. P. (2013). Therapeutic effects of valproate combined with lithium carbonate on MPTP-induced parkinsonism in mice: possible mediation through enhanced autophagy. *The International Journal of Neuroscience*.

[570] Bove J., Martinez-Vicente M., Vila M. (2011). Fighting neurodegeneration with rapamycin: mechanistic insights. *Nature Reviews Neuroscience*.

[571] Moors T. E., Hoozemans J. J. M., Ingrassia A. (2017). Therapeutic potential of autophagy-enhancing agents in Parkinson’s disease. *Molecular Neurodegeneration*.

[572] Savolainen M. H., Richie C. T., Harvey B. K., Männistö P. T., Maguire-Zeiss K. A., Myöhänen T. T. (2014). The beneficial effect of a prolyl oligopeptidase inhibitor, KYP-2047, on alpha-synuclein clearance and autophagy in A30P transgenic mouse. *Neurobiology of Disease*.

[573] Scrivo A., Bourdenx M., Pampliega O., Cuervo A. M. (2018). Selective autophagy as a potential therapeutic target for neurodegenerative disorders. *The Lancet Neurology*.

[574] Baltazar G. C., Guha S., Lu W. (2012). Acidic nanoparticles are trafficked to lysosomes and restore an acidic lysosomal pH and degradative function to compromised ARPE-19 cells. *PLoS One*.

[575] Bourdenx M., Daniel J., Genin E. (2016). Nanoparticles restore lysosomal acidification defects: implications for Parkinson and other lysosomal-related diseases. *Autophagy*.

[576] Issa A. R., Sun J., Petitgas C. (2018). The lysosomal membrane protein LAMP2A promotes autophagic flux and prevents SNCA-induced Parkinson disease-like symptoms in the *Drosophila* brain. *Autophagy*.

